# Interpreting alignment-free sequence comparison: what makes a score a good score?

**DOI:** 10.1093/nargab/lqac062

**Published:** 2022-09-05

**Authors:** Martin T Swain, Martin Vickers

**Affiliations:** Department of Life Sciences, Aberystwyth University, Penglais, Aberystwyth, Ceredigion, SY23 3DA, UK; The John Innes Centre, Norwich Research Park, Norwich NR4 7UH, UK

## Abstract

Alignment-free methods are alternatives to alignment-based methods when searching sequence data sets. The output from an alignment-free sequence comparison is a similarity score, the interpretation of which is not straightforward. We propose objective functions to interpret and calibrate outputs from alignment-free searches, noting that different objective functions are necessary for different biological contexts. This leads to advantages: visualising and comparing score distributions, including those from true positives, may be a relatively simple method to gain insight into the performance of different metrics. Using an empirical approach with both DNA and protein sequences, we characterise different similarity score distributions generated under different parameters. In particular, we demonstrate how sequence length can affect the scores. We show that scores of true positive sequence pairs may correlate significantly with their mean length; and even if the correlation is weak, the relative difference in length of the sequence pair may significantly reduce the effectiveness of alignment-free metrics. Importantly, we show how objective functions can be used with test data to accurately estimate the probability of true positives. This can significantly increase the utility of alignment-free approaches. Finally, we have developed a general-purpose software tool called KAST for use in high-throughput workflows on Linux clusters.

## INTRODUCTION

The rapid growth in genomic data sets poses problems for sequence search and comparison. The most frequently used methods for sequence comparison are based on sequence alignment. They include very well established methods such as BLAST (1), and algorithms like the Needleman-Wunsch (2) or the Smith-Waterman algorithm (3). Alignment-free methods have received less attention, nonetheless they have been a consistently active area of research over the last few decades (4). Alignment-free methods include a number of different approaches, and one of their main attractions is that they can scale more efficiently than alignment-based approaches. They may also be able to detect existing relationships between sequences that are overlooked by alignment-based approaches (5,6). Alignment-free methods have been applied to quite a diverse range of applications, including phylogenetic analysis (7); binning reads for metagenomic analyses (8); epigenetics applications (9); RNA-Seq quantification (10); anchoring vertebrate genome assemblies onto reference genomes (11); bacterial genome annotation (12) and protein classification problems (13).

### Overview of alignment-free methods based on *k*-mer frequencies

Many different alignment-free scoring methods are described in the literature and they have been the subject of a number of recent reviews. In (5), a number of applications of alignment-based methods are identified and they highlight areas where their application can be troublesome; while in (6) a survey, explanation and evaluation of many different alignment-free approaches is provided. Further authors have attempted to benchmark the different methods (14,15). We refer the reader to these excellent studies for further detail on different alignment-free methods.

In this paper, we have attempted to restrict our focus to methods that are based on *k*-mer frequencies, as we explain below. We have been motivated to understand how to interpret the outputs of these methods, i.e. their scores; to gain insight into why they might fail, and the situations when they are most successful. Here we provide a brief overview of the rationale behind the alignment-free metrics we have explored in this paper, and mention some of the previous analytical studies that have interpreted and characterised their score distributions.

One of the best characterised alignment-free scoring approach is the D2 metric or D2 statistic (16). The D2 metric can be defined by considering a pair of sequences comprised of a reference *r* and query *q* sequence that can be decomposed into DNA words typically called k-mers of a specified length *k* in base pairs. For example, if *k* = 4 then a DNA sequence of 4 letters A, C, G, and T can be decomposed into 4^*k*^ = 4^4^ = 256 possible words or 4-mers (GGCT or AAGA are examples of 4-mers). The D2 statistic is a sum over all possible *k*-mers, applied to the product of counts for a k-mer $w$ in the two sequences i.e.:

(1) $$\begin{equation*} D2(r,q) = \sum ^w{C_r(w).C_q(w)} \end{equation*}$$

Here, *C*_*r*_ and *C*_*q*_ are the counts (number of occurrences) of *k*-mers in the sequences *r* and *q*, respectively. Because this statistic is based on counts, it can have an arbitrary value that will tend to increase with the length of the sequences under consideration. Various modifications have been proposed to the *D*2 metric to give it a more practical value, e.g. to scale the scores output from the metric so that they lie between 0 and 1, where lower scores represent more similar sequence pairs. For instance, the vector cosine distance can be written as:

(2) $$\begin{equation*} cosine(r,q) = \frac{D2}{\sqrt{\sum {C_r(w)}^2}{\sqrt{\sum {C_q(w)}^2}}} \end{equation*}$$

Further modifications may then be applied to the vector cosine metric by different authors to obtain more convenient scores. For instance, in the *D*2 metric rather than giving a value between –1.0 and 1.0, it may be adjusted to give a number between 0.0 and 1.0.

The frequency of occurrence of each *k*-mer is often used instead of the counts. *K*-mer frequencies have been shown to generate signatures unique to different organisms (17–19). The k-mer frequencies are derived from the counts by dividing each *k*-mer count by the total number of *k*-mers in the sequence (i.e. the sequence length minus the *k*-mer length). The metrics we have investigated in this paper are based on k-mer frequencies. Once the frequency vectors have been calculated, various distance measures can be used to estimate similarity, which include the Euclidian, Manhattan, Canberra or Chebyshev distances (6). Other metrics discussed in the literature may be tweaked in different ways by different authors, and as they may be given different names, it can be a challenge to clearly distinguish between different metrics.

A similar approach to the *D*2 metric is used to find alignment seeds in alignment software like BLAST (1) and BLAT (20). The *D*2 metric has therefore been subjected to relatively rigorous and sophisticated analyses (21–23). Although the metric is relatively simple to implement, data sets of biological sequences can be complex and the application of *D*2 is not always straightforward to interpret. Lippert *et al.* (21) investigated the properties of the *D*2 distribution using an analytical approach. They found that depending on the k-mer length and *k*-mer abundance in the sequences under consideration, the properties of the *D*2 distribution change. They identified three asymptotic regimes including the compound Poisson and normal distributions. Foret *et al.* (24) proposed that the gamma distribution may be better than the normal distribution for characterising the *D*2 distribution for applications such as the analysis of EST sequences. By comparing analytical and empirical distributions, these authors investigated estimates of significance in the scenario of database search, where a query sequence is compared to several other sequences, and the *P*-value is then estimated for the best of these comparisons. It is worth noting that every distribution will contain rare or significant scores so long as there is variation between the sequences under study. These significant scores, however, do not necessarily correspond to biological significance because the metric may be unable to distinguish signal, in the sense of biological features of subjective interest to the researcher, from noise i.e. uninteresting features. Furthermore, the D2 distributions can be complex to characterise due to the natural variability of the sequences under consideration, i.e. statistical noise arises from random variation in each sequence as well as due to correlations of *k*-mer frequencies in both sequences (25,26). Lippert *et al.* concluded their study with the observation that ‘a naive, one distribution fits all, approach to *D*2 statistics could easily create serious errors in estimating significance’ (21).

To address some of these issues, modifications to *D*2 were implemented in approaches such as d2Star and d2S (also known as d2Shepp) (23,25). These two metrics use a Markov approach to estimate the likely occurrence of various words (27). For example, they might use the background, i.e. the frequency of occurrence of 1-mers (the four letters A, C, G and T) in a DNA sequence to calculate an expectation value of how abundant each of the possible 4-mers should be in that sequence. These expectation values are used to adjust the actual abundance of 4-mers measured by the counts, and subsequently the frequencies. They adjust the *D*2 metric and its distribution in various ways that may yield greater accuracy. There is no equivalent background for protein sequences, because proteins are the functional molecules that DNA codes for, with different pressures acting on the amino acids that comprise the sequence. Moreover, the protein alphabet is large, and protein sequences are short, meaning that the background cannot be calculated accurately.

Two further metrics that we have investigated are derived from areas other than biological sequence analysis (28,29). The Bray–Curtis metric was designed for ecological applications, to measure dissimilarity between two different sites in terms of the species found in those two locations (30). The Bray–Curtis dissimilarity can be written, when comparing two sites *i* and *j*, as:

(3) $$\begin{equation*} {\rm BC}_{i,j} = 1 - \frac{2C_{i,j}}{S_i + S_j} \end{equation*}$$

where *C*_*i*, *j*_ is a sum of the species (i.e. *k*-mers) common to both sites (i.e. sequences), with the sum using whichever species count is the lesser between the two sites; the *S*_*i*_ and *S*_*j*_ are the total counts of species at each site. An important assumption here is that the two sites are of a similar size. The Google similarity distance is used to find semantic similarity in web pages. It implements the idea that related key words with similar meanings should be ‘close’ according to this measure (31). It may be modified to compare protein sequences (32) by summing over key words (i.e. k-mers) to give the Normalised Google Distance:

(4) $$\begin{equation*} {\rm NGD}_{i,j} = \frac{ {\rm max}(S_i,S_j) - C_{i,j} }{(S_i + S_j) - {\rm min}(S_i,S_j)} \end{equation*}$$

Here, the max(*S*_*i*_, *S*_*j*_) and min(*S*_*i*_, *S*_*j*_) are the greater value between *S*_*i*_ and *S*_*j*_, or lesser, respectively. When formulated this way it performs in a similar manner to the Bray-Curtis dissimilarity.

### Differences between alignment-based and alignment-free sequence comparison

Our paper is motivated by the desire to understand the application of alignment-free methods in practice, for instance as an alternative approach that can complement well established genomics workflows based on sequence alignment. A key observation we have made concerning differences between alignment-based or alignment-free approaches is that, with sequence alignment, the input sequences are preserved and may be recovered from the output (i.e. the alignment), thus preserving biological context; but this is not true for alignment-free methods.

On one hand, sequence alignments are often very large and complex data sets. They may incorporate thousands or even millions of sequences and may be used for a range of sophisticated applications including predicting protein structure or elucidating evolutionary patterns (33).

On the other hand, when using an alignment-free metric, the result of comparing two sequences is nothing more than a number, i.e. the similarity score. It is important to realise that at this stage all knowledge of the input sequences has been lost: for a single pairwise comparison the similarity score has no biological meaning at all. It is only when data sets consisting of three or more sequences are processed that the alignment-free scores can provide some biological insight.

For example, for a set of three sequences (A, B and C), the relative difference in the scores might indicate that sequence pair A and B are more similar than sequence pair A and C. Alignment-free metrics, however, do not provide an objective measure of similarity such as, for instance, the *E*-value from BLAST – instead they only measure relative similarity within the set of sequences being searched. The principle output from alignment-free search is therefore the ranking of the similarity scores (in other words, the ranking of sequences in the reference data set in terms of their measured similarity to the query sequence). As we highlight in this paper, each metric measures ‘similarity’ in different ways, sometimes with significantly different assumptions and biases.

### The use of objective functions to encode biological context

We propose that an objective (or evaluation) function should be applied to replace the biological context that has been lost in alignment-free search. This function can be used to map the pair of sequence identifiers associated with a score onto external knowledge bases that provide biological context. Using the knowledge-bases, it will encode a particular set of criteria relevant to the specific search problem performed by the researcher, thus allowing sequence pairs that are true positives to be identified within that domain. Without an objective function, it is still possible to generate a distribution of scores, but there would be no basis to identify true positives within that distribution and so no basis to evaluate how effective the search algorithm is. We demonstrate how an objective function can be used to calibrate alignment-free metrics for different applications using test data. As a result of this process, similarity scores can be mapped onto likelihood values, indicating the probability of a correct prediction.

We have performed empirical studies to characterise the properties of the score distributions generated by a number of different alignment-free scoring metrics that use k-mer frequencies. We have used objective functions to identify true positives within those score distributions. The advantage of our empirical approach is that it is relatively simple to implement and it does not require the specialised statistical background that may be required to engage with some of the analytical approaches described above. Moreover, it may be readily applied to both nucleotide and protein sequence.

### The need for a general-purpose tool for alignment-free sequence comparison

To aid our investigations we developed the software KAST. This was needed due to the lack of other suitable alignment-free software that could process both DNA and protein sequences. KAST was designed for efficient execution and to provide suitably flexible input/output formats. This allowed us to embed it in data analysis pipelines so that we could explore a variety of practical investigations. It is designed to be a general-purpose tool for alignment-free sequence comparison. While a number of software packages exist (5), they tend to be designed for specific research purposes rather than general usage. Many are implemented in high level scripting languages that give relatively slow execution times; or they are embedded in frameworks designed to compare and contrast different metrics and are awkward to use in external applications.

## MATERIALS AND METHODS

Here we describe the data sets, objective functions, and analyses that we have performed. We have split this into two sections, one for amino acid (i.e. protein) sequence and the other for nucleotide sequence. The list of metrics we have investigated is given in Table 1.

**Table 1.** The alignment-free metrics investigated. The ‘y’ or ‘–’ in a column denotes whether or not the metric was used in the protein (AA) or nucleotide (DNA) analyses (metrics incorporating a Markov approach where not applied to protein analyses because the background is ill-defined in these cases), if the metric is suitable for use with sequences of variable length (Len), and finally a reference for the metric is given. Note the Markov methods d2Star and d2S are calculated with the m=0 parameter in KAST, which sets the background k-mer length to 1 bp

Metric (and acronym)	AA	DNA	Len	Ref
*D*2	y	y	y	(6)
Euclidian distance (euclid)	y	y	y	(6)
Manhattan distance (manh)	y	y	y	(6)
Chebyshev distance (cheby)	y	y	y	(6)
Normalised Canberrra distance (ncamb)	y	y	–	(6)
d2Star	–	y	y	(23,25)
d2Shepp (d2s)	–	y	y	(23,25)
Brays-Curtis (bc)	y	y	–	(30)
Normalised Google Distance (ngd)	y	y	–	(31,32)

### Data sets and objective functions for predicting orthologs from protein sequence

We used data sets from DIOPT (34) to develop an objective function for protein ortholog prediction. Different tools for predicting orthologs can give different results: to overcome this, DIOPT integrates a nine existing approaches and demonstrates an increased sensitivity with only a modest decrease in specificity (34). DIOPT includes inparalog and co-ortholog predictions, and it is not restricted to one-to-one ortholog relationships, although these are the majority. The species and data we investigated were: *Schizosaccharomyces pombe* or fission yeast with 5138 proteins (35); *Saccharomyces cerevisiae* or brewer’s yeast with 6,713 proteins (36); *Caenorhabditis elegans* with 28 400 proteins (37); and *Drosophila melanogaster* with 30,493 proteins (38). See also Table 2.

**Table 2.** Statistics describing the original data sets used. The range of sequence lengths is given by the min(imum) and max(imum) values. The *C. elegans* and *D. melanogaster* data sets contain a few outliers (very long protein sequences): most of the sequences are less than about 6,000 amino-acids, respectively

Data set	Min	Max	Mean	Mode
*S. pombe*	24	4,924	464	378
*S. cerevisiae*	17	4,911	450	359
*C. elegans*	9	15 188	467	340
*D. melanogaster*	11	22 949	659	468
Species genomes	138 927	13 654 608	3 383 082	3 018 238
Strain genomes	6847	10 236 779	3 018 845	2 736 403

Sets of protein sequences were downloaded for each of these species and the FASTA headers processed so that the identifiers could be mapped to the data stored in DIOPT (DIOPT provides gene identifiers rather than protein identifiers). Tables of orthologs could then be retrieved from DIOPT. Finally, the DIOPT data sets were adjusted so that the identifiers used by DIOPT matched those in the FASTA headers of the protein sequences. Thus, it was possible to map between the FASTA headers and the ortholog data from DIOPT.

The evaluation process involved running the alignment-free sequence comparison with sets of protein sequences from two different species (e.g. yeast species Y1 and Y2). A protein from species Y1 was compared against all the proteins from species Y2 using a single alignment free metric. The scores were ranked. Then, using the objective function, a positive prediction was called if the top ranked pair of proteins was present in the set of DIOPT orthologs, otherwise it was a false prediction. The process was repeated for every protein in species Y1 so that the number of correctly predicted orthologs could be counted. This was done in two stages: first we perform the alignment-free comparison on all the proteins from species Y1 using the KAST software (presented later in this paper), then we evaluate using an objective function that counted the correct predictions using Python scripts. True orthologs were counted if the DIOPT evidence was one of ‘high’, ‘moderate’ or ‘low’.

### Investigations performed on protein sequences

The following investigations of predictive accuracy were performed. In each of these tests, all the sequences from one FASTA file are compared against all sequences in another FASTA file. This results in 5138 × 6713 = 34, 491, 394 comparisons for the fission yeast versus brewer’s yeast case. For the fly-worm case, the numbers get large and time-consuming, especially for the larger *k*-mer lengths. We therefore used subsets of this data, including 1/10th and 1/30th of the data for *D. melanogaster*. For instance when the 1/30th data set is used (1107 proteins) with all the data from *C. elegans* (28 400 proteins) this results in 28 882 800 comparisons, a similar number to the yeast system.

#### The influence of k-mer length (proteins)

We investigated this by evaluating the different metrics in two different ways:

1. By using the top hit only, i.e. the optimal ranked pair of proteins based on the scores.
2. By generating the receiver-operator curves (ROC) and calculating the area under the curve or AUC.

#### Distributions: histograms of score frequencies (proteins)

The frequency of occurrence of different scores for a variety of metrics and k-mers was explored. If the maximum and minimum possible scores fall between 0 and 1 (which it does for most of the metrics) then that score range might be divided into 100 bins and the number of scores falling into each bin counted. The score frequency for a histogram bin is calculated by dividing each of the 100 bin counts by the total number of scores (i.e. the sum over all bins). The histograms indicate how the scores distribute over the range of allowed scores. Two different types of score frequency histograms were calculated:

1. All-scores distribution: this is when all sequences in the two species are compared to each other and consists of both positive and negative scores. Negative scores dominate the frequency values in this histogram because they vastly outnumber the positive scores. For example for the 34 491 394 comparisons made in the two yeast case, there are 7090 pairs that are classified as true orthologs by DIOPT (DIOPT includes, e.g. many-to-one ortholog mappings).
2. Positive-only distribution: this distribution is based on the set of sequence pairs corresponding to true positive scores according to DIOPT. Only unique pairs were included (i.e. one-to-one mappings), meaning that if a protein from the first species of the pair mapped to multiple proteins in the second species, then only the first such ortholog mapping was included. It consists of 4409 pairs for the yeasts system. Note that this distribution is a subset of the all-scores distribution.

#### Overlaps between distributions (proteins)

We have used the histograms of score frequencies as a basis to empirically investigate and compare the behaviour of various scoring metrics and parameters. To summarise these results into a single figure, we calculated the fraction of overlap between two score histograms. This has previously been described as the overlapping index (39): for two distributions *A* and *B*, the overlapping index η is given by the sum, over all scores *s*, of whichever distribution has the minimum score-frequency *f*, i.e.

$$\begin{equation*} \eta (A,B) = \int _{s=0}^{max(s)} min[f_A(s),f_B(s)] \, ds \end{equation*}$$

For example, consider the overlapping index between the positive-only and the all-scores histogram: if the positive-only and the all-scores distributions overlap each almost entirely, the overlapping index has a value close to 1.0, and the predictive accuracy is expected to be low; on the contrary, if there is relatively little overlap (the overlapping index is close to 0.0) then the predictive accuracy is expected to be high.

#### Score correlations with length (proteins)

We have calculated correlations between the length of the protein sequences (the mean of the pair) and the score, to investigate if these are independent from each other. This was performed in R version 3.1.2 (40) using the Spearman rank correlation for nonparametric data. The data set used was a set of 5000 true orthologs (positives) from the fly-worm system.

#### Determining the likelihood of a prediction being correct for protein ortholog predictions

We calculate the cumulative difference in score frequency between two score histograms, i.e. that of the positive-only and the all-scores. This can be used with an objective function to identify a specific score that maximises positive scores whilst allowing most negative scores to be filtered out. For instance, the difference between the curves is calculated by iterating over each of the bins in the two histograms that correspond to the same scores, and subtracting the positive-only score frequencies from those from the all-scores. The sum of these differences gives the cumulative difference curve and the maximum of this curve gives a score that may be used as a cut-off score. To map scores from pairwise comparisons onto fractional values indicating the likelihood of a prediction being correct, we have taken the output from KAST and stored the highest ranked sequence pairs and their scores. By iterating over the range of possible scores, usually from 0.0 to 1.0, it is possible to quickly evaluate predictive accuracy at a range of different scores. This allows the selection of a score that gives a desired level of predictive accuracy – assuming that only the top ranked score is of interest. We performed this using the fly-worm system with 1/10th of the *D. melanogaster* data.

### Data sets and objective functions for predicting taxonomic assignment from DNA sequence

To develop an objective function for taxonomic assignment with DNA sequences, we used the data set generated by Genometa of whole (or nearly whole) genome sequences (41). A number of quality checks were performed on this data set that reduced its size. The remaining data set consists of 1705 sequences that are split into two portions. One portion contains one genome per species and consists of 1052 sequences—we refer to this as the species or reference data set. There are 1047 unique taxonomic species in this data set (there are redundancies due to e.g. heterotypic synonyms). The second portion consists of 653 strain sequences, of which 493 (75.5%) map onto the species sequences in the reference data set – we refer to this as the strain or query data set. A number of the 493 strains map onto the same species: in total there are 187 unique taxonomic species in the set of 493 strain sequences. See also Table 2.

In order to evaluate taxonomic comparisons between these two data sets taxonomic information was downloaded from the NCBI taxonomy database (42) and an in-house script was used to parse this information and build the taxonomic tree. A file was produced that maps the GI numbers in the Genometa FASTA headers to the following 6 attributes of taxonomic information, in order of the lowest member of the hierarchy to the highest: strain, species, genus, family, order, class, and phylum. This means that sequences in the strain data set can be mapped onto the species data set using the NCBI taxonomic identifiers – and this can be used to evaluate taxonomic predictions made by alignment-free comparison methods. Note that although sequences in the reference data set were identified down to the strain taxonomic level, those strains (as identified by the taxonomic identifier) were not contained within the query data set. This means that all matches between sequences from the query and reference data sets occur at the species taxonomic level or higher.

The evaluation process involved using alignment-free metrics to score similarity between sequences from the strain data set when compared to sequences from the species data set. Similar to the protein case, one sequence at a time from the strain data set was compared against all sequences in the species data set. The scores were ranked. The objective function called a positive prediction if the sequence identifiers belonging to the top ranked pair of sequences mapped to the same taxonomic level, e.g. the species or genus level. After performing all pairwise sequence comparisons using the KAST software, the correct taxonomic predictions were counted using the objective function.

We investigated a number of different parameters using the following data sets. Fragments of different sequence lengths were extracted from each of the species (reference) and strain (query) data sets. Five fragments of the length *L* were created by dividing the genome sequence into ∼10 equal sized intervals. To avoid overlaps between reference and query fragments, and to sample along the length of the genome, the five query and reference fragments were taken from alternating intervals. This was to reduce biases in the data that may arise because many genome sequences are recorded from the origin of replication: they may therefore be considered to be semi-aligned, and this can significantly inflate the accuracy achieved with the shortest fragments. Each fragment was created by taking the first *L* bases from each of those intervals, where *L* was initially set to 100 bp, and then doubled until reaching 102,400 bp. In order to reduce the complexity of our results, only fragments of length *L* equal to 0.1, 0.4, 1.6, 6.4, 25.6 and 102.4 kb were included in the figures. Thus for a fragment size of 0.1 kb there are 5 × 1052 = 5260 sequences in the species data set, and 5 × 653 = 3265 in the strain data set; and when these data sets are compared to each other 17 173 900 comparisons are made. There may be slightly less sequences in the data sets for longer *L* because a few of the genome sequences were incomplete and of insufficient length to generate the required fragments.

### Investigations performed on DNA sequences

We investigated how the following parameters affect predictive accuracy.

#### The influence of k-mer length (DNA)

and how this may change with different sequence lengths, for different metrics.

#### The influence of sequence length (DNA)

This was investigated in the following ways:

1. *L*-equal: here the reference and query sequences are of equal length. There are six different data sets corresponding to the six different fragment lengths *L*.
2. *L*-unequal: here the query sequences are allowed to vary over the six different fragment lengths but the reference query is fixed at the first 250 000 bp. The original idea was to include the whole reference genome sequence, but this led to cumbersome data sets and little difference in performance. There are six different data sets corresponding to the six different query lengths *L*.

#### Distributions: histograms of score frequencies (DNA)

The calculation of this histogram is similar to that described under the equivalent paragraph for proteins. Two different types of score frequency histograms were calculated:

1. All-scores distribution (*L*-equal and *L*-unequal): this is when all sequences in the species and strain data sets are compared to each other and consists of both positive and negative scores. There is a different distribution for each of the 6 different fragment lengths *L*. Negative scores dominate the frequency values in this histogram because they vastly outnumber the positive scores. For example if 5 × 1052 species sequences of length 0.1 kb are compared against 5 × 653 strain sequences of length 0.1 kb this gives a total of 17 173 900 scores, of which 5 × 5 × 493 = 12 325 sequences would be true positive scores
2. Positive-only distribution (*L*-unequal only): this consists of the strain fragments of a particular fragment length (where *L* is 0.1 kb, 0.4 kb etc.) matched to the whole genome sequences in the species data set. Only the first 250 kb of the whole genome sequence was used to reduce file sizes and compute speed. A positive pair shared the same taxonomic identifier: using ‘species’ as the taxonomic identifier resulted in 5 × 493 = 2465 sequence pairs (consisting of 187 species). For comparison, using the ‘genera’ taxonomic identifier gives 22 300 matched sequence pairs (consisting of 121 genera that now encompass approx. 440 species). There is a different file for each of the 6 different fragment lengths *L*.

#### Overlaps between distributions (DNA)

We have used the histograms of score frequencies as a basis to empirically investigate the behaviour of various scoring metrics and input parameters. This is calculated in the same way as for proteins.

#### Determining the likelihood of a prediction being correct for taxonomic classification (DNA)

This is calculated in the same way as for proteins.

### The KAST software

KAST has been implemented using the SeqAn toolkit, a C++ template library for the analysis of biological sequences (43). Parallelization is implemented via the pthreads library. Data can be nucleotide or amino acid sequences, with an option for a 10 character reduced amino acid alphabet (44). One advantage of the increased efficiency is that it has allowed us to explore relatively long *k*-mers for amino-acid sequences.

We have designed a number of different ways to input data to enable flexibility in its usage. These include options to provide sets of query and reference sequences, giving similar usage to alignment software; an option to provide a single file for all against all sequence comparisons; and the ‘interleaved’ option where pairs of sequences in the input file are compared to each other – by creating such files externally the user has full control over which sequences are compared to each other. KAST supports 13 different formats for input data (e.g. FASTA, FASTQ, sam, bam, GBK, EMBL etc.) as well as gzipped versions. Output can be provided as a distance matrix (for use with phylogeny applications) or in a number of different tabular formats. The scores are ranked in the KAST outputs.

Currently over 10 different scoring metrics are implemented in KAST. These include the cosine metric; the d2 variant of the cosine metric; distance measures such as Canberra, Manhattan, Chebyshev, and Euclidian; the Bray-Curtis (30) dissimilarity and the Normlised Google Distance (31,32); and approaches based on the D2 statistic that incorporate Markov models, including d2Star, d2S (or d2Shepp) (23,25,27) and the S2 (or dAI) metric (27). The github documentation includes instructions on how to add more distance measures.

Each of the metrics can be used with binary masks to implemented gapped or spaced k-mers (45–47). For example, masking strings like ‘1010101’ and ‘1101100’ can be used (simultaneously) with a 7-mer. The masking string is mapped to the k-mer and only those base positions denoted a ‘1’ are included in the comparison metrics (those with a ‘0’ are skipped).

We have also added parameters to filter sequence pairs with dissimilar lengths, and to apply a cut-off score.

For the KAST distribution, please see the Data Availability section.

## RESULTS AND DISCUSSION

We first consider the performance of various metrics on protein ortholog prediction, then we consider their performance for the taxonomic classification of DNA fragments.

### Influence of K-mer length on predictive accuracy for protein ortholog prediction

**Figure 1.**
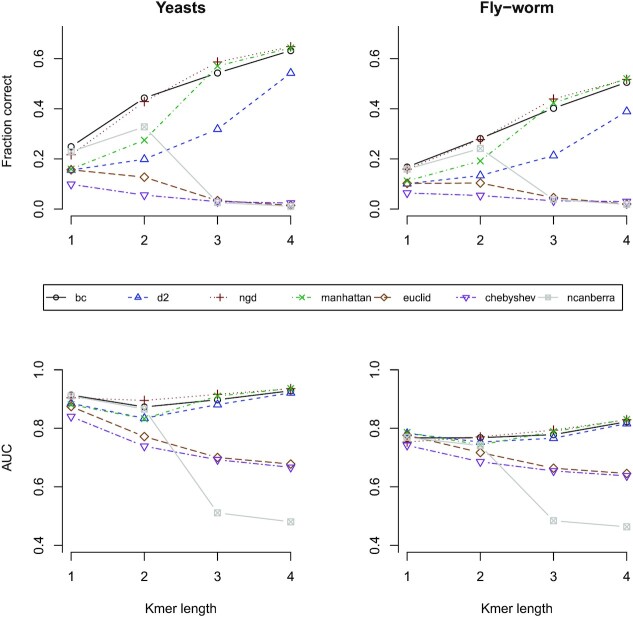
The fraction of correct protein ortholog predictions (based on top ranked hit) and AUC (area under the receiver-operator curve (ROC)) for the scoring metrics given in the key (centre of figure) with different *k*-mer lengths. ‘Yeasts’ involves *S. pombe* proteins compared to *S. cerevisiae*, and ‘Fly-worm’ involves *C. elegans* proteins compared to *D. melanogaster*.

In Figure 1, we have considered the prediction of protein orthologs. Here, we have used two systems: comparisons between two closely related species, two yeasts (*S. pombe*) and (*S. cerevisiae*); and comparisons between two much more distantly related species, a fly (*D. melanogaster*) and a worm (*C. elegans*). These data are described in the materials and methods. In Figure 1, we have explored seven different scoring metrics and *k*-mer lengths 1 to 4. Using k-mers of length 5 does not improve on the results shown here. We have measured the accuracy of ortholog prediction in two different ways: firstly, by considering whether or not the pair of sequences giving the highest ranked score is a true ortholog; and secondly by calculating the AUC or area under the curve for the receiver-operator curves (ROC curves). The data for this figure are given in Supplementary File 1.

The yeasts comparison tends to give more accurate results than the fly-worm comparisons, as might be expected due to the smaller evolutionary distance in the yeast system. With *k*-mer length 4 the fraction of correct predictions is 65% for NGD in the yeasts, and this falls to 52% for NGD in the fly-worm system. It is also worth noting that the fly-worm system involves identifying the correct ortholog out of a data set of 28 400 *C. elegans* proteins, whereas the this number is considerably smaller at 6713 for the yeasts.

Three of the metrics clearly show decreased accuracy for longer *k*-mers (Euclidian, Chebyshev and Canberra), while the other four metrics tend to show improved accuracy with longer *k*-mers. According to Figure 1, the scoring metrics based on the Euclidian and the Chebyshev distances both give poor performance in general for protein sequence, and their performance further declines with increasing *k*-mer length. This is in contrast to the Manhattan, NGD, BC and d2 metrics, and these four metrics behave in a similar manner to each other. In the yeasts, ortholog predictions based on the fraction correct increase from 16% to 25% for k-mer length 1, up to 54% to 65% for *k*-mer length 4 (with *D*2 giving slightly worse performance here).

The Canberra metric gives good performance for *k* = 1: here the AUC has a value of 0.911 in the yeast system and it compares favourably to the best metric with these parameters, which is BC with 0.913. Performance, however drops off significantly for the Canberra metric with larger *k*-mers, with the AUC falling to 0.480 and 0.463 (slightly worse than random) for *k* = 4, which compares to 0.928 and 0.822 for BC, in the yeast and fly-worm systems respectively.

There is a minimum in the AUC curve for most metrics at k-mer length 2. This is most pronounced for the *D*2 and Manhattan metrics in the yeasts comparison. This minimum of the AUC curve can be at least partially explained using factors that we explore in the subsequent subsections. This includes the fact that scores for the metrics are not independent of the length of sequences being compared, but instead may correlate with this parameter.

### Insights on protein ortholog prediction derived from the distributions of score frequency

**Figure 2.**
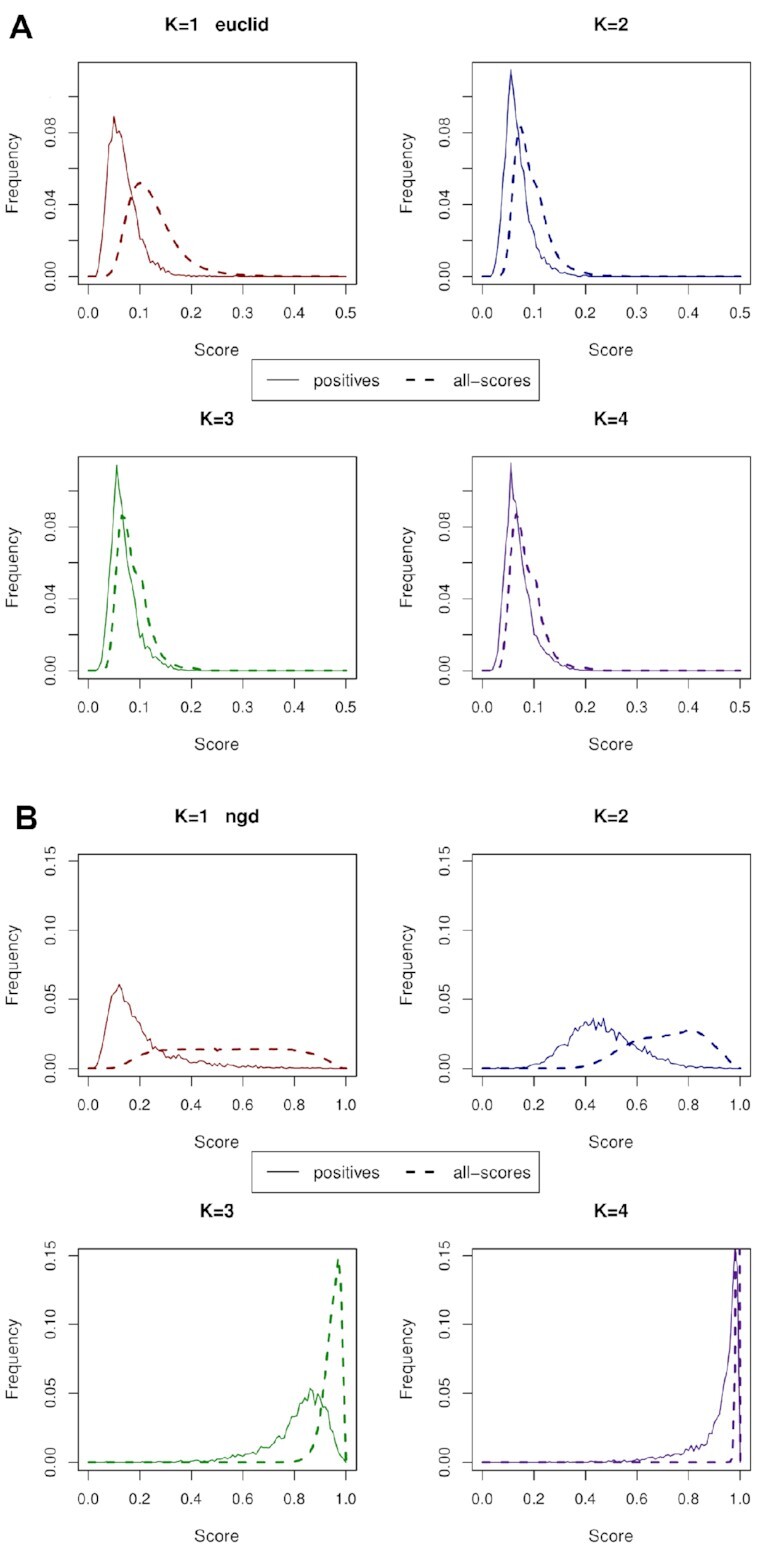
Score distributions showing the frequency of occurrence (y-axis) of each score (x-axis) for (**A**) the weakly performing Euclidian, and (**B**) strongly performing NGD metrics. *K*-mers ranging from 1 to 4 amino-acids are considered, with the distributions given for two data sets: (i) positives for the true positive-only scores (solid line); and (ii) all-scores (dashed line). These results are for the yeasts system. Note the overlap between the distributions increases for the Euclidian metric, for longer k-mers, while NGD retains significant separation between the distributions.

In Figure 2, we show distributions of score frequency, for all-scores and positive-only distributions with the Euclidian and NGD metrics, for *k*-mers 1 to 4. These metrics are shown because, of the metrics represented in Figure 1, NGD generally has the best performance and Euclidian is one of the worst. In particular, for the AUC curve, NGD has the highest value for *K* = 2, with the smallest dip in the curve at this point. When comparing the distributions for Euclidian, it is clear from Figure 2 that as the *k*-mer length increases there is much less difference between the ‘positives’ and ‘all-scores’ distributions. The overlapping index between the two distributions indicates how similar these distributions are. The more similar they are, the less successful the metric is at identifying signal from noise. Ideally, the true orthologs or ‘positives’ would have a distribution with minimal overlap to those of the ‘all-scores’.

In contrast to the Euclidian metric, the distributions for NGD have greater spread over the range of possible scores and the ‘positives’ and ‘all-scores’ distributions are more clearly distinguishable. Scores for *k*-mer length 1 tend to be distributed closer to 0.0, the bottom of the range of possible scores; while for *k*-mer length 4 the scores tend to be distributed close to 1.0, the top of the scale. There is also a relatively clear separation (i.e. a small degree of overlap) between frequency of score distributions for ‘all-scores’ and those for ‘positives’, across all *k*-mer lengths, and the separation increases slightly for longer k-mers. This corresponds to better predictive performance being achieved with longer k-mers.

Distributions for the other metrics are available as figures in the supplementary information, Supplementary File 2.

**Figure 3.**
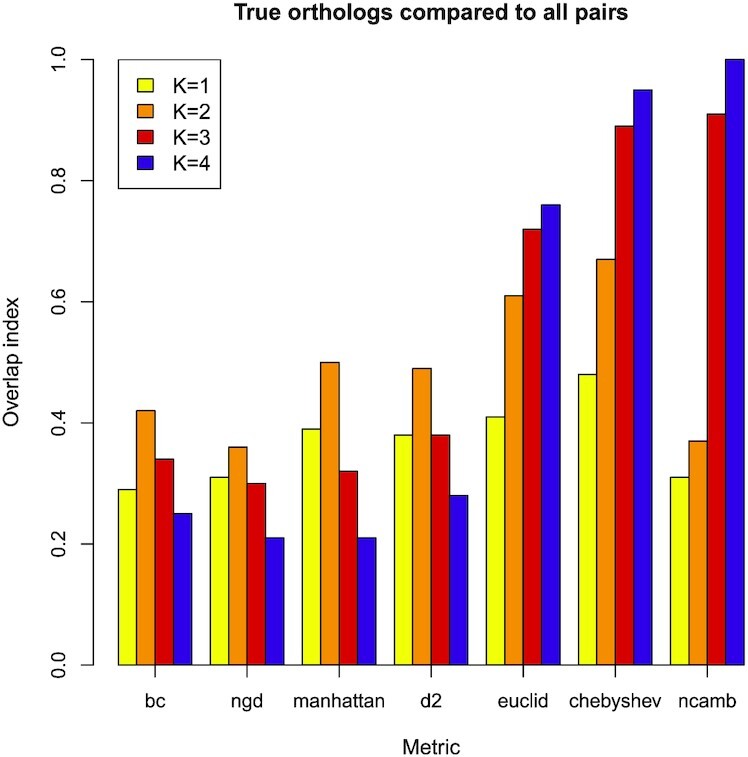
The overlapping index between the curves from the positive-only distribution (true protein orthologs) and the distribution of all-scores, for all protein pairs in the yeasts system. The smaller the overlap index, the better the predictive performance. The Euclidian, Chebyshev and Canberra metrics all give poor performance for *k*-mers 3 and 4 in particular.

In Figure 3, we have calculated the overlapping index between the ‘positives’ and the ‘all-scores’ distributions in order to summarise these findings for seven different metrics and four different k-mer lengths. The BC, NGD, d2 and Manhattan metrics all perform well according to the results shown in Figure 1 and they have relatively small overlaps in Figure 3. As explained previously, a relatively small fraction of overlap indicates better predictive performance than a relatively high overlap. For these metrics, the largest overlap is for *k*-mer length 2, which corresponds to the minimum of the AUC curve in Figure 1. The variation in the scores is relatively high for *k*-mer length 2, leading to distributions centred on the mid-point of the range of scores and which are spread over the full range of scores, e.g. from 0.0 to 1.0. These distributions have relatively large overlaps. As k-mer lengths increase, the score distributions tend to be pushed towards the extremes of the range of allowed scores, the distributions are relatively compressed, and the overlaps are smaller. The smallest overlap for these four metrics is for k-mer length 4, shown in purple in Figure 3, and giving the highest accuracy in Figure 1.

In contrast, the Euclidian, Chebyshev and Canberra metrics tend to have worse performance with larger *k*-mers. Figure 3 shows that there is large overlap between the ‘positives’ and ‘all-score’ distributions, meaning that there is little difference between the scores for known orthologous sequences and those without any detectable orthology. For these metrics, the best performance is achieved with k-mer length 1, where their overlapping index values are lowest.

### Does the similarity score correlate with mean protein sequence length?

**Figure 4.**
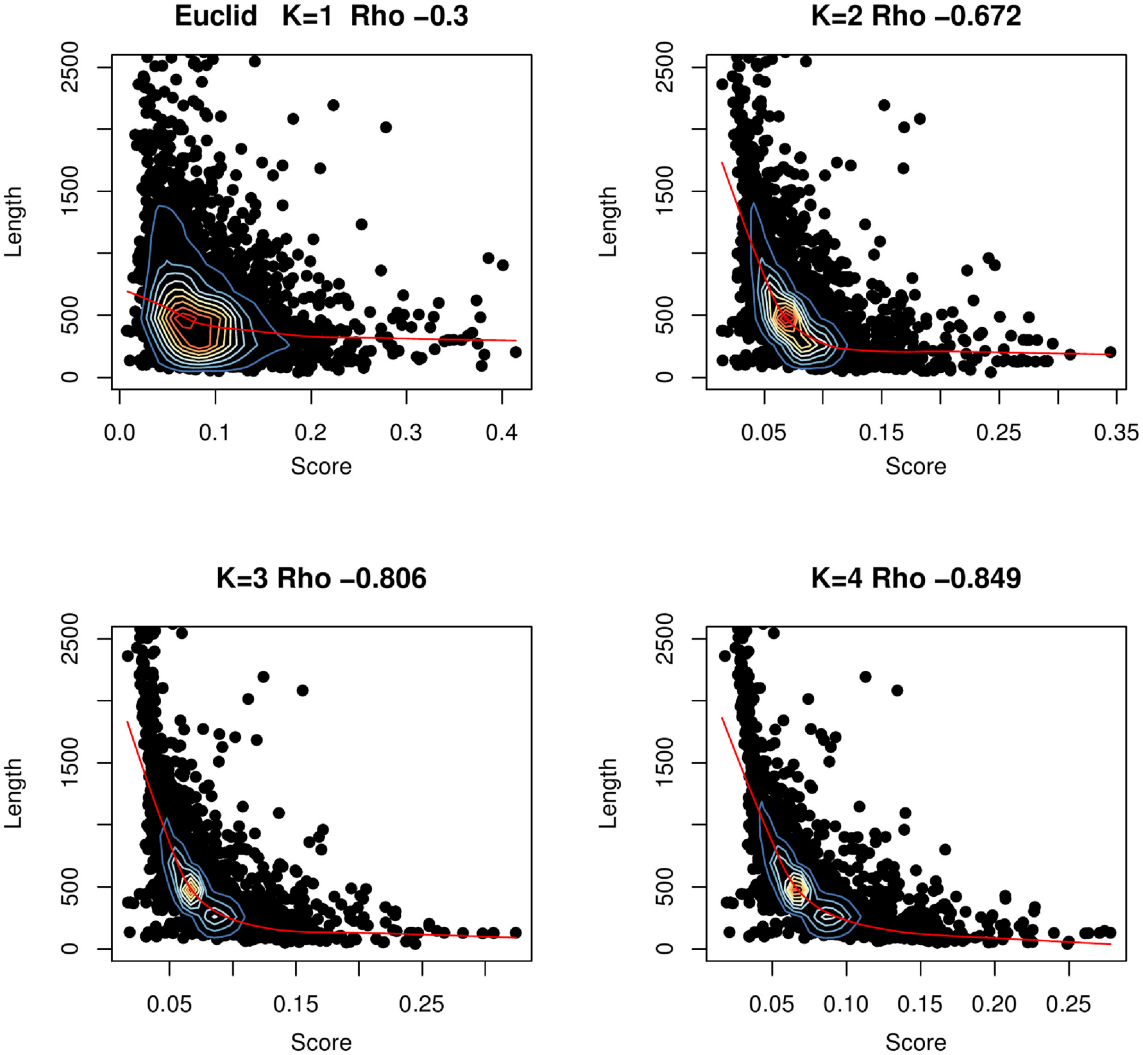
The correlation between mean protein sequence length and the score of true positive sequence pairs, for the weakly performing Euclidian metric with different *k*-mer lengths. The test was performed with Spearman’s rank correlation and the values of Rho are given in the figure. In each case, the *P*-value is below 2.2e–16. Contour lines indicate the density of the dots. Large correlations are observed for longer *k*-mers, where performance is weakest for this metric.

In Figure 4, we explore the relationship between protein sequence length (i.e. the mean length of the pair of sequences being compared) and the metric score for the ‘positives’ distribution. For the Euclidian metric, with *k*-mer lengths 1 to 4 we have used a dot plot to indicate the relationship between the mean sequence length and the score, for each protein pair. We have calculated the correlation between mean length and score using Spearman’s rank correlation. The correlation values are given in the figure legend and are weakly anti-correlated for k=1 and strongly anti-correlated with values below –0.8 for *k*-mers 3 and 4. Thus for the Euclidian metric, which gives poorer performance as the *k*-mer length increases, the scores for longer *k*-mers are dependent on protein sequence length. For longer *k*-mers, if the mean length of the protein pair is relatively short, then the score is likely to be high, predicting a relatively dissimilar pair; while if the mean length of the protein pair is relatively long, then the proteins are predicted to be relatively similar. Ideally, there should be zero correlation between score and sequence length.

These results are for the fly-worm system and figures for other metrics are given in Supplementary File 3. Similar effects are also seen in the yeasts system: although the magnitude of the correlation might change by around 25% or so, the pattern (relative magnitude of correlations) across the *k*-mer lengths remains the same.

**Figure 5.**
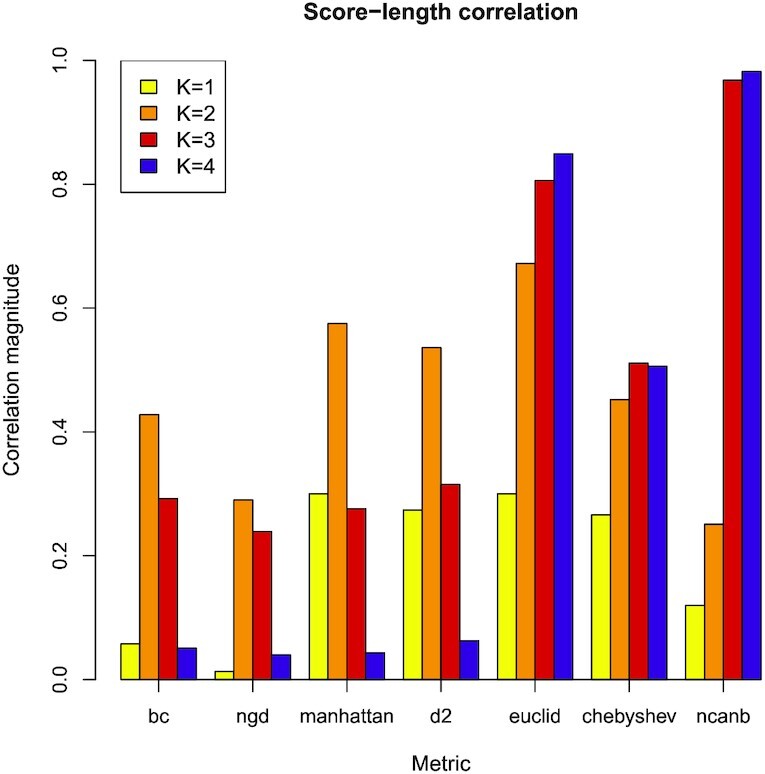
The correlation between protein sequence length and score for different metrics and *k*-mer lengths are summarised, for the ‘positives’ data set. The test was performed with Spearman’s rank correlation and the values of rho are given by the correlation magnitude (absolute values of rho). In each case, the *P*-value is less than 2.2e–16. These data are from the fly-worm system. Correlations of higher absolute magnitude tend to associate with weaker performance.

In Figure 5, we have calculated the correlations in a similar way to that described for Figure 4, but for a range of different *k*-mer lengths and scoring metrics. For the *D*2, BC, NGD and Manhattan metrics the highest correlation is given for *k*-mer length 2 (which corresponds to the minimum of the AUC curve in Figure 1), and the lowest correlation for *k*-mer length 4 (which gives the most accurate predictions for these four metrics). As mentioned previously, the Euclidian metric shows significant correlation for all *k*-mer lengths, with *k*-mer length 1 giving the lowest correlation magnitude, while the Canberra metric gives very high correlation for *k*-mers lengths 3 and 4. This metric’s accuracy is very poor (worse than random, see Figure 1) for these longer *k*-mers, although it is has low correlation and good performance for shorter *k*-mers.

Note that for *k*-mer length 4 amino-acids the number of possible *k*-mers is 20^4^ = 160 000. If two protein sequences of say 1000 amino-acids are compared then: (a) they are likely to have a very small number of possible 4-mers in common; (b) the number of 4-mers present in one sequence but not present in the other is likely to be higher; while (c) the vast majority of 4-mers will be absent in both sequences. The number of shared 4-mers will tend to increase with longer sequences and this may be basis of the observed correlation, although other factors may be involved. The selection of the distance metric used needs to be considered carefully in these circumstances because the usual assumption is that the majority of counts are non-zero. In these cases, where the majority of counts are zero, the Euclidian distance behaves poorly, the Manhattan distance behaves considerably better, and the Canberra distance only suffers from correlation and poor performance with the longer k-mers.

### Can the prediction of protein orthologs be improved by controlling sequence length?

**Figure 6.**
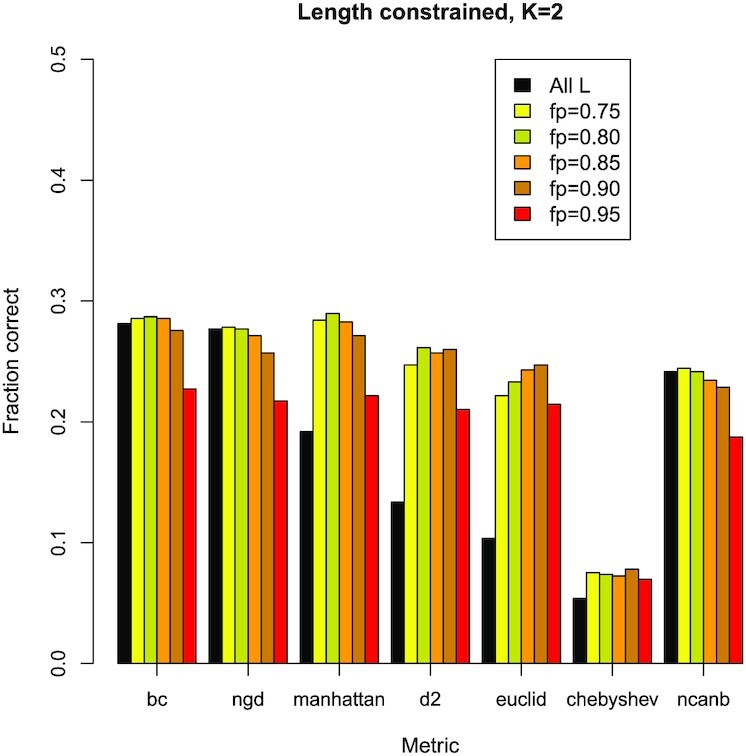
Controlling protein sequence length may lead to improvements of performance. The black bar gives the fraction correct with no applied length constraints, whilst the coloured bars give the fraction correct when the sequence length is constrained using the fp parameter in KAST, as given in the legend. For example fp=0.75 may be interpreted as the smallest of the sequence pair having at least 75% of the length of the longest: only sequence pairs that meet this condition are scored and ranked. The Manhattan, *D*2, and Euclidian metrics in particular show improved performance. These data are from the fly-worm system with k-mer length 2 amino acids.

The correlation between metric score and mean protein sequence length suggests that the predictive power of the metrics could be improved if sequence length was controlled in some way. To explore this, we added an option to KAST to constrain the length of compared sequence pairs so that the lengths are similar to within e.g. 80% of each other. Figure 6 shows the results when the sequence lengths are controlled in this manner, for a number of different metrics, and for *k*-mer length 2 (this *k*-mer has the highest absolute correlation according to Figure 5).

In Figure 6, the black blocks in the cluster of bars for each metric are the original predictive accuracy when length is not controlled. The degree of improvement or deterioration, when sequence length is controlled, can be seen for each metric by comparing the coloured blocks in a cluster to the black block. The figure shows little or no overall improvement for the NGD, BC and Canberra metrics, but the others, i.e. Manhattan, *D*2, Euclidian and Chebyshev metrics do show significant improvement.

The best options for the protein ortholog prediction problem in terms of accuracy are the BC and NGD metrics without any length filters (when these metrics are used with length filters we observed, at best, no significant improvement in accuracy). Manhattan is just as accurate as these two metrics for *k*-mer lengths 3 and 4 (and also for *k*-mer lengths 1 and 2, but only if the length filter is applied). The BC and NGD metrics operate in a similar manner. They consider sequence length to be an important component of sequence similarity. This can be a reasonable assumption for protein ortholog prediction, where sequences of protein orthologs are likely (but not always) to have similar lengths.

### Determining the likelihood of a prediction being correct for protein ortholog predictions

It is desirable for alignment-free search to be applied with a measure of the probability of success, which may be provided as an input parameter for the search. For example, in the context of protein ortholog prediction, by applying a particular cut-off score we should be able to limit search results to protein pairs with, for instance, at least an 80% chance of being a correct prediction.

First, consider that an optimal score threshold can be derived from the score frequency distribution plots shown in Figure 2. This score can be used as a filter or cut-off score: when applied, only scores less than this value are retained. Such a filter balances enrichment for true positives against coverage of the full data set, which mainly consists of true negatives. An advantage of applying this filter, therefore, is that it removes large numbers of negative sequence pairs from downstream analyses.

**Figure 7.**
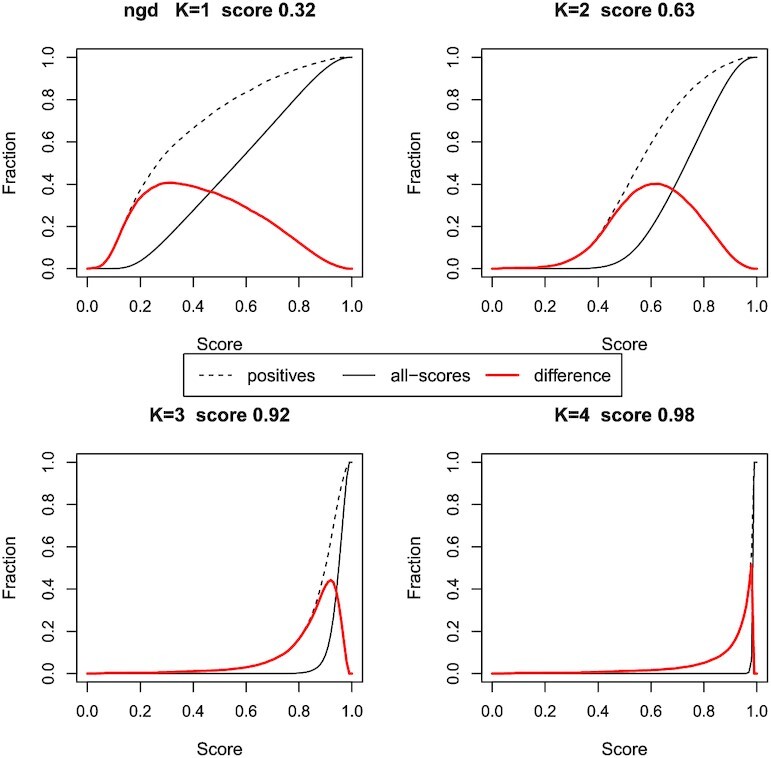
Selecting a cut-off score to optimise positive predictions. For protein sequence comparison, the ‘positives’ and ‘all-scores’ histograms are summed over the scores, from 0.0 to 1.0, to give the cumulative score frequencies. They are plotted as black lines (dots and solid, respectively). The ‘difference’ curve in red is the difference between these two curves. The peak of this curve suggests a score (given in the graph titles), such that the majority of true positive scores lie below this value, while true negatives tend to lie above it. The graphs for four different k-mer lengths are shown with the NGD metric. These data are from the fly-worm system.

A way of identifying this cut-off score is shown in Figure 7, where the plots show the cumulative score frequencies. These are calculated by summing the frequencies for each score from 0.0, up to the maximum score of 1.0, for the NGD metric. The cumulative frequency for the ‘positives’ distribution is subtracted from that for the ‘all-scores’ distribution to give a curve in red that shows the difference between these curves. The maximum of this curve indicates an optimal or cut-off score: applying this score as a filter increases the proportion of correct predictions, but it will yield less true positives over all. As *k*-mers get longer, the peak of the difference curve shown in Figure 7 gets sharper, indicating that the NGD metric is able to make a clearer distinction between true and false predictions. The sharpest peak is for *k*-mer length 4, and here almost all the positive scores (over 99%) are equal to, or below, the cut-off score of 0.98.

**Figure 8.**
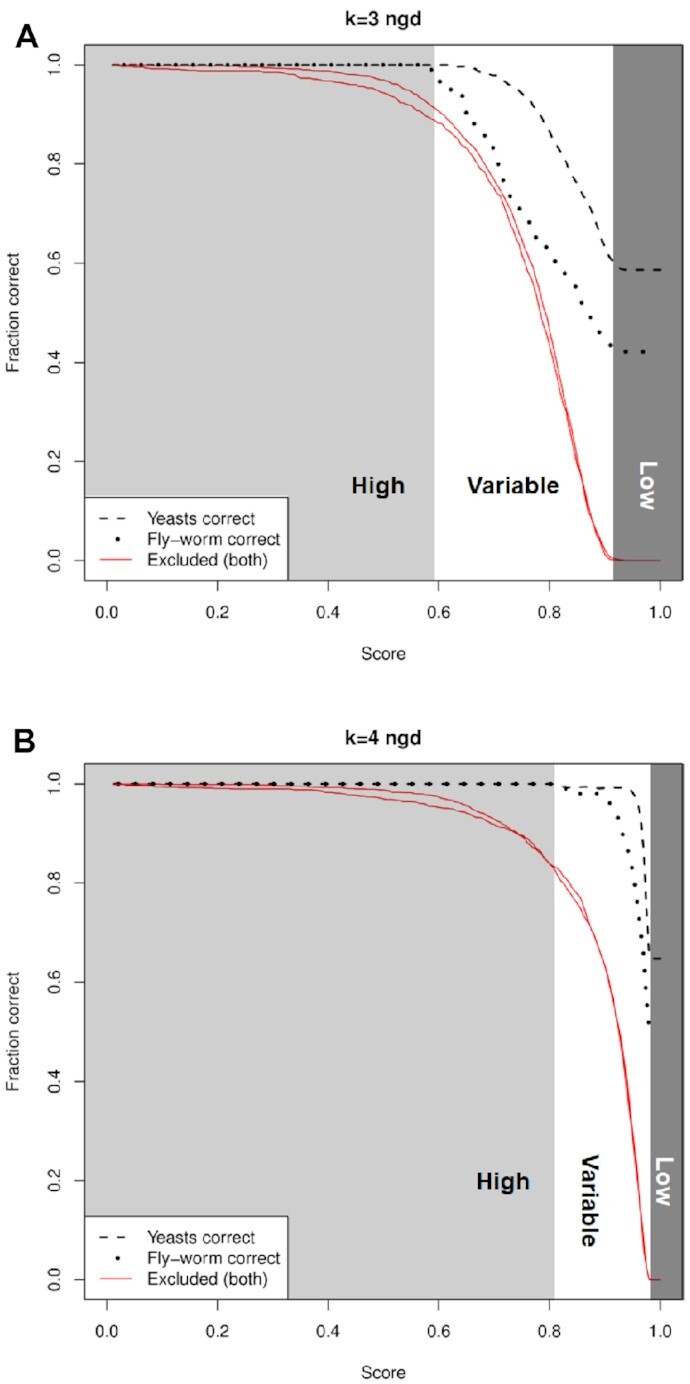
Mapping scores to fractions of correct predictions, to derive likelihoods or probabilities. Curves are shown for protein ortholog prediction for the NGD metric, with (**A**) *k*-mer length 3, and (**B**) *k*-mer length 4 amino acids, for the yeasts and fly-worm system. The black curves show the fraction of correct predictions when different cut-off scores are applied, while the red curves show how many correct predictions are excluded by applying the cut-off score. The difference between the black curves indicates the error that would be observed if we applied e.g. the yeast probabilities to scores from the fly-worm system. The area of the graph has been further divided into three areas corresponding to score ranges that correspond to high, variable or low accuracy predictions (based on the black curves).

In Figure 8, we expand on this idea. In order to obtain scores annotated with a measure of the probability of success, it is necessary to identify the proportion of correct predictions achieved at different score thresholds (i.e. cut-offs). The cost of applying a cut-off score filter is that less predictions are considered. This is because, by definition, applying the cut-off score will exclude protein pairs with scores above the cut-off score – but these scores will be less likely to be true positives than those below the cut-off score. For example, in the fly-worm system with *k*-mer length 4, without a cut-off score applied, all correct predictions are included (1081 out of 2147 predictions) and 0 are excluded. This corresponds to a predictive accuracy of 50% (based on the top hits). When applying a cut-off score filter of 0.915 (which is approximately at the most concave section of the red curve in Figure 7), there are 468 correct predictions with a predictive accuracy of over 95%. The cost of achieving this high accuracy rate, however, is that 613 of the possible correct predictions are removed by the filter. Figure 8 shows the proportion of correct predictions calculated for a range of cut-off scores: these are black curves in the graphs, and are shown for both the yeasts and the fly-worm system. The graphs also show the cost in terms of the correct predictions excluded by the filter: these are the red curves.

It is possible to map the scores generated by the NGD metric onto the proportion of correct predictions using the graphs in Figure 8. In this way, we can make estimates of the probability of a prediction being correct. In Figure 8, the area of the graphs have been divided into three indicative areas corresponding to scores that lead to high, variable or low accuracy predictions (derived from the black lines). For *k*-mer length 4, the low area would correspond to the scores above the cut-off score given in Figure 7, i.e. above 0.98. Given that almost all correct predictions have scores less than this value, predictions with scores above this value are unlikely to be true (i.e. they may be allocated a likelihood of 0.0). Conversely, scores below 0.8 could be considered the high likelihood region: predictions are highly likely to be true with an accuracy over 95%. In between these scores would be a medium or variable region. In this region, it is possible to map a score onto a probability value, thus estimating the likelihood of the ortholog prediction being correct.

Once the mapping between scores and probabilities has been derived, it may then be applied to ortholog predictions between different pairs of species – not just to the yeasts or fly-worm system. Some care, however, is required. In Figure 8 it can be seen that there are different levels of accuracy between the yeasts system and the fly-worm system. Based on the top hit predictions for *k*-mer length 4, their accuracy is 65% and 50%, respectively. These two systems are composed of species pairs that are relatively close and distant in evolutionary terms. Using just these species may limit the accuracy of the probability estimates. It is possible, however, that a number of different species pairs separated with a range of evolutionary distances could be used to generate a more complete library of probabilities. The library could be used to estimate likelihoods for pairs of species, depending on their evolutionary distance. The objective function plays an essential role in this process: it is used to determine the true orthologs between a pair of species. Additional pairs of species require additional objective functions and test data sets.

### K-mer length and DNA fragment length influence predictive accuracy for taxonomic prediction

In our DNA sequence comparison example, sequences deriving from two non-overlapping fragments of a bacterial genome can be recognised as similar. Alignment-based methods are unlikely to perform well in this application because the fragments come from the same genome. They therefore do not share any long blocks of evolutionary conserved sequences that are required for alignments.

DNA sequence varies in length much more than protein sequence. With DNA, the length of the reference and query sequences can vary considerably with respect to each other: one could be a 100 bp short read, the other a 10 000 000 bp chromosome. As with protein sequences, the length of the DNA sequences can have a significant influence on the alignment-free metric scores.

**Figure 9.**
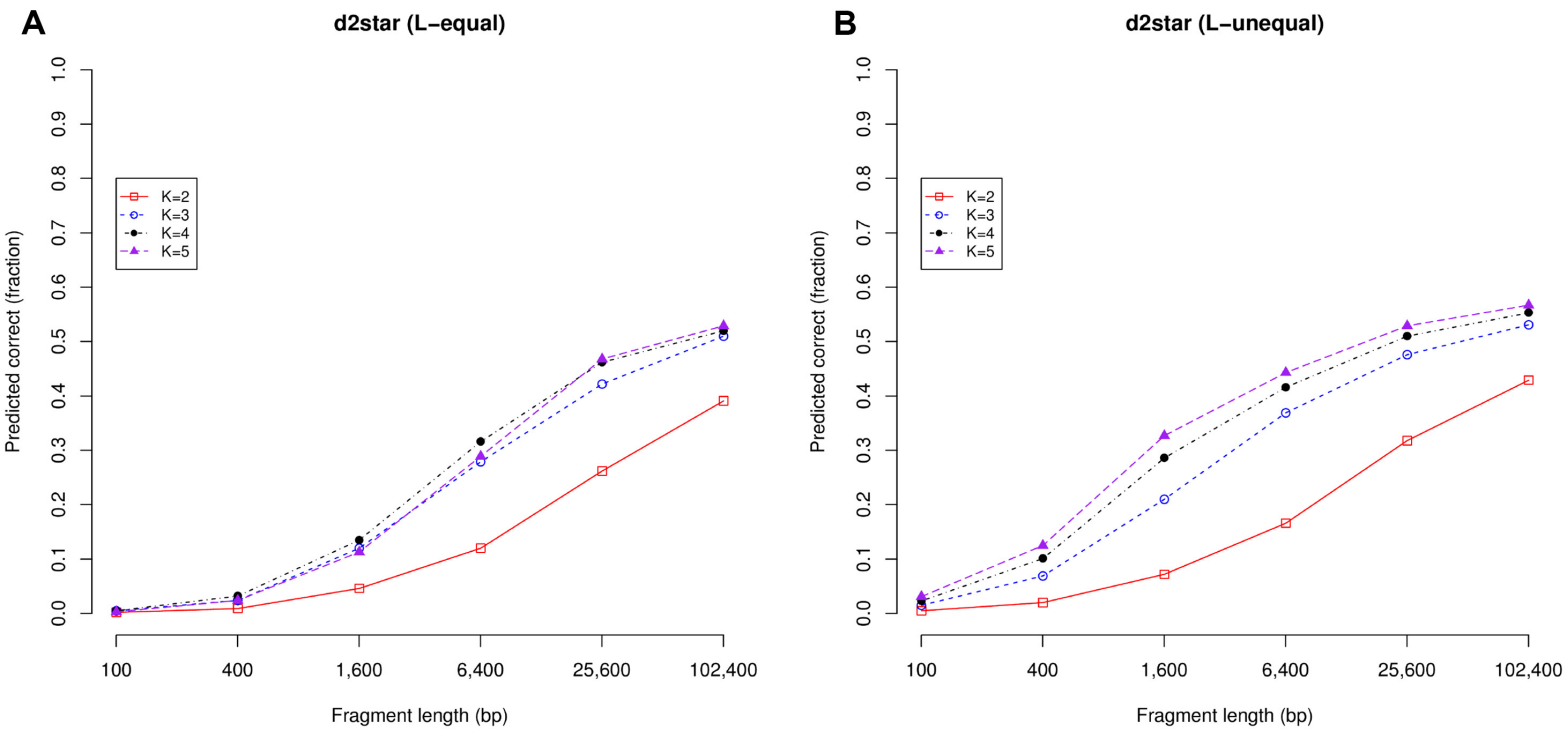
The metric used is d2Star, and the comparison has been performed on both (**A**) the L-equal case (reference and query lengths are equal and given in the x-axis); and (**B**) *L*-unequal case (reference is a 250 kb fragment, the query length is given in the x-axis). Longer DNA fragments lead to more accurate predictions than those made with shorter fragments. Increasing *k*-mer length from 2 to 5 bp improves predictive accuracy for *L*-unequal, and also for the *L*-equal case (but not consistently e.g. see *K* = 5). Here, the species is predicted and the maximum possible accuracy is 74%.

In Figure 9, we show taxonomic prediction results achieved whilst varying both the *k*-mer length and the fragment length. In the left-hand side graph we show the *L*-equal case, whilst the *L*-unequal is shown in the right-hand side graph. These results are for the d2Star metric, however the results are broadly similar for other metrics. The data for this figure, and equivalent data for other metrics, are given in Supplementary File 1.

Note that for the related problem of predicting the genera (rather the species) the predictive accuracy is significantly higher i.e. around 85% for *k* = 5 and 102.4 kb fragments). An issue with species prediction in these data sets is that only 493 of the 653 query sequences have a shared species classification with the reference data set, meaning that the maximum accuracy is 74%. This is higher for genera classification at nearly 99%.

Increasing the fragment length leads to greater predictive accuracy. Accuracy is very low for the shortest fragments, especially for *L*-equal: here it is 0.2% and 0.3% for *k* = 2 and *k* = 5 respectively with 100 bp. Accuracy increases with fragment length, tending to level off for the larger fragments (e.g. at 39.1% and 52.9% for *k* = 2 and *k* = 5 respectively with 102.4 kb and *L*-equal).

Increasing the *k*-mer length from 2 to 5 bp may improve predictive accuracy. For shorter sequence lengths, however, longer *k*-mers may lead to reduced accuracy. Indeed, Figure 9 shows that *k* = 5 is worse than *k* = 4 and no better than *k* = 3 for the smaller fragment lengths in the *L*-equal case.

There is a computational cost in using longer k-mers. The number of DNA words that need to be counted is equal to 4^*k*^ where *k* is the *k*-mer length, and four derives from the number of DNA bases. For example, a *k*-mer length of 3 bp results in 4^3^ = 64 words and a k-mer length of 5 bp results in 1024 words. Often a *k*-mer length of 4 bp is a reasonable choice (48). It has similar accuracy to a *k*-mer length of 5, but can be up to 4 times faster to calculate. The *L*-unequal case is also slower to compute than *L*-equal because it involves longer reference sequences. The ideal *k*-mer length is hard to specify in general terms as it depends on a variety of factors specific to the search problem. Some authors have observed that reducing the *k*-mer length can reduce resource requirements whilst maintaining a comparable accuracy (6); on the other hand, some authors observe that k-mer length may be marginal for good performance whilst noting that the appropriate choice of *k* is very much dependent on the alignment-free method and its parameters (15). Our results emphasis the relationship between sequence and k-mer length, with longer *k*-mers tending to requiring longer sequences for good performance. We elaborate on this below.

**Figure 10.**
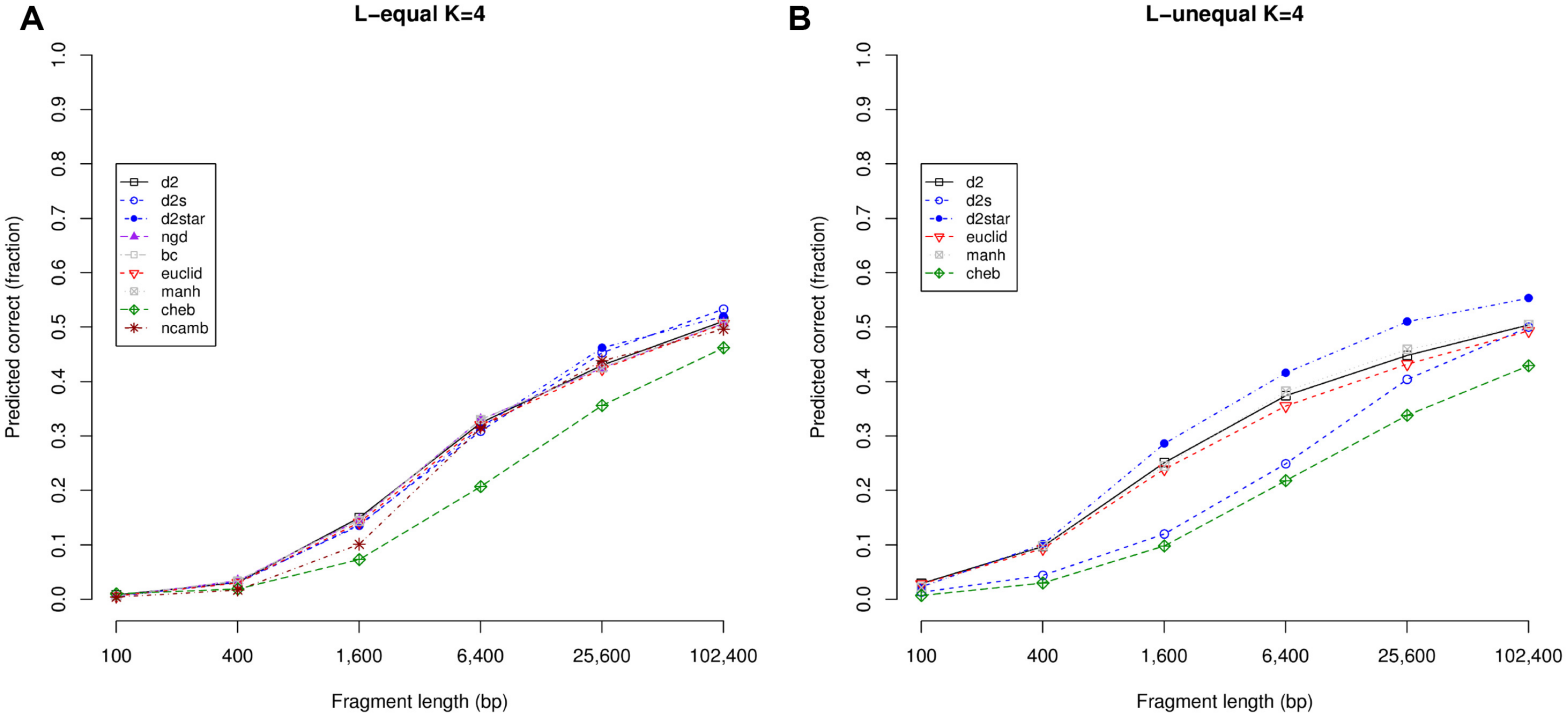
Predictions using different metrics. In (**A**) the *L*-equal situation, predictions with different metrics and DNA fragments of equal size give similar results, with Chebyshev giving the poorest performance. The situation is more complex in (**B**) the *L*-unequal situation: d2Star tends to outperform the others, with Chebyshev again giving the poorest performance. NGD, BC and Canberra are very poor if fragments are not the same length, hence they are not shown for the *L*-unequal situation.

Figure 10 explores the role of fragment length on the scores in the *L*-equal and *L*-unequal situations for *k*-mer length 4 bp. In the *L*-equal graph, results for nine different alignment-free metrics are shown. They tend to give similar performance, except for the Chebyshev metric that is noticeably worse for mid-length fragments, but just marginally worse for the shortest and largest fragments.

For the *L*-unequal situation in Figure 10, we show the results for 6 of the metrics. The NGD, BC and Canberra metrics perform very badly when the reference and query fragments are of different lengths, and so we have not included them here. These three metrics interpret the differing lengths in the *L*-unequal situation as highly dissimilar sequences, irrespective of *k*-mer frequencies, and hence they achieve effectively zero correct predictions. There is much more variability between the remaining metrics in the *L*-unequal situation. Chebyshev again gives the worse performance.

The methods that incorporate the background *k*-mer frequency via Markov approaches (here the background is calculated with single nucleotides) appear to perform differently to each other. Although they have similar performance in the *L*-equal case, in the *L*-unequal case, however, d2Star performs much better than d2S, but the difference is less for the shortest and longest fragments. The d2Star metric performed the best of all the metrics with the *L*-unequal data set, for *k*-mers of length 3–5 bp (see Supplementary File 1).

For the metrics that do not compute the background, there are some inconsistencies but they generally behave slightly better in the *L*-unequal case than in the *L*-equal case (due to its shorter reference sequences). The *D*2, Manhattan and Euclidian metrics are similar to each other in terms of predictive accuracy, with the Chebyshev method giving the weakest performance for these predictions.

### Score distributions vary with *k*-mer length and DNA fragment length

The distribution of score frequencies can vary significantly for key parameters used in alignment free sequence comparison. These include the *k*-mer length, the sequence fragment lengths, the relative lengths of the sequence pair being compared, and the scoring metric being deployed.

**Figure 11.**
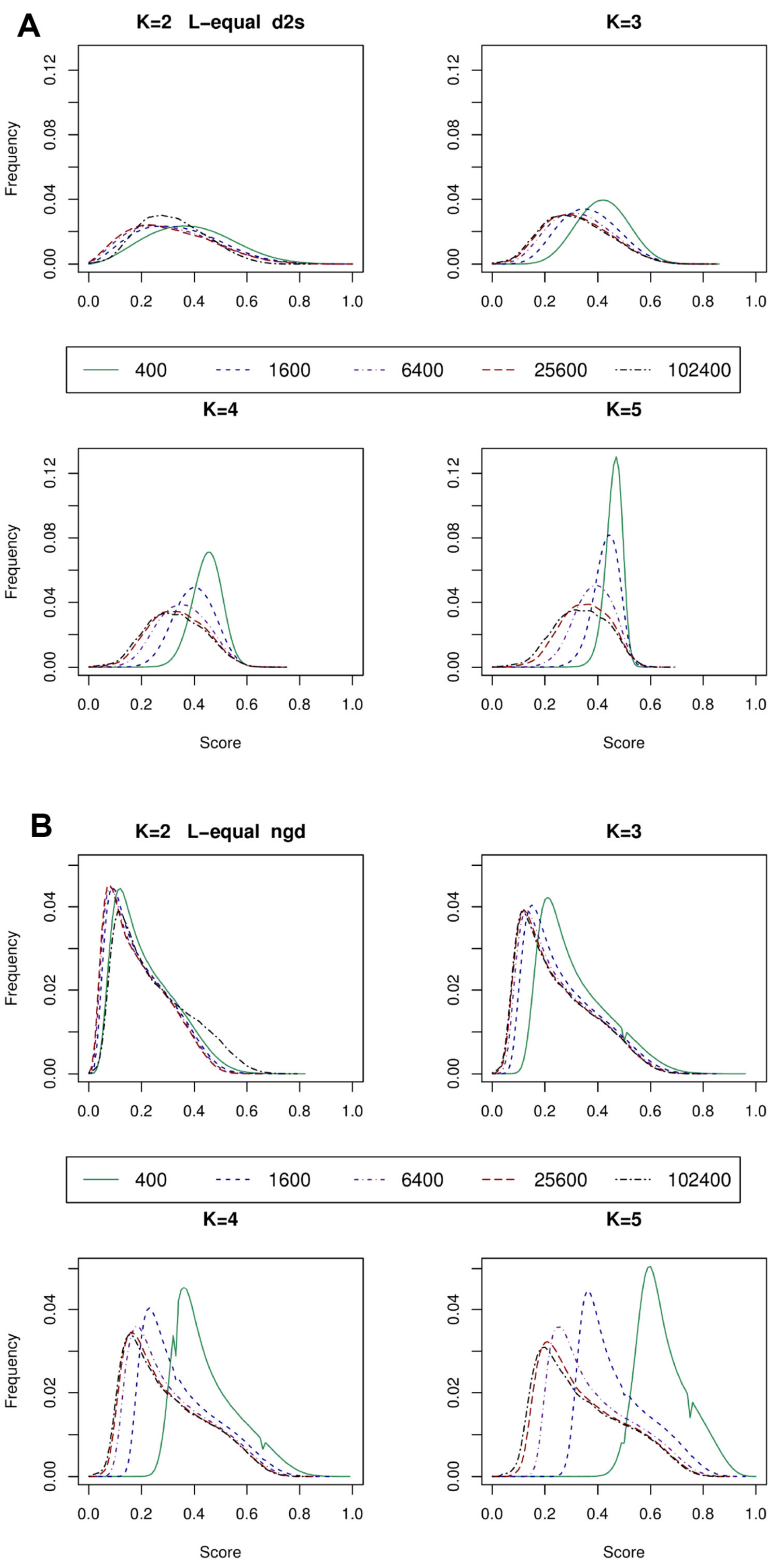
Frequency of occurrence (y-axis) of each score (x-axis) in the all-scores distribution. Results are given for two metrics: (**A**) d2S, and (**B**) NGD; with different DNA fragment sizes, and *k*-mer lengths. In both graphs the distribution is comprised from *L*-equal fragments, the sizes of which are given in the key. As the fragment sizes get longer, *k*-mer sampling improves, statistical noise is reduced, and the shapes of the distributions converge towards those of the longest fragments. For the shorter *k*-mers, the convergence occurs more quickly (i.e. with shorter fragments) than for the longer *k*-mers.

In Figure 11, we show score frequency histograms for the all-scores distribution (described in the methods), for the *L*-equal case for two metrics: d2S and NGD. For the shortest *k*-mer length, i.e. 2 bp, with both metrics the score distributions are relatively similar for all sequence fragment sizes. For the longest *k*-mer, i.e. 5 bp, the score distributions are much more dissimilar and distinct, except perhaps for the two longest fragments (*L* = 25.6 kb and *L* = 102.4 kb). These results highlight the differences between score distributions that arise from using different sequence lengths (and with the reference and query sequences equal in length). Figures with the distributions for other metrics, and for the *L*-unequal case, are given in Supplementary File 4.

Most analytical studies into metrics such as the *D*2 distribution have only considered cases where the pairs of sequences have equal or similar lengths. There is a risk that an erroneous assumption might arise, i.e. that alignment-free methods operate independently of sequence length. There is some theoretical reasons why this could be true. An influential review (49) interpreted DNA sequences in terms of chaotic dynamics using a visualisation of the DNA sequence called the Chaos Games Representation or CGR. The CGR is a visual representation of DNA sequences that generates very attractive images that have been shown to have fractal properties (50,51). They have been used as the basis for sequence comparison (52,53). Moreover, due to the link with fractals, the CGR highlights their scale independent nature – meaning that the frequency of occurrence of k-mers in a whole genome sequence is repeated throughout the genome, so that k-mer frequencies are much the same in short or long sequence fragments (54). This observation is true within limits: as sequences become shorter, however, there tends to be greater likelihood of divergence from the k-mer frequencies derived from the whole genome sequence. The divergence may be due to specific evolutionary or functional constraints being placed on the genetic code within shorter sequences, for example due to the need to encode a particular protein or, in bacteria, due to horizontal gene transfer. As sequences get longer, they contain more genetic features, and the pattern of *k*-mer frequencies then converges towards that observed in the whole genome. Sequence variability manifests as noise in the alignment-free metrics. Shorter sequences are more variable, with the result that scores from shorter sequence pairs are observed to have means with relatively dissimilar values (i.e. closer to 1.0 in the two metrics in Figure 11), as compared to the means generated from longer sequence pairs (which are closer to 0.0).

Once the *k*-mers have been well sampled via sequences that are sufficiently long, the score distributions tend to converge towards a similar shape. This can be more readily achieved with relatively short sequences for a k-mer length of 2 bp, which has just 16 *k*-mers to sample. Here a sequence length of 400 bp would allow each 2-mer to be sampled on average 400/16 = 25 times, thus allowing for accurate estimates of *k*-mer abundances. In contrast, a k-mer length of 5 bp has 1024 *k*-mers to sample, and with a sequence length of 400 bp more than half of these *k*-mers would have frequencies equal to zero: this poor sampling may adversely affect the performance of metrics. Much longer sequences are required to accurately estimate the 5-mer abundances, especially for the rarer *k*-mers.

In a whole genome sequence, as *k*-mers get longer, they are more likely to hold functional sequence, and they occur less frequently. *K*-mers of length 10 bp will occur once per million bases on average, i.e. just a few times in a typical bacterial genome. In contrast, k-mers in the 2 bp to 5 bp range, which we have focused on in this paper, are too short to code for more than 1 amino-acid, but are likely to occur 1000s of times in a bacterial genome. Their functional content is minimal: it is their frequency of occurrence that provides the important signal for the similarity metrics (48,55).

Longer *k*-mers, however, offer alternative strategies for detecting similarity. The presence or absence of long *k*-mers can give a reliable, binary signal that can be used for classification. This is because longer *k*-mers occur only rarely in sequences, if at all, and hence they may be used as biomarkers. This can be a very successful strategy and has been implemented in metagenomics software for taxonomic classification (56). For instance, the software Kraken performs classification tasks for metagenomics applications. It uses the presence of certain k-mers of length 31 bp to perform exact matches in sequence databases (57). It may also be worth mentioning that, if k-mers are sufficiently long, they could be considered to be alignments’, thus pushing at the limits of what could be called alignment-free sequence comparison. Nonetheless, two recent studies with a focus on bench-marking alignment-free algorithms have explored the more general use of *k*-mers of up to 10 bp and longer (14,15).

### Score distributions for different metrics, *k*-mers, fragment lengths and for both equal and unequal DNA fragment lengths

**Figure 12.**
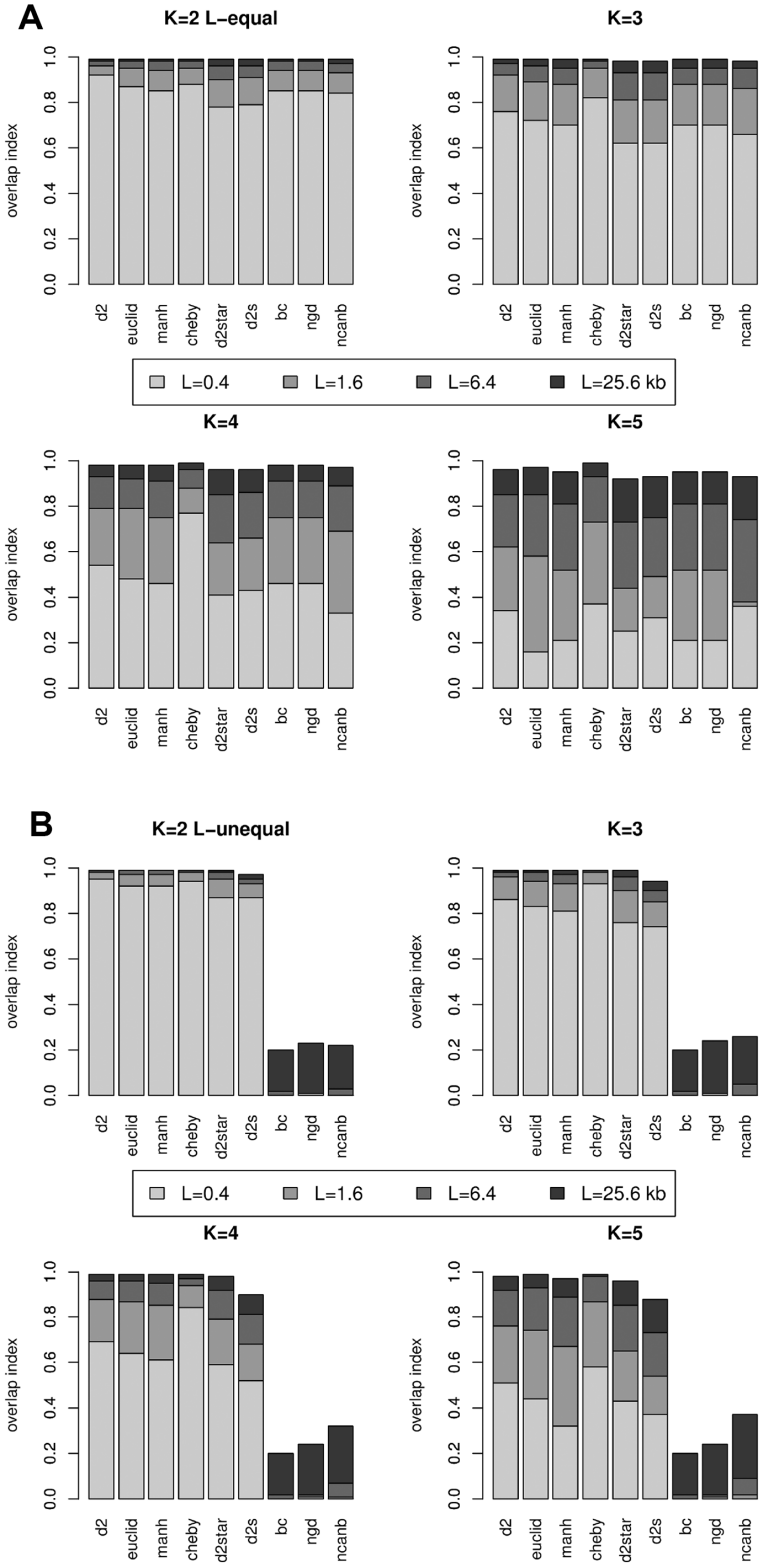
The overlapping index between the score distribution derived for the longest fragment (*L* = 102.4 kb), and the smaller fragment lengths given by the figure key, are shown. Results are given for four different *k*-mer lengths and for both the (**A**) *L*-equal and (**B**) *L*-unequal situations. The closer the overlap index is to 1.0, the better the *k*-mer sampling, leading to less noise (i.e. a stronger signal). Longer DNA fragments tend to provide better sampling of *k*-mers and more similar distributions, i.e. greater overlap. An exception occurs for the BC, NGD and Canberra metrics, which perform very badly in the *L*-unequal situation (when the reference and query sequences have dissimilar lengths).

Figure 12 summarises the results shown in Figure 11 for a range of different metrics. It highlights the ways in which the score distributions generated by alignment-free metrics depend on sequence length and k-mer length. In Figure 12, the similarity of different score distributions is compared by calculating the overlapping index between the distributions. To calculate the overlapping index, these distributions are compared to the score distribution involving the longest query sequence length, *L* = 102.4 kb. The overlap represents the convergence of the distributions, (see Figure 11). The overlapping index is shown for distributions generated using query sequence lengths from *L* = 0.4 kb up to *L* = 25.6 kb (given in the key). The reference sequences are given as for the *L*-equal and *L*-unequal cases (see methods for further detail). Results are given for four different *k*-mer lengths, 2, 3, 4 and 5. The closer the overlapping index between the score distributions is to 1.0, the more similar the distributions.

In Figure 12, the graphs for *k* = 4 (*L*-equal), shows that d2S (also shown in Figure 11) with the smallest length, *L* = 0.4 kb has a overlap index of 0.44. This means that the score distribution for *L* = 0.4 kb is not very similar to that for *L* = 102.4 kb: only a bit less than half of the distributions overlap. For *L* = 1.6 kb and *L* = 6.4 kb the overlap index values are 0.66 and 0.86, respectively. For the longest fragment shown in the figure key, *L* = 25.6 kb, the overlap index for d2S is ∼0.96 and this means that the score distribution for *L* = 25.6 kb has a relatively strong resemblance to that for *L* = 102.4 kb.

It is tempting to consider that, as the score distributions become more similar, the interpretation of their scores will also become more similar – for instance, in terms of the fraction of correct predictions as given in Figures 9 and 10. Unfortunately, this does not quite reflect the situation. Figure 10 shows that for d2S (*k* = 4 and *L*-equal) there is a significant increase in accuracy between *L* = 25.6 kb and *L* = 102.4 kb (from 45% to 53%), even though the overlap index between these score distributions is relatively high at 0.96. Other metrics show similar behaviour. The issue arises because true positives are most likely to be found in the tail of the distribution: relatively small changes in the tails can influence performance and the overlap index does not reflect the similarity between the tails well. It is also worth noting that these sequences differ in length by a factor of 4. Sequences that are more similar in length may still allow for a shared interpretation of the scores. Figures 9 and 10 show that as sequences get longer, the fraction of correct predictions tends to converge towards an upper limit. Thus, similarity in length may be more flexibly defined for longer sequences than for shorter sequences. In any case, it will depend on a number of parameters, including the k-mer length and metric used, and so we have not attempted to explore this precisely here.

In certain circumstances, it is advantageous to cut the sequence fragments so that they are all of equal lengths. Then the sequence pairs will generate scores from the same score-frequency distribution, with the same degree of statistical noise, and this can greatly simplify interpretation. This is particularly relevant for the query fragments – if the reference fragments are sufficiently long (e.g. a large chromosome fragment) they may not require any adjustment. The important point is that the sequence lengths influence the distributions of similarity scores for the alignment-free approaches we have explored in this section, with some metrics being more sensitive to this effect than others are. Although in most of our experiments the Chebyshev metric gives poor performance, it is worth noting that it does display the most consistent scoring with varying sequence length, as shown in Figure 12 (see the graphs for *K* = 3 and *K* = 4 for instance).

There is no need for the reference and query sequences to be of equal lengths, so long as the difference in length is consistent throughout the data set. A long reference sequence (*L*-unequal) will sample the k-mers relatively well, potentially compensating for the lesser sampling of the query sequence. In general, this gives more accurate results in our tests, and the overlaps given in Figure 12 are greater in the *L*-unequal case than the *L*-equal case. The overlaps for the BC, NGD and Canberra metrics, however, are very small. For these metrics, using the *L*-unequal reference sequence length (250 kb) with the much shorter query lengths gives very poor results. There is an assumption in the design of these metrics that sequences will be of similar length. It is therefore important to consider this when using these metrics because they do not function with sequence pairs of very dissimilar lengths.

### Determining the likelihood of a prediction being correct for DNA taxonomic classification

**Figure 13.**
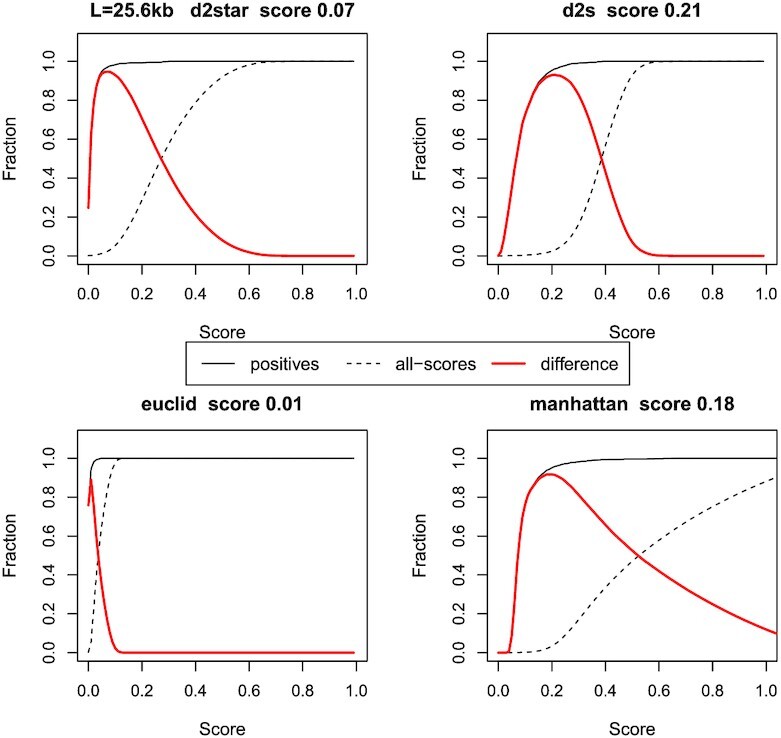
Selecting a score to optimise positive predictions. For DNA sequence comparison, the positive-only or ‘positives’ and the all-scores histograms are summed over the scores, from 0.0 to 1.0 (up to 2.0 for Manhattan), to calculate the cumulative score frequencies. They are plotted as black lines (solid and dots, respectively). The ‘difference’ line in red is the difference between these two curves. The peak of this curve suggests a score (given in the graph titles), such that the majority of true positive scores lie below this value, while true negatives tend to lie above it. The *k*-mer length has been set to 4 bp, the query length to 25.6 kb, and these results are based on the *L*-unequal data set. Four different metrics are shown.

Similarly to the protein ortholog prediction case, it can be advantageous to estimate the likelihood of a taxonomic prediction being correct. This can be performed in a similar manner to the protein case. Determining a cut-off score for DNA is more complex than for proteins, however, due to the greater variability of the sequence length and its influence on the scores. The curves in Figure 13 have been calculated in a similar way to those in Figure 7 but we have reduced the parameter set to only consider a *k*-mer length of 4 bp, a query length of 25.6 kb, and the L-unequal data set. Results are shown for four different metrics. In addition to the optimal scores, the figure indicates the different shaped distributions produced by different metrics.

**Figure 14.**
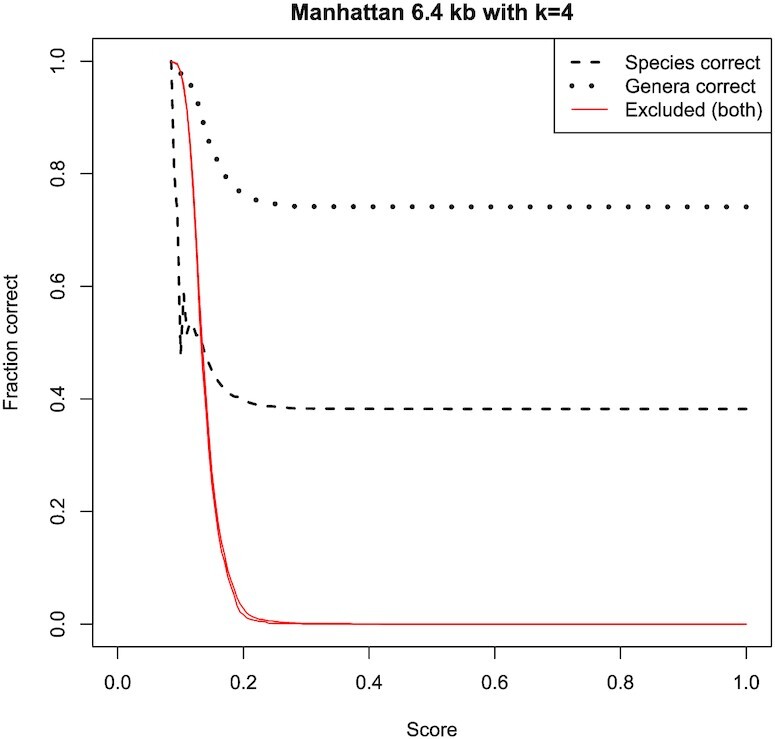
Mapping scores to fractions of correct predictions, to derive likelihoods or probabilities. The black curves show the fraction of correct predictions when different cut-off scores are applied, while the red curves show how many correct predictions are excluded by applying the cut-off score. Here DNA taxonomy prediction has been performed at two different levels (species and genus) with the Manhattan metric and k-mer lengths 4 bp. In each case, the scores are the same, but the objective function applied to determine true positives (i.e. the fraction correct) is different. Note two very similar red curves are plotted, corresponding to each taxonomic level.

Similar to Figures 8 and 14 shows the trade-off between applying a cut-off score to obtain more accurate taxonomic predictions, at the expensive of obtaining less predictions overall. The black curves show the fraction of correct predictions for different cut-off scores, whilst the red curves show how many correct predictions are excluded by applying increasingly stringent cut-off scores. The black curves may be used to map scores to probability values.

The importance of biological context is highlighted in Figure 14. There are two black curves because the similarity scores have been evaluated in two different contexts with two different objective functions i.e. predictions of either the species or the genus taxonomic level. The same set of scores is used in both cases, but the probability of a correct prediction, as represented by the black curves in the figure, is significantly different.

A recent software tool for phylogeny analysis (58,59) performs calibration using test data, with some similarity to what we have proposed here. Rather than explicitly including objective functions as we have done, however, in this application the objective function is implicitly embedded within the application. The objective is to predict the alignment-based identity score (percentage of identical nucleotides between two optimally aligned sequences). This is performed with self-supervised general linear models that use *k*-mer statistics derived from a variety of alignment-free methods. A strength of this method is that it combines multiple alignment-free approaches. However, given that it reproduces alignment-based statistics, it is not clear that it can address niches of interest where alignment-based methods perform poorly, such as the application we have presented here.

### Performance of the KAST software

As mentioned previously, we developed KAST because we were unaware of other alignment-free software packages that provided comparable functionality. In evaluating KAST, our analysis is therefore limited in terms of comparisons to other codes. Those that are available are restricted to DNA sequence.

Two other alignment-free codes that are designed for efficiency using C/C++, include CAFE (60) and ALF (61). CAFE is designed as a graphical application accessed via a GUI, although it can also be accessed via a command line interface. Like KAST, ALF is based on the SeqAn toolkit (43) but it was designed to look at a more limited set of distance measures than KAST. In addition, KAST offers additional functionality not present in CAFE and ALF, including parallel execution and scoring methods for amino-acid sequences.

For reference, we have provided some example KAST timings for protein comparison, using k-mers in the range of 1 to 4 amino-acids, the NGD metric, and outputting the scores for all comparisons (-n 0 option in KAST). These results are based on the two yeasts system *S. pombe* and *S. cerevisiae* (as the reference and query sequences in KAST, respectively), and comprises 34,491,394 protein sequence comparisons. The speed-ups available when using multiple cores are given in Table 3, and can be seen to be 7.0 times faster when 8 cores are used. There was some further increase when 16 cores were used but it was marginal. The lack of scaling here is likely related to issues described by other authors, with concerns including thread synchronization and Non-Uniform Memory Access (NUMA) (62,63). The use of *k*-mer length 4 amino acids can be particularly demanding on computational resources, both for time and RAM (running with 8-cores required 47 Gb of RAM).

**Table 3.** KAST speed-up when run on a x86_64 AMD system with 64 cores (2299 MHz) and 529 GB memory. The number of cores used are given in the top row. These results are based on the yeasts system (protein sequence). There was no advantage in running KAST with *K* = 1 and *K* = 2 beyond two cores: the speed-up remains much the same as for two cores

*K*-mer	16	8	4	2	1	Time
*K* = 1	–	–	–	×1.1	×1.0	2.56 (min)
*K* = 2	–	–	–	×2.2	×1.0	5.33 (min)
*K* = 3	×6.3	×6.0	×3.7	×1.8	×1.0	45.08 (min)
*K* = 4	×7.2	×7.0	×3.1	×1.7	×1.0	16.08 (h)

An example set of KAST parameters with 1 core and *k*-mer length 1 are: kast -c 1 -k 1 -q yeastSC.fa -r fissionYeast.fa -t ngd -s aa -n 0 -f blastlike -o out.kast.

We have compared execution times of KAST against ALF and CAFE, with the results shown in Table 4. For this comparison, we used the DNA sequences in the query data set with fragment length 25.6 kb. We performed an all versus all comparison of the sequences in this data set (3250 × 3250 comparisons) due to the more limited input options available with ALF and CAFE. Some adjustment of the input files was required because certain characters in the sequence files were not accepted, including the NCBI format of the headers (for CAFE), and in the DNA sequences ALF only accepts the characters ACGTN but not R. Moreover, CAFE requires each FASTA record to be in a separate file, which is not ideal when there are 1000s of sequences to process. Although these issues are relatively minor, they highlight the need for a robust and flexible implementation like KAST that is suitable for general use.

**Table 4.** KAST comparison to CAFE and ALF when run on a Intel(R) Xeon(R) CPU E5-2640 v3 at 2.60GHz with 32 cores and 64 Gb of RAM available. KAST was run with 1 and 16 cores (-c 1 and -c 16, respectively). CAFE has two modes, an initial run that does k-mer counting with jellyfish and writes files containing the counts that may then be used by subsequent runs. We have given times for the initial CAFE run. Both wall clock times (minutes:seconds) and RAM used (Gb) are given in pairs for each software package. Note that for most applications we would recommend using DNA *k*-mers in 3–5 bp range. The larger k-mers are shown here for reference purposes only

*K*-mer	KAST -c 1	KAST -c 16	ALF	CAFE
*K* = 3	0:07 0.2	0:09 0.2	0:09 0.3	10.20 1.0
*K* = 5	0:13 0.2	0:09 0.2	0:12 0.3	10:54 1.0
*K* = 7	1:43 0.4	0:18 0.4	1:27 0.4	21:42 1.0
*K* = 9	25:06 4.3	3:17 3.5	22:42 4.2	250:26 1.0

KAST is similar but slightly slower in execution speed when compared to ALF with 1 core, but just over 7 times faster when 16 cores are used. It is also has a slightly lower RAM requirement when used with multiple cores as compared to ALF. CAFE is more than 10 times slower than ALF, and more than 70 times slower than KAST when used with 16 cores. It does, however, use about 25% of the RAM of KAST and ALF.

Further optimisations of the KAST code are possible. We aimed to investigate alignment-free methods based on *k*-mer counts converted to frequencies. We therefore iterate over all possible *k*-mers. When using k-mers of 4 amino acids, 160 000 *k*-mers are possible, and the vast majority of these will have counts (and therefore frequencies) of zero. It would be possible to modify metrics to take advantage of this. It is also possible to take advantage of the strategies implemented in other codes (20,63,64).

## CONCLUSION

Many different metrics are described in the literature, and recent studies that bench-mark the metrics are an important step towards assessing their performance (14,15). These studies are very useful when deciding which alignment-free methods to use. Nonetheless, it is still difficult to understand the factors that lead to good performance, and there is still considerable uncertainty when tackling new application areas.

To complement the wide breadth of these studies, we have narrowed the focus and attempted to develop a more detailed understanding of a small number of more promising metrics. We have focused on two application areas, involving protein and DNA sequence, where we have large data sets that allow us to robustly characterise the similarity score distributions with different parameters and search scenarios. In particular, our DNA example is characteristic of an application where alignment-based approaches are likely to be ineffective. Here we attempt to recognise fragments from bacterial genomes based on their genome signatures: a recurring, fractal-like pattern of nucleotides that is used to encode disparate proteins (50,51,54). Two fragments from the same bacteria genome would not normally align against each other because they usually have no coding sequences in common – there are therefore no long continuous blocks of conserved sequence that can align. The frequency of usage of small *k*-mers, however, is still very similar in the two fragments, and to a lesser extent in other sequences that share the same taxonomic classifications (65). This provides a signal that alignment-free approaches are uniquely able to detect.

### Alignment-free methods appear to be simple—but the underlying biology is complex

One of the appealing features of alignment free methods is their speed and simplicity. Our investigation has highlighted that this simplicity arises because these methods remove biological context, with the result that a pair of sequences is reduced to a number, the similarity score. While the scores allow a number of sequences to be ranked by relative similarity, more sophisticated and objective interpretations of the scores require biological context to be restored in some form.

Moreover, our results have shown that different parameters, and different data sets, can lead to markedly different score distributions, and thus to different rankings of similarity. Even if the score distributions appear similar, small differences in the tail of the distribution can have a significant influence on performance. Additionally, different biological questions require different interpretations of those distributions: the concept of similarity as measured by different alignment-free methods does not readily map onto a researcher’s expectation of what similarity should measure.

The simplicity of alignment-free methods is therefore deceptive and it is important not to underestimate the challenge of interpreting the alignment-free scores in the context of the underlying biology, which is often complex, and further complicated by the subjective concerns of the researcher.

### Objective functions provide a flexible framework for the interpretation of alignment-free scores

We have proposed the role of an objective function in providing biological context that is required to evaluate the scores. Our objective functions involve mapping sequence identifiers to external knowledge bases in order to retrieve the missing biological context.

We have shown that a set of scores output from an alignment-free approach may be interpreted in different ways according to the objective function selected and the biological context it encodes. Flexible approaches are therefore important when interpreting similarity scores—alignment-free methods are based on a relative ranking, and there is no single objective measure that can be used to interpret this.

Although the concept of an objective function is key to interpreting and calibrating alignment-free analyses, we have not seen it discussed in a general manner in the alignment-free literature. Instead the presence of the objective function is usually implicitly assumed in applications developed with a single objective in mind.

We believe a greater consideration of the objective function’s role would help in understanding the practical application of alignment-free analysis. The advantage of explicitly incorporating the objective function is that it frees up software tools, allowing them to be more easily adapted to different search priorities, data sets, and applications. We have demonstrated that our approach is simple and flexible enough to work for both protein and DNA sequences

### Alignment-free scores may correlate significantly with the mean sequence length

In practice, for effective search strategies, our results show that an important consideration is the variability in the length of sequences. Whilst other authors have recently explored sequences that vary considerably in length (15), it is not clear if they have only investigated DNA sequence pairs of equal length, rather than the mismatched sequence lengths that naturally arise in many data sets, including those we have explored here (both DNA and protein). We have shown that alignment-free metrics may output scores that correlate significantly with the mean length of the sequence pair: we have not seen this reported before in the literature.

Different metrics have different sensitivity to length differences. Some metrics (NGD, BC and Canberra in some circumstances) consider sequence pairs that differ in length to be significantly dissimilar, not because of the content of those sequences, but simply because the sequences are of different lengths. While this is well known for the BC metric due to its established use in ecology (30), it is not so clear for the other metrics.

When predicting protein orthologs this sensitivity to relative sequence length may not be a problem, because reported orthologs often, but not always, have similar lengths: indeed, NGD and BC do very well in these cases. However, this behaviour is certainly problematic when considering the taxonomic classification of fragments of whole genome DNA, for instance from a genome or metagenome assembly, that may differ in length by orders of magnitude.

### Some alignment-free metrics give poor performance when most k-mer frequencies equal zero

We have attempted to focus on the concept of *k*-mer frequencies in alignment-free analysis applied to both DNA and protein sequence. Some care is required concerning how frequencies are implemented in practice. Strictly speaking, a frequency should be a continuous rather than a binary variable. Ideally, this means that each *k*-mer should have counts much greater than zero in each sequence—and this requires relatively short k-mers and relatively long sequences. While this is often achievable for DNA sequences, it is much more difficult with protein sequence due to its larger alphabet and shorter sequences.

Although a metric may be designed for *k*-mer frequencies, it is possible, through a combination of the k-mer length and sequence length parameters, to inadvertently create a situation where the vast majority of *k*-mers are absent, those that are present have very low counts, and the numbers of shared *k*-mers between two sequences could be minimal. In these cases, the *k*-mer frequencies would be represented as binary values of presence or absence, rather than as more continuous variables. This may not be obvious to the user and can result in unexpected behaviour that significantly impairs the performance of the metric.

The situation is likely related to what is known in machine learning as the ‘curse of high dimensionality’. With such high dimensional data, the concept of distance may be unclear because metrics behave differently to what seems intuitive based on 3D space (66). In such cases the concept of similarity may break down. Relating to our observations with protein sequences, other authors have shown that the Manhattan distance consistently performs better than the Euclidian distance in high dimensional data sets (67).

For example, with the longer k-mers we have used with protein sequences, the Canberra metric undergoes an unexpected drop in performance as the k-mer length increases, and the dimensionality of the space increases. While Canberra is a competitive metric for *k*-mer length 1, when *k*-mer frequencies are almost all above zero and most are shared between the sequence pair; it is at best random with k-mers of 3 or more amino acids, when few *k*-mers are shared between sequences, and those that are shared will rarely have counts above 1. We have shown that in this situation its true positive scores are highly correlated (negatively) with sequence length. The Euclidian and Chebyshev metrics also do poorly in these high-dimensional spaces, while some metrics do well (NGD, BC, and for the larger *k*-mers, Manhattan). Due to the smaller alphabet in DNA sequence, *k*-mers of length 4 or 5 bp do not usually suffer from these problems (unless perhaps the sequences are extremely short in length).

Some authors have classified methods that use the binary, presence or absence of k-mers differently from those based on frequency-based distances (6,68). Indeed, one could argue that sufficiently long and rare k-mers are essentially alignments, and as such, methods based on presence or absence of these *k*-mers should not be classified as alignment-free methods at all.

### Alignment-free scores can be annotated with probability values using objective functions and test data

We have calibrated alignment-free metrics using an empirical approach with objective functions and test data. This provides a relatively convenient way to generate score distributions corresponding to different parameter sets. In particular it allows us to isolate true positives and their score distributions. An important outcome is that these distributions may be used to calculate probability values for predictions made on unseen data sets with similar characteristics to the test data.

Calibrating alignment-free metrics in this way is highly advantageous for downstream processing. It provides a direct indication about how effective the search or sequence comparison procedure will be. However, this may not be straightforward: high quality test data can be time-consuming to curate, and developing an objective function to identify true positives may also require some time and technical skill. Nonetheless, without calibration there is a significant risk that the alignment-free metric and parameters chosen may be sub-optimal or even inappropriate for the purposes of the researcher.

### KAST software

Finally, due to the lack of alternatives, we have developed the KAST software as a general-purpose tool for fast and efficient alignment-free sequence comparison. This is freely available, along with various scripts, data sets and analyses that we have used in this paper (see the Data Availability section).

## DATA AVAILABILITY

An example set of Linux and Python scripts, including data and the KAST executable, that perform the main parts of our analysis, are available at https://pure.aber.ac.uk/portal/en/ (search with the title of this paper). There we have made available the scripts we used to make figures in this paper, including additional figures with different parameter sets to those shown in the main body of the paper. Alternatively, the DOI of the data set is: https://doi.org/10.20391/3e9573af-363b-4ff7-9f27-72eb84440b68.

The data we have made available for the protein use-case includes (a) FASTA files containing the protein sequences for the yeasts system and the fly-worm system; (b) FASTA files with the true orthologs presented as pairs of sequence for use with the KAST interleaved format; and (c) the associated DIOPT files with the ortholog mappings.

The data we have made available for the DNA use-case includes (a) FASTA files containing the DNA sequences for the strain (query) and species (reference) data sets; (b) FASTA files with positive pairs for use with the KAST interleaved format; and (c) the associated files with the NCBI taxonomic mappings.

For both these use-cases, scripts are available that run KAST, evaluate the output with an objective function, make score-frequency histograms, generate probability scores from the histograms, and annotate KAST output with the probability scores.

KAST source code is freely available via GitHub: https://github.com/martinjvickers/KAST. A manual is also provided: https://github.com/martinjvickers/KAST/wiki.

A docker container with KAST and a ‘lite’ version of the scripts, so-called because they are missing some of the larger data sets available from the DOI given above. This can be accessed at https://hub.docker.com/r/biocontainers/kast. Instructions for the commands to run this are found in the KAST documentation.

## Supplementary Material

lqac062_Supplemental_Files
