# Supplementary material for: Interpreting alignment-free sequence comparison: what makes a score a good score?
Source: NAR Genom Bioinform. 2022 Sep 5;4(3):lqac062. doi: 10.1093/nargab/lqac062 (PMC9442500; doi:10.1093/nargab/lqac062)
Supplement: lqac062_Supplemental_Files [file lqac062_supplemental_files.zip › Supp1_Tables.pdf]

Table 1.1: Fly-worm ortholog system, AUC values (area under the ROC curve).

kmer	bc	ngd	manhattan	d2	euclid	chebyshev	ncanberra
1	0.766896	0.753253	0.785874	0.782474	0.776481	0.741742	0.769072
2	0.767958	0.7708	0.743503	0.75298	0.717111	0.68559	0.740682
3	0.778448	0.793942	0.788795	0.766217	0.663646	0.654296	0.484025
4	0.822428	0.829394	0.830285	0.816185	0.645708	0.637457	0.463277

Table 1.2: Fly-worm ortholog system, fraction of correct top hits.

kmer	bc	ngd	manhattan	d2	euclid	chebyshev	ncanberra
1	0.167614	0.159091	0.112216	0.100852	0.102273	0.06392	0.159091
2	0.28125	0.276989	0.191761	0.133523	0.103693	0.053977	0.241477
3	0.401989	0.43892	0.423295	0.213068	0.045455	0.03267	0.038352
4	0.505682	0.517045	0.519886	0.389205	0.019886	0.02983	0.018466

Table 1.3: Yeasts ortholog system, AUC values (area under the ROC curve).

kmer	bc	ngd	manhattan	d2	euclid	chebyshev	ncanberra
1	0.913625	0.904487	0.881038	0.885402	0.874372	0.84006	0.910917
2	0.873028	0.895275	0.83277	0.834328	0.771726	0.738717	0.865777
3	0.897736	0.916062	0.910253	0.881037	0.700311	0.69258	0.510954
4	0.928359	0.935631	0.936228	0.921219	0.677861	0.66657	0.480374

Table 1.4: Yeasts ortholog system, fraction of correct top hits.

kmer	bc	ngd	manhattan	d2	euclid	chebyshev	ncanberra
1	0.248794	0.216299	0.159177	0.155623	0.156385	0.098502	0.229246
2	0.443006	0.428281	0.274689	0.198528	0.12719	0.055598	0.327748
3	0.542777	0.586443	0.570449	0.318609	0.033511	0.029449	0.025641
4	0.631886	0.647372	0.64264	0.542523	0.014721	0.023864	0.010914

Table 2.1: L-equal DNA fragment classification at species level, fraction of correct top hits for k-mers 2 to 5. Frag is the fragment length in kilobases.

KMER 2									
Frag	bc	chebyshe	d2	d2s	d2star	euclid	manhatta	ngd	ncanberra
0.1	0.01	0.008	0.008	0.002	0.002	0.007	0.007	0.01	0.006
0.4	0.019	0.02	0.018	0.01	0.009	0.017	0.019	0.019	0.017
1.6	0.067	0.063	0.065	0.044	0.046	0.064	0.066	0.067	0.062
6.4	0.174	0.168	0.172	0.103	0.12	0.171	0.173	0.174	0.172
25.6	0.311	0.287	0.315	0.238	0.262	0.316	0.31	0.311	0.32
102.4	0.43	0.404	0.429	0.376	0.391	0.43	0.43	0.43	0.424
KMER 3									
Frag	bc	chebyshe	d2	d2s	d2star	euclid	manhatta	ngd	ncanberra
0.1	0.007	0.006	0.006	0.005	0.005	0.006	0.007	0.007	0.005
0.4	0.028	0.02	0.027	0.025	0.024	0.026	0.028	0.028	0.022
1.6	0.125	0.092	0.128	0.112	0.12	0.126	0.123	0.125	0.116
6.4	0.284	0.224	0.287	0.274	0.279	0.283	0.283	0.284	0.288
25.6	0.406	0.364	0.409	0.418	0.422	0.405	0.405	0.406	0.404
102.4	0.487	0.45	0.488	0.502	0.51	0.485	0.487	0.487	0.474
KMER 4									
Frag	bc	chebyshe	d2	d2s	d2star	euclid	manhatta	ngd	ncanberra
0.1	0.006	0.01	0.008	0.006	0.005	0.006	0.006	0.006	0.004
0.4	0.034	0.019	0.031	0.03	0.032	0.03	0.032	0.034	0.017
1.6	0.146	0.073	0.15	0.137	0.135	0.141	0.143	0.146	0.101
6.4	0.33	0.207	0.324	0.309	0.316	0.32	0.33	0.33	0.315
25.6	0.425	0.356	0.43	0.453	0.462	0.423	0.425	0.425	0.438
102.4	0.507	0.462	0.511	0.533	0.52	0.506	0.506	0.507	0.496
KMER 5									
Frag	bc	chebyshe	d2	d2s	d2star	euclid	manhatta	ngd	ncanberra
0.1	0.006	0.005	0.006	0.004	0.003	0.004	0.006	0.006	0.013
0.4	0.028	0.019	0.026	0.03	0.024	0.022	0.025	0.028	0.018
1.6	0.134	0.059	0.133	0.121	0.113	0.121	0.133	0.134	0.077
6.4	0.327	0.176	0.318	0.287	0.289	0.319	0.326	0.327	0.289
25.6	0.441	0.331	0.43	0.461	0.468	0.429	0.441	0.441	0.437
102.4	0.524	0.449	0.517	0.539	0.529	0.515	0.524	0.524	0.514

Table 2.2: L-unequal DNA fragment classification at species level, fraction of correct top hits for k-mers 2 to 5. Frag is the fragment length in kilobases.

KMER 2									
Frag	bc	chebyshev	d2	d2s	d2star	euclid	manhattan	ngd	ncanberra
0.1	0	0.012	0.016	0.007	0.005	0.013	0.013	0	0
0.4	0	0.029	0.042	0.021	0.02	0.04	0.037	0	0
1.6	0	0.087	0.114	0.064	0.072	0.111	0.107	0	0
6.4	0	0.164	0.213	0.139	0.166	0.208	0.207	0	0
25.6	0	0.271	0.326	0.277	0.318	0.317	0.317	0	0
102.4	0	0.37	0.403	0.369	0.429	0.396	0.394	0	0
KMER 3									
Frag	bc	chebyshev	d2	d2s	d2star	euclid	manhattan	ngd	ncanberra
0.1	0	0.009	0.022	0.014	0.015	0.02	0.022	0	0
0.4	0	0.036	0.072	0.032	0.069	0.068	0.067	0	0
1.6	0	0.12	0.202	0.12	0.21	0.191	0.199	0	0
6.4	0	0.219	0.322	0.264	0.369	0.303	0.322	0	0
25.6	0	0.328	0.413	0.421	0.476	0.401	0.42	0	0
102.4	0	0.422	0.478	0.502	0.531	0.469	0.474	0	0
KMER 4									
Frag	bc	chebyshev	d2	d2s	d2star	euclid	manhattan	ngd	ncanberra
0.1	0	0.007	0.029	0.013	0.023	0.028	0.022	0	0
0.4	0	0.03	0.097	0.044	0.101	0.093	0.098	0	0
1.6	0	0.098	0.251	0.12	0.286	0.239	0.245	0	0
6.4	0	0.218	0.374	0.249	0.416	0.355	0.382	0	0
25.6	0	0.338	0.448	0.404	0.51	0.432	0.459	0	0
102.4	0	0.429	0.504	0.5	0.553	0.493	0.504	0	0
KMER 5									
Frag	bc	chebyshev	d2	d2s	d2star	euclid	manhattan	ngd	ncanberra
0.1	0	0.009	0.036	0.014	0.031	0.032	0.011	0	0
0.4	0	0.023	0.124	0.051	0.125	0.114	0.102	0	0
1.6	0	0.077	0.28	0.131	0.327	0.264	0.283	0	0
6.4	0	0.201	0.403	0.29	0.443	0.384	0.41	0	0
25.6	0	0.324	0.471	0.456	0.529	0.457	0.479	0	0
102.4	0	0.423	0.525	0.532	0.567	0.515	0.527	0	0
