# Supplementary material for: Interpreting alignment-free sequence comparison: what makes a score a good score?
Source: NAR Genom Bioinform. 2022 Sep 5;4(3):lqac062. doi: 10.1093/nargab/lqac062 (PMC9442500; doi:10.1093/nargab/lqac062)
Supplement: lqac062_Supplemental_Files [file lqac062_supplemental_files.zip › Supp2_Figure2extras.pdf]

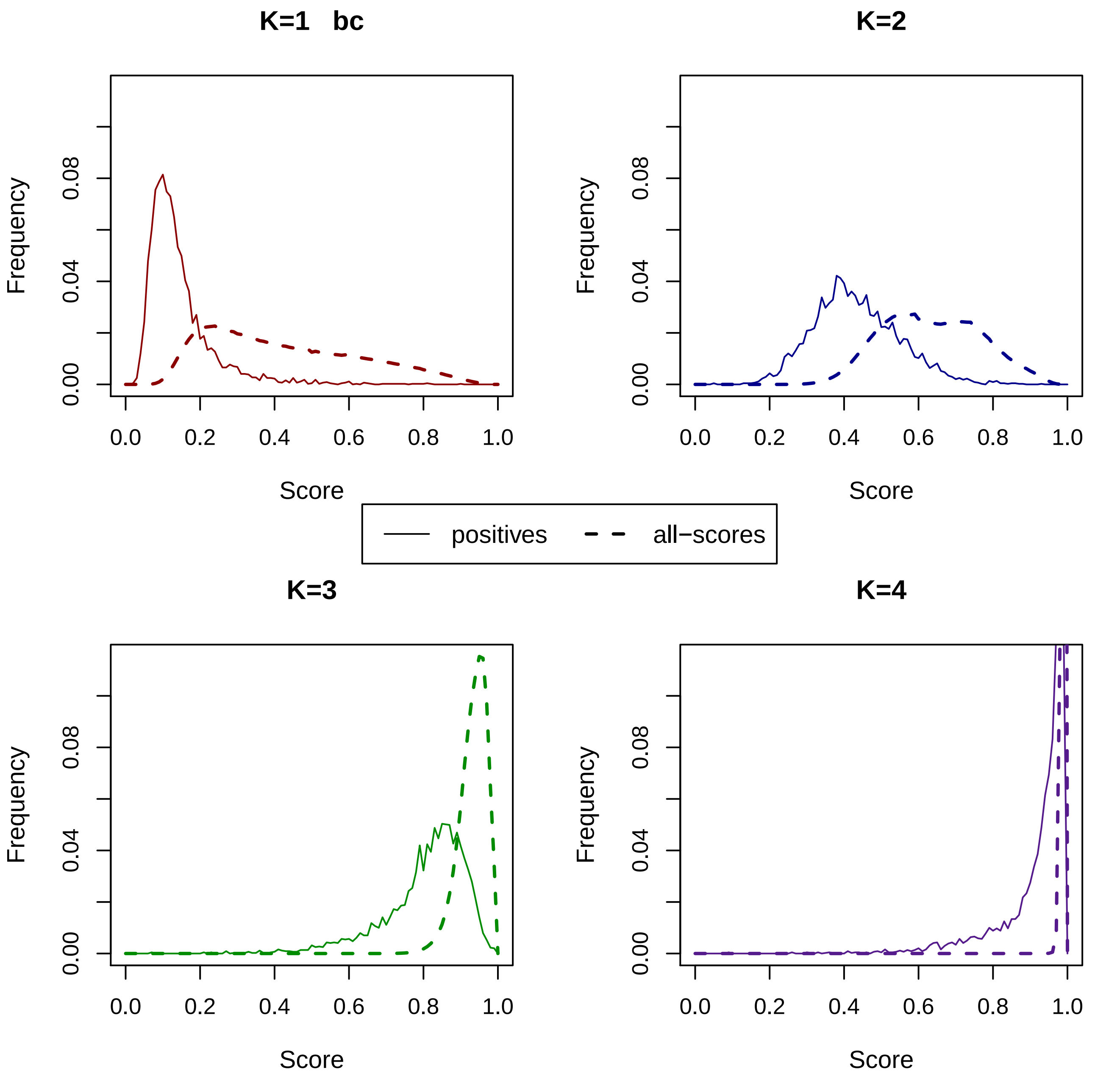
K=1 bc
K=2
K=3
K=4
Frequency
Score
0.00
0.04
0.08
0.0
0.2
0.4
0.6
0.8
1.0
positives
all−scores

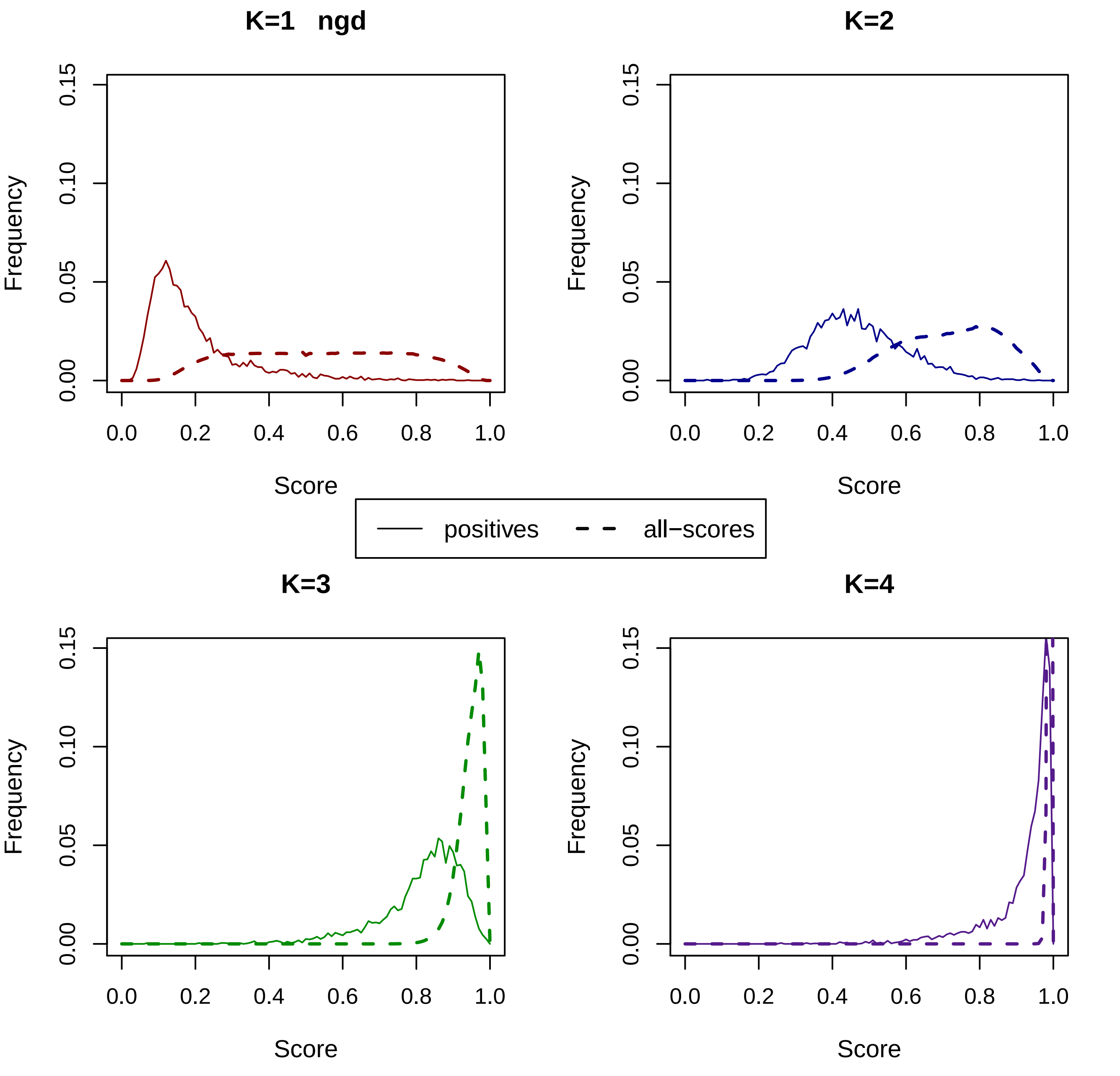
K=1 ngd
K=2
K=3
K=4
Frequency
Score
0.00
0.05
0.10
0.15
0.0
0.2
0.4
0.6
0.8
1.0
positives
all−scores

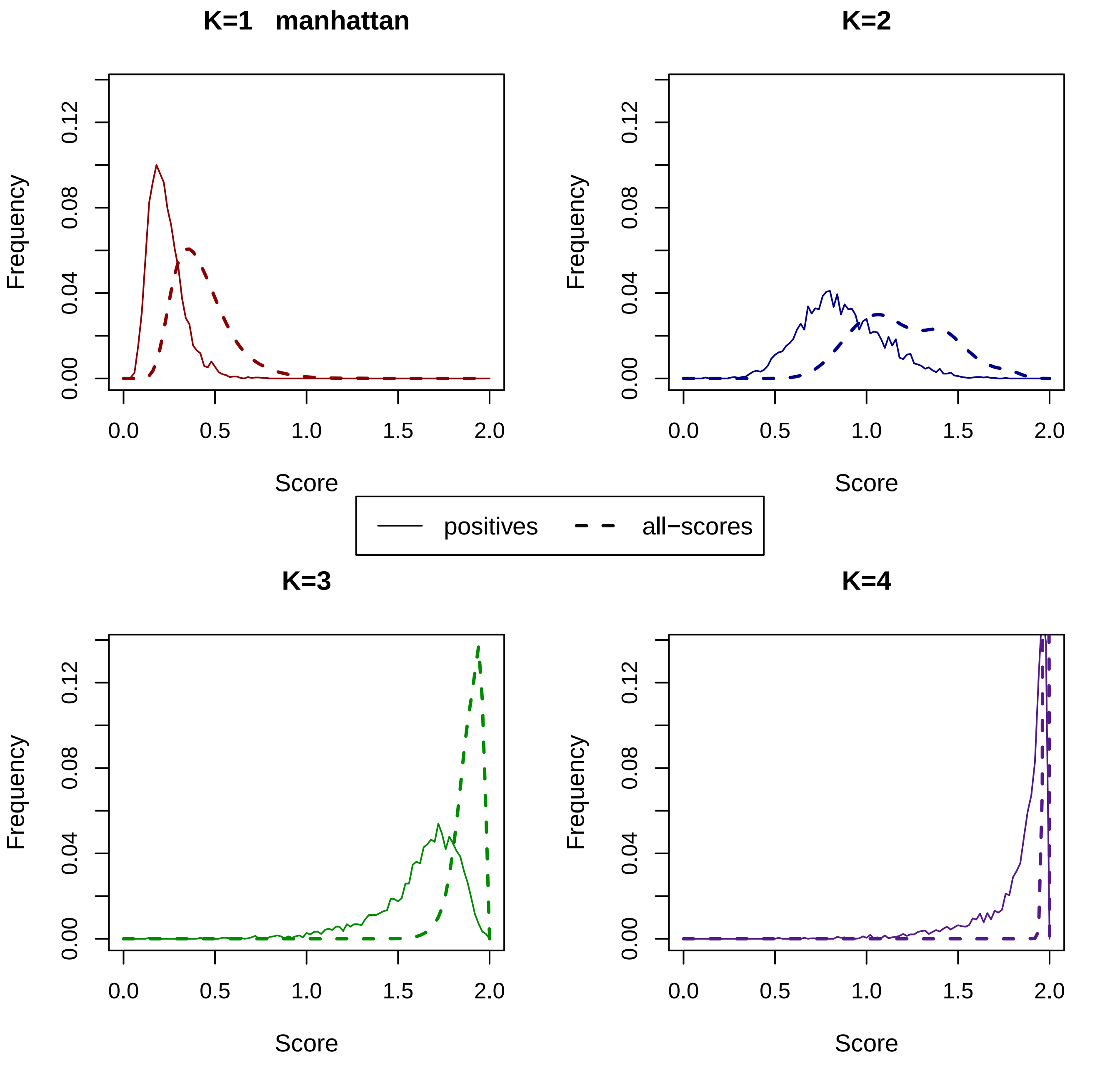

K=1 manhattan
K=2
K=3
K=4
Frequency
Score
0.00
0.04
0.08
0.12
0.0
0.5
1.0
1.5
2.0
positives
all−scores

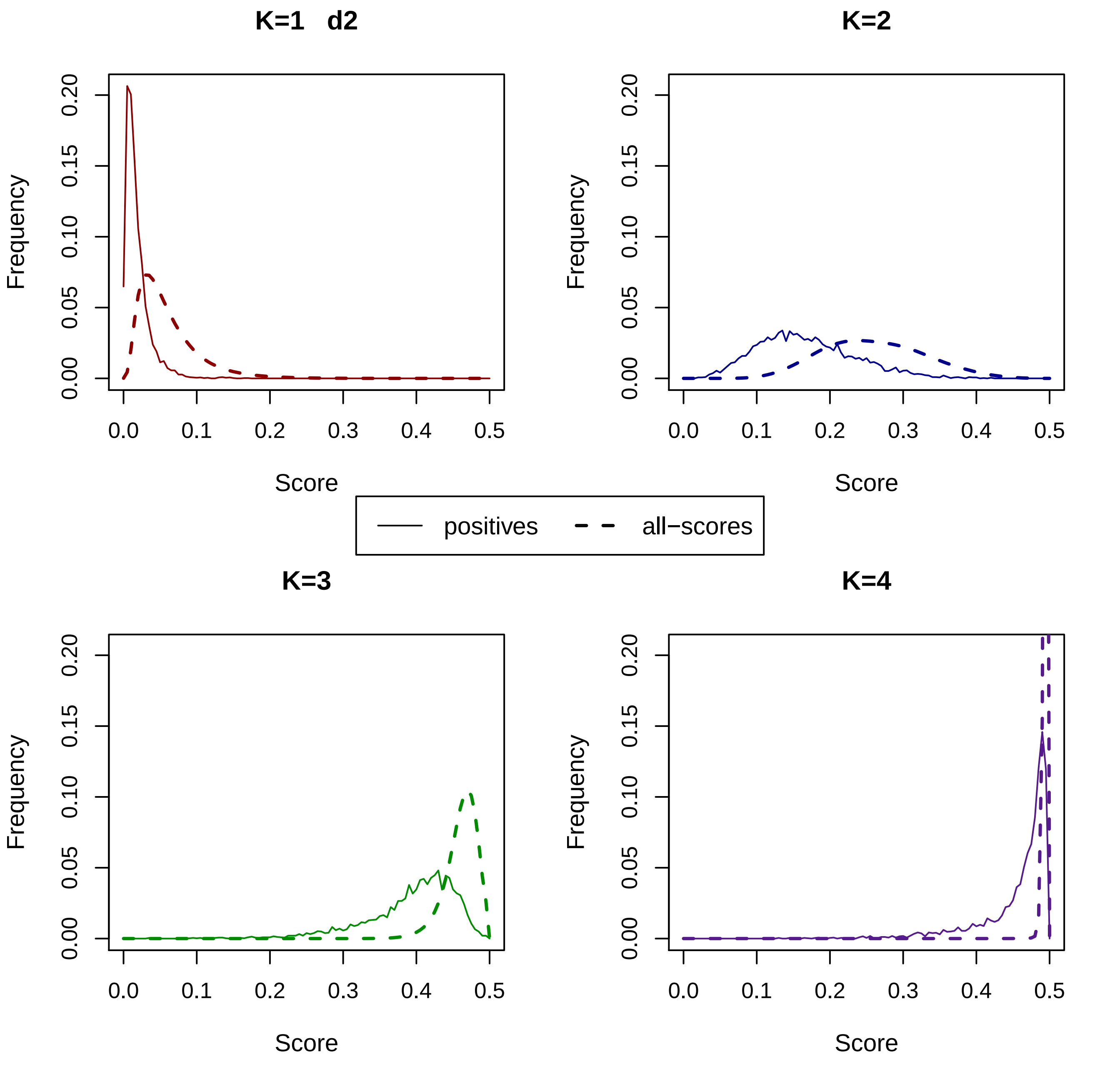
K=1 d2
K=2
K=3
K=4
Frequency
Score
0.00
0.05
0.10
0.15
0.20
0.0
0.1
0.2
0.3
0.4
0.5
positives
all−scores

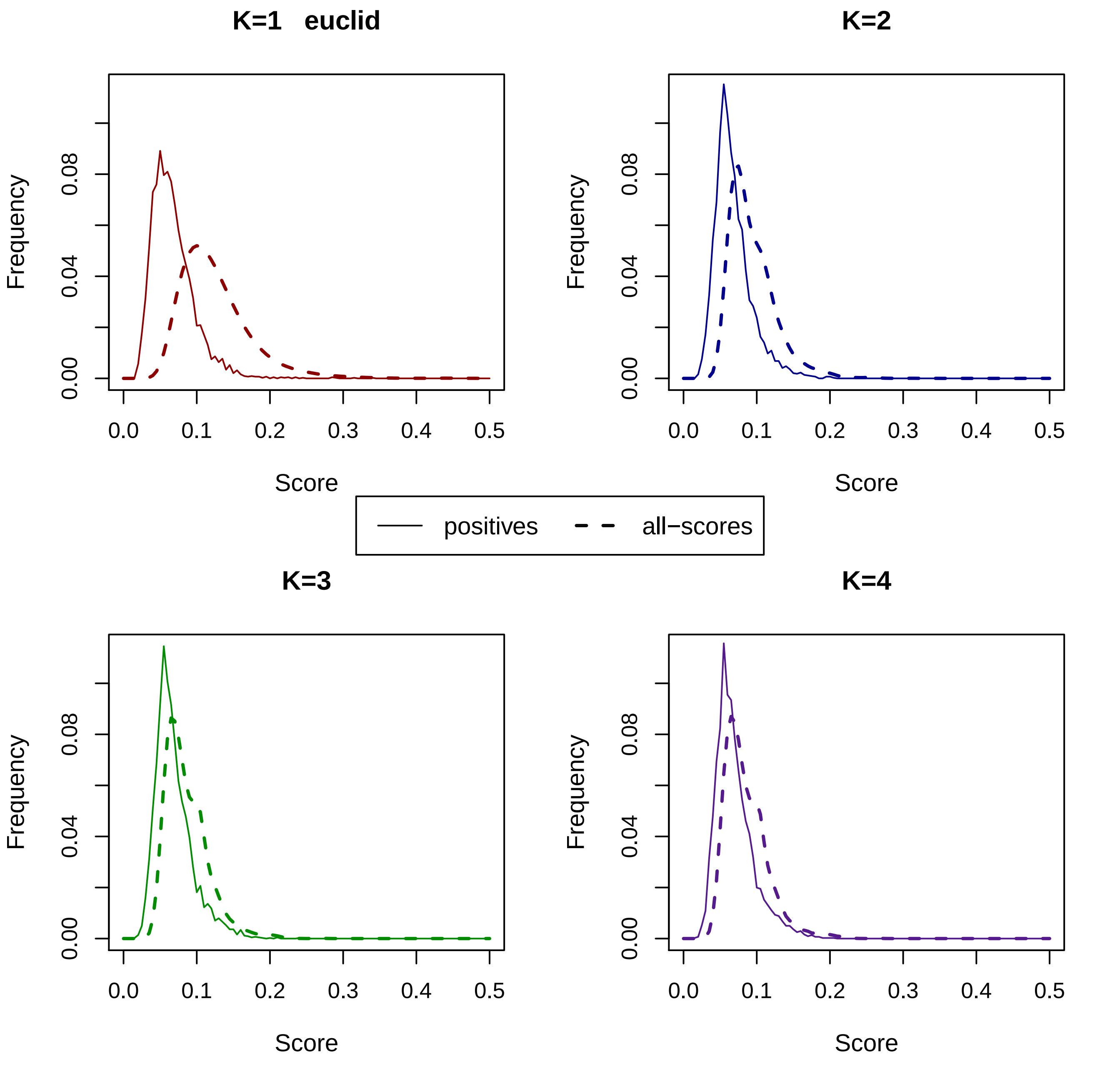

K=1 euclid
K=2
K=3
K=4
Frequency
Score
0.00
0.04
0.08
0.0
0.1
0.2
0.3
0.4
0.5
positives
all−scores

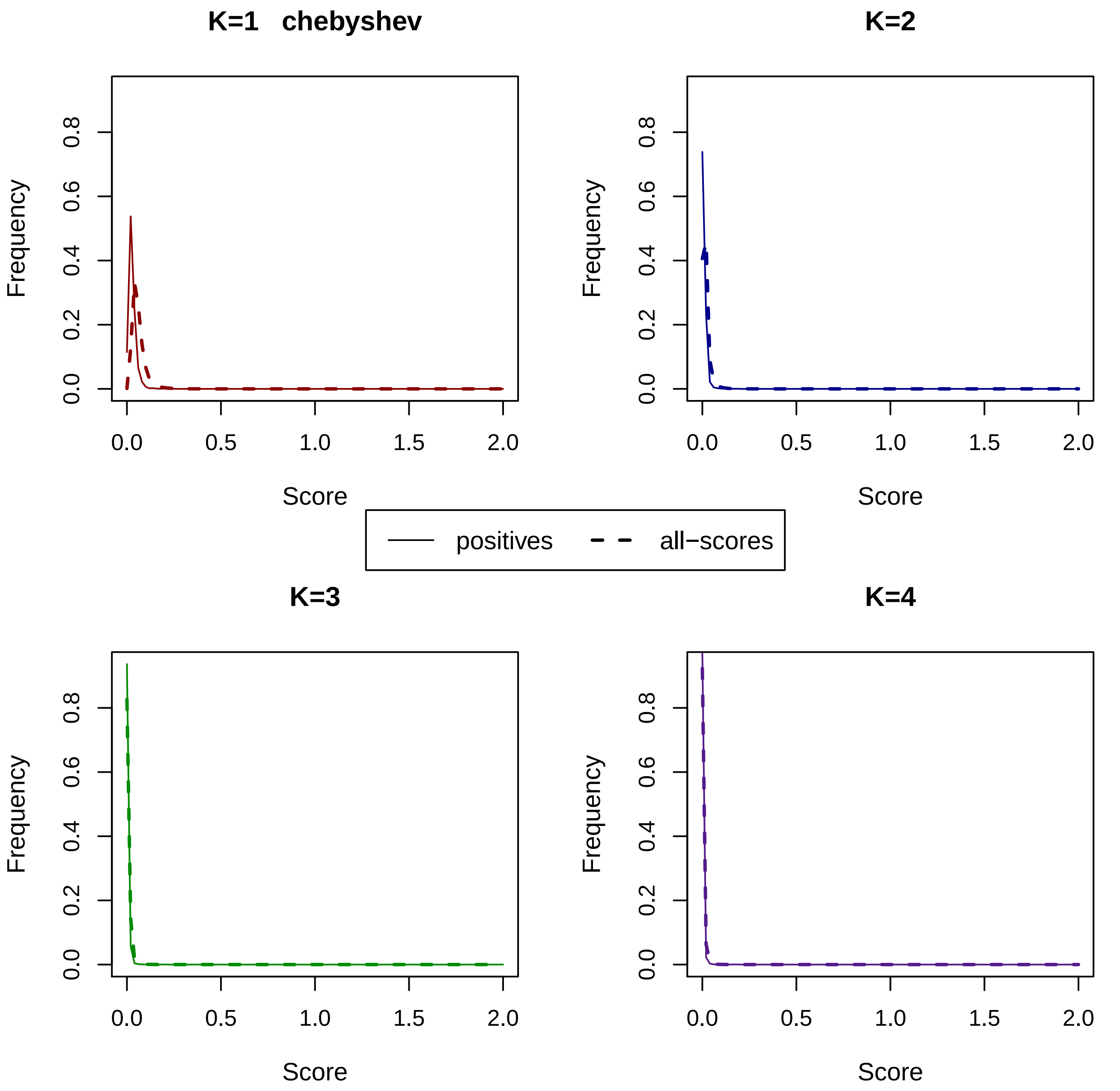
K=1 chebyshev
K=2
K=3
K=4
Frequency
Score
0.0
0.2
0.4
0.6
0.8
0.0
0.5
1.0
1.5
2.0
positives
all−scores

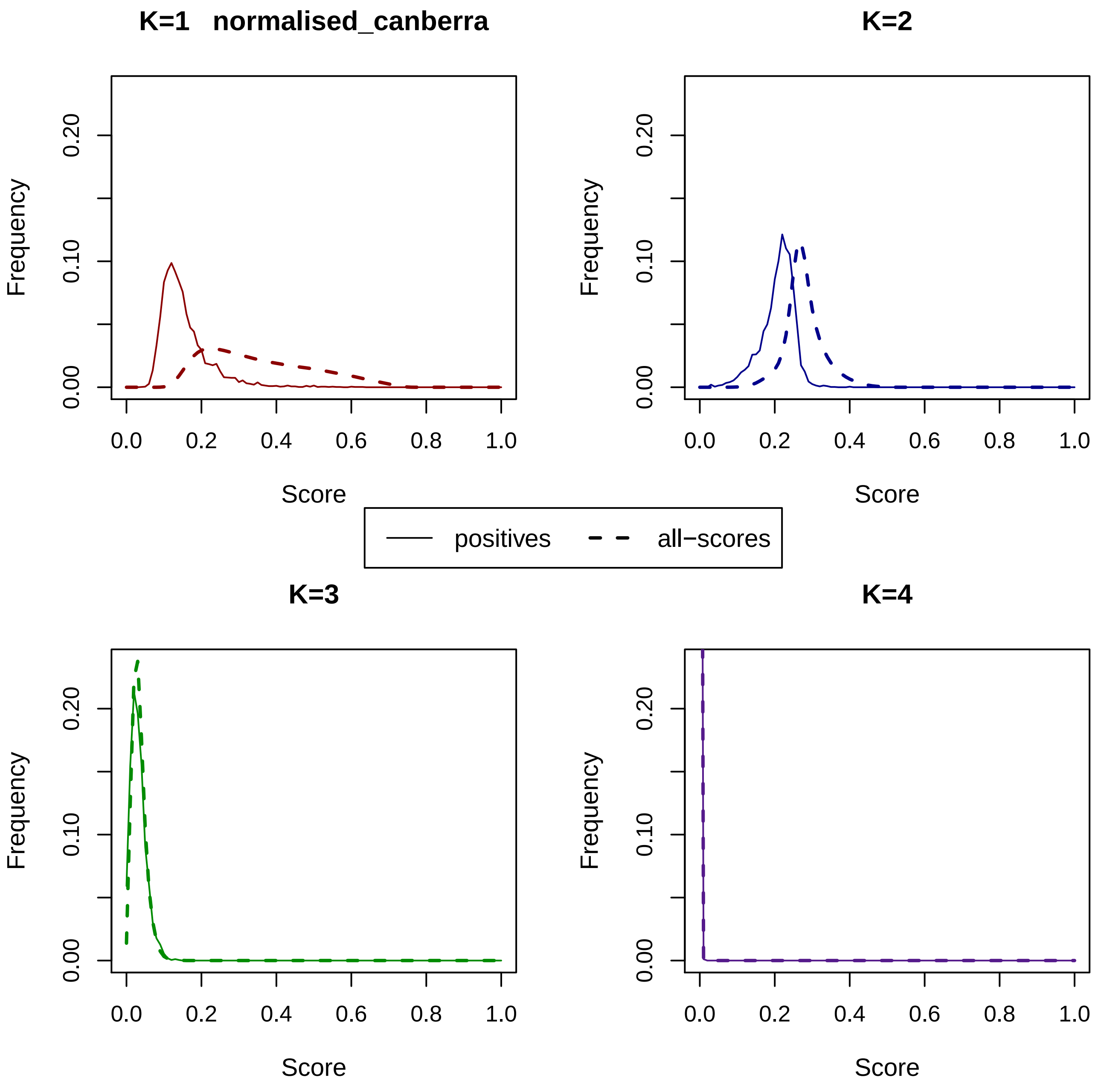

K=1 normalised_canberra
K=2
K=3
K=4
Frequency
0.00
0.10
0.20
Score
0.0
0.2
0.4
0.6
0.8
1.0
positives
all−scores
