# Supplementary material for: Interpreting alignment-free sequence comparison: what makes a score a good score?
Source: NAR Genom Bioinform. 2022 Sep 5;4(3):lqac062. doi: 10.1093/nargab/lqac062 (PMC9442500; doi:10.1093/nargab/lqac062)
Supplement: lqac062_Supplemental_Files [file lqac062_supplemental_files.zip › Supp3_Figure4extras.pdf]

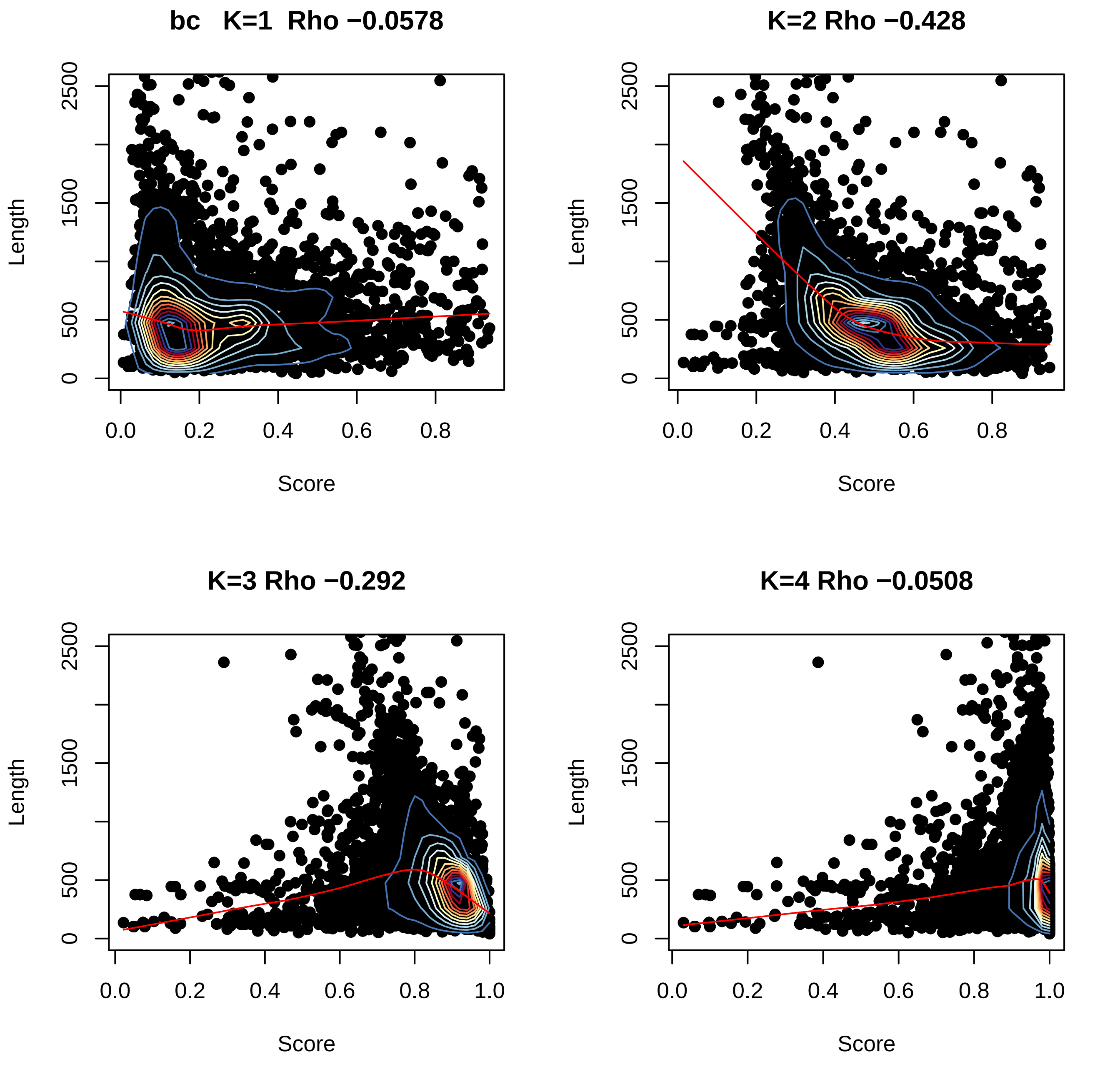
bc K=1 Rho −0.0578
K=2 Rho −0.428
K=3 Rho −0.292
K=4 Rho −0.0508
Length
Score
0
500
1500
2500
0.0
0.2
0.4
0.6
0.8
1.0

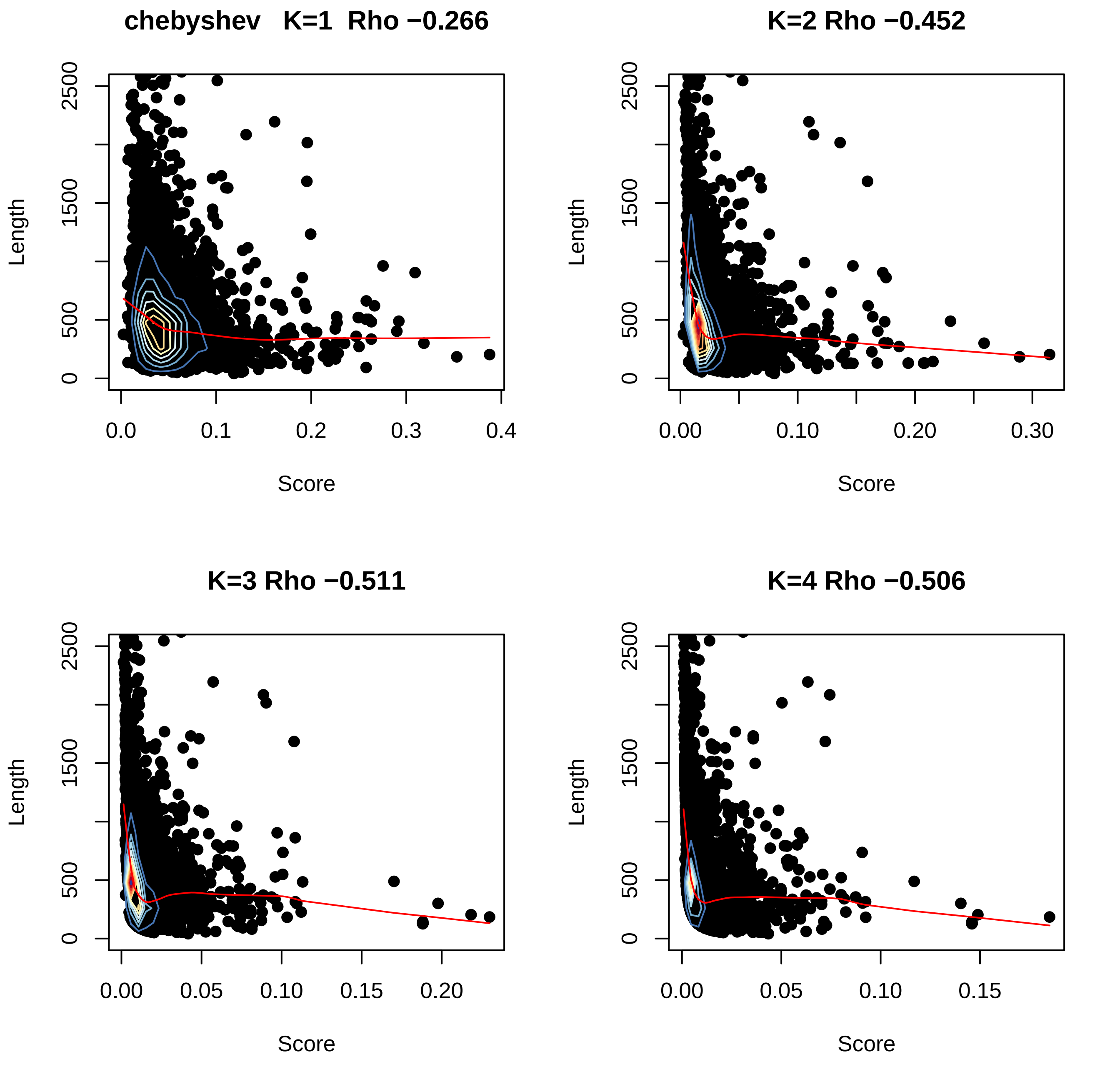
chebyshev K=1 Rho −0.266
K=2 Rho −0.452
K=3 Rho −0.511
K=4 Rho −0.506
Length
Score
0
500
1500
2500
0.0
0.1
0.2
0.3
0.4
0.00
0.10
0.20
0.30
0.05
0.15

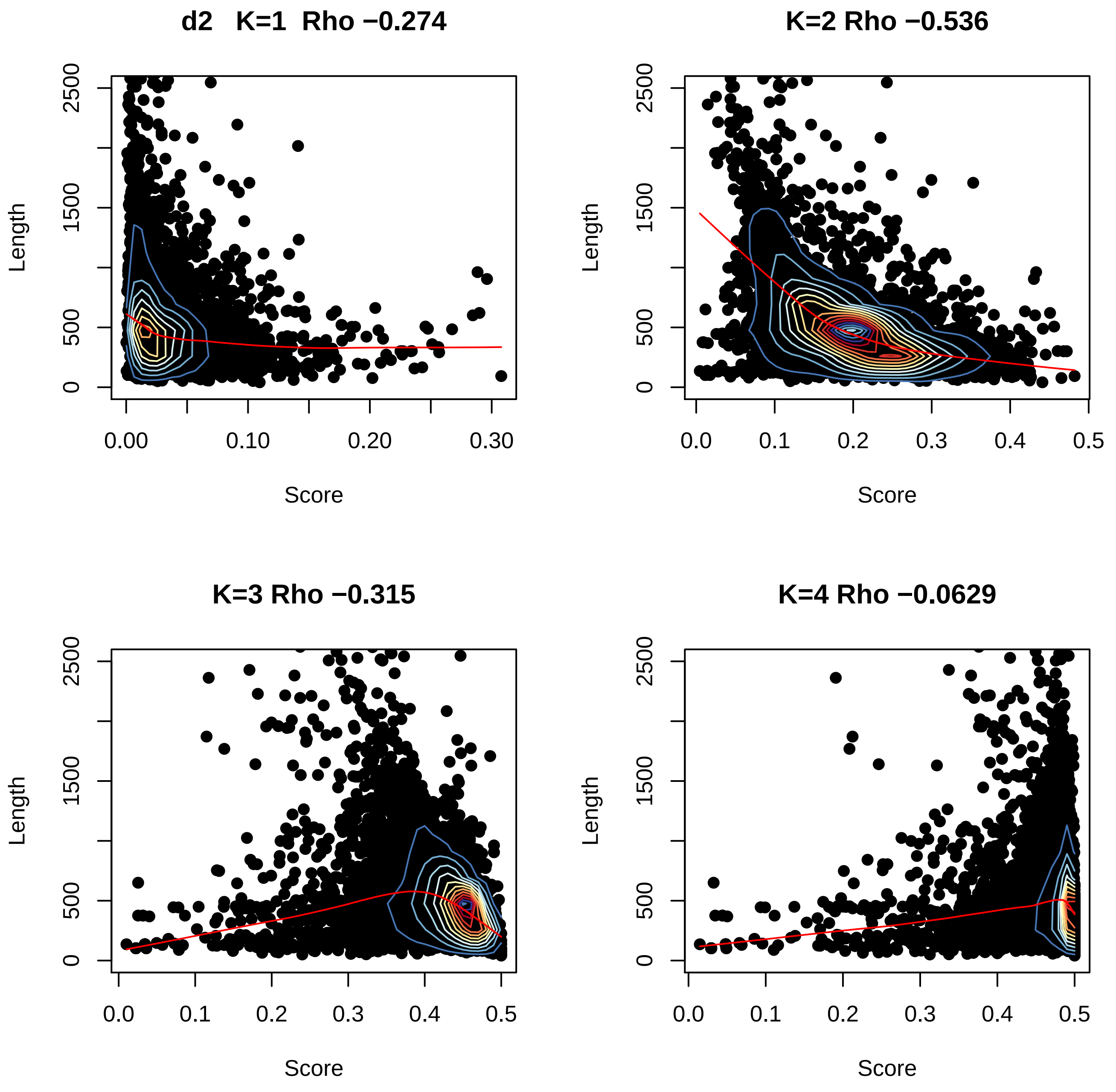

d2 K=1 Rho −0.274
K=2 Rho −0.536
K=3 Rho −0.315
K=4 Rho −0.0629
Length
Score
0
500
1500
2500
0.00
0.10
0.20
0.30
0.0
0.1
0.2
0.3
0.4
0.5

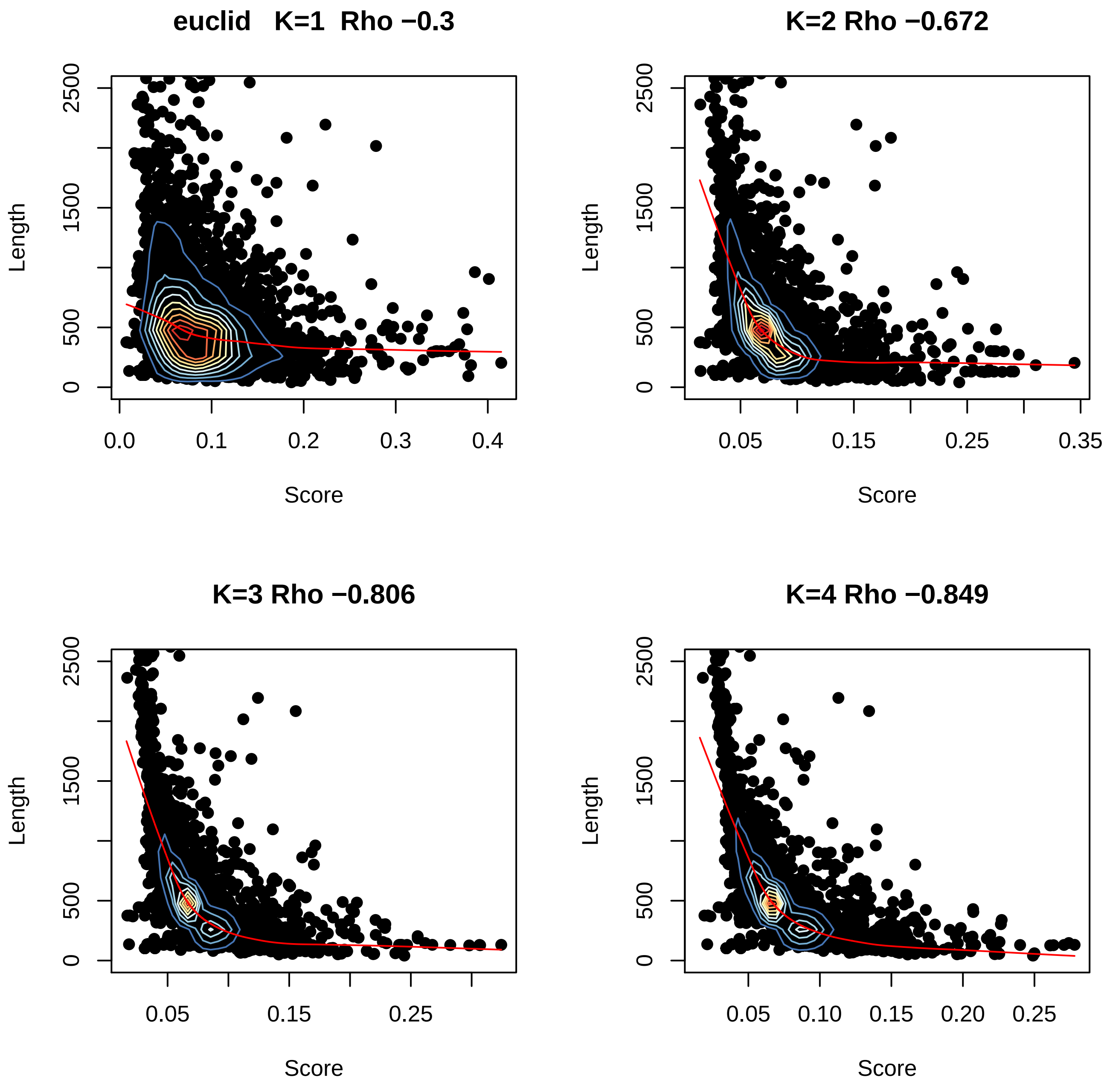
euclid K=1 Rho −0.3
K=2 Rho −0.672
K=3 Rho −0.806
K=4 Rho −0.849
Length
Score
0
500
1500
2500
0.0
0.1
0.2
0.3
0.4
0.05
0.15
0.25
0.35
0.10
0.20

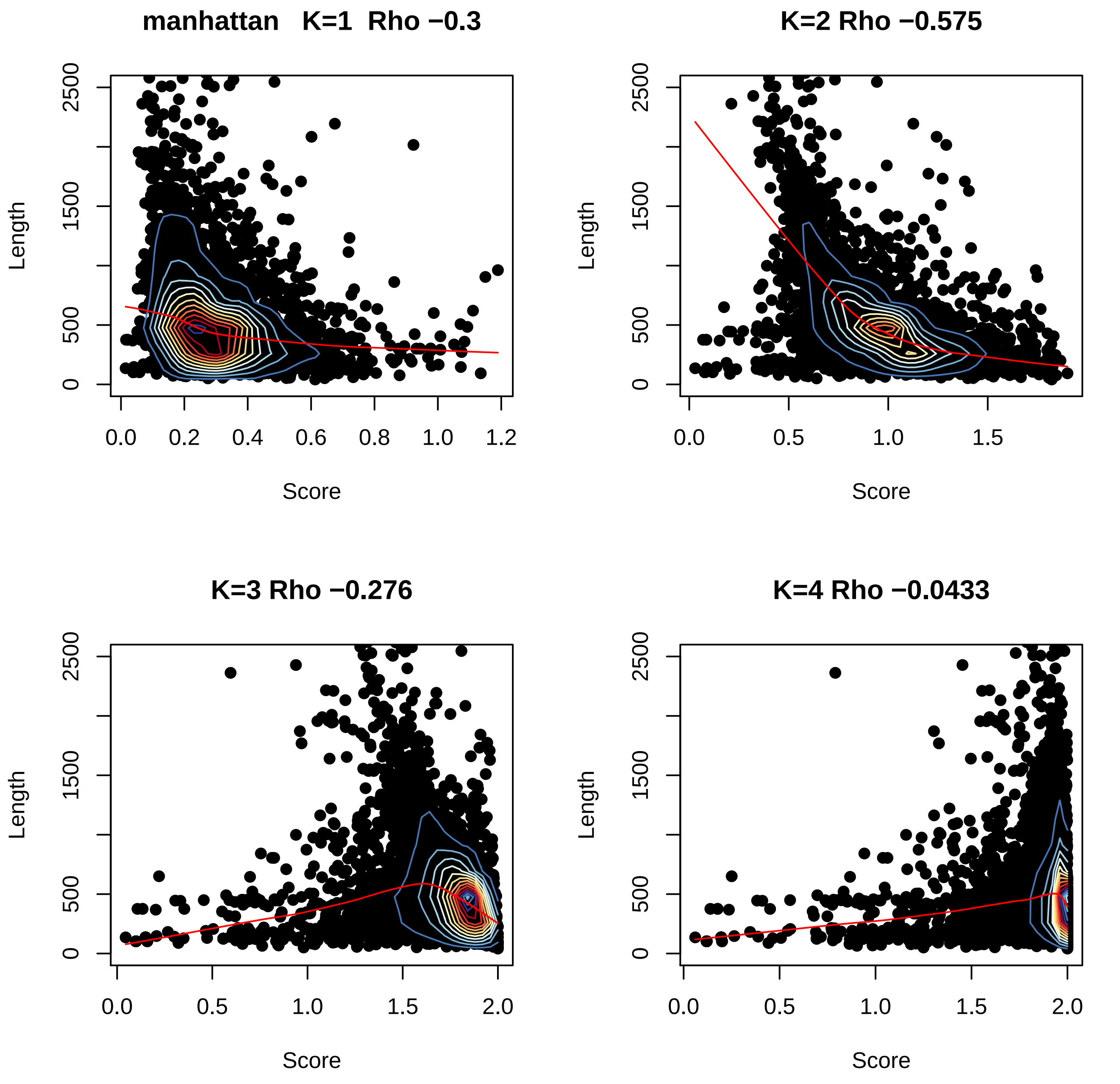
manhattan K=1 Rho −0.3
K=2 Rho −0.575
K=3 Rho −0.276
K=4 Rho −0.0433
Length
Score
0
500
1500
2500
0.0
0.2
0.4
0.6
0.8
1.0
1.2
0.5
1.5
2.0

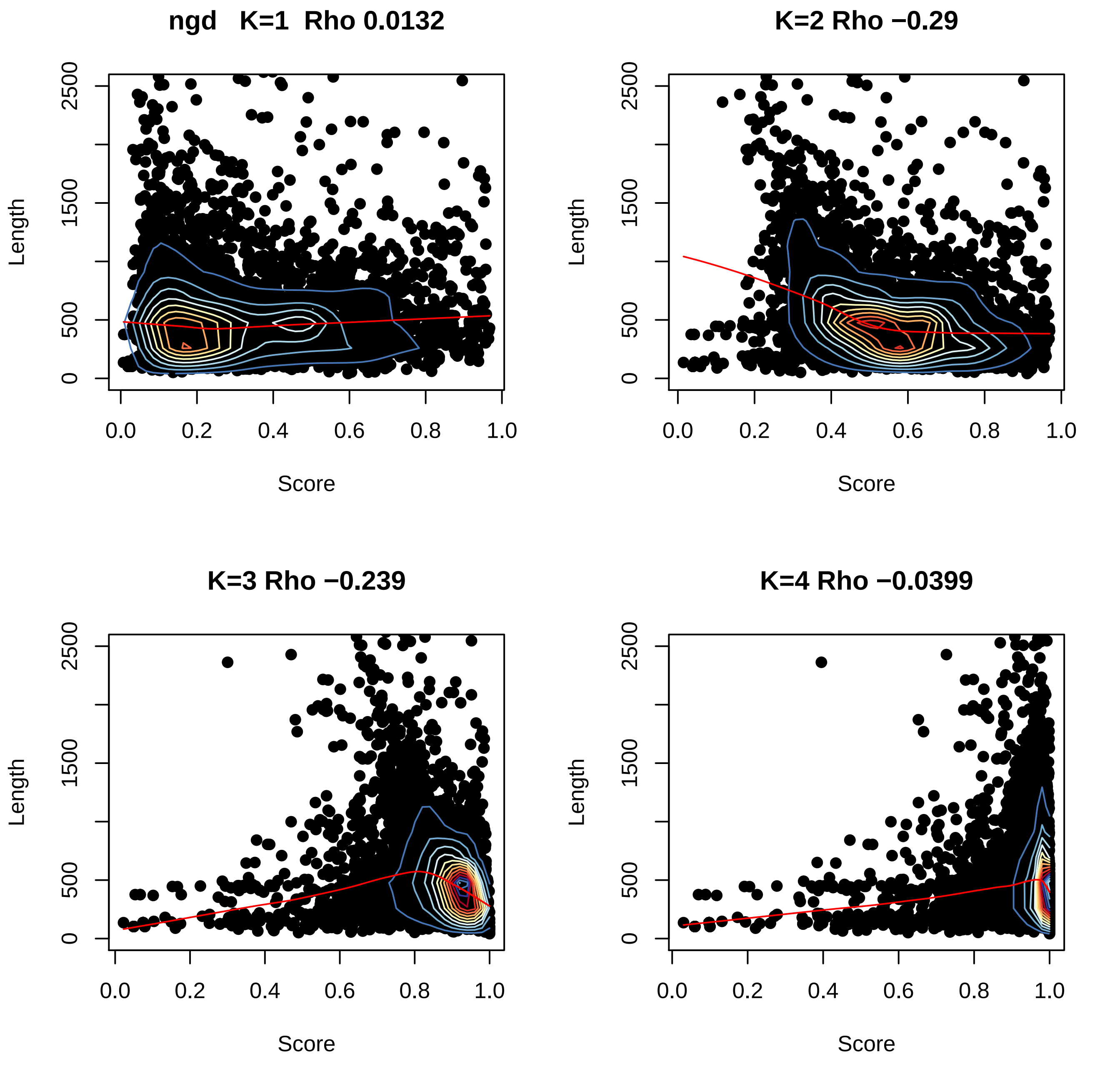

ngd K=1 Rho 0.0132
K=2 Rho −0.29
K=3 Rho −0.239
K=4 Rho −0.0399
Length
Score
0
500
1500
2500
0.0
0.2
0.4
0.6
0.8
1.0

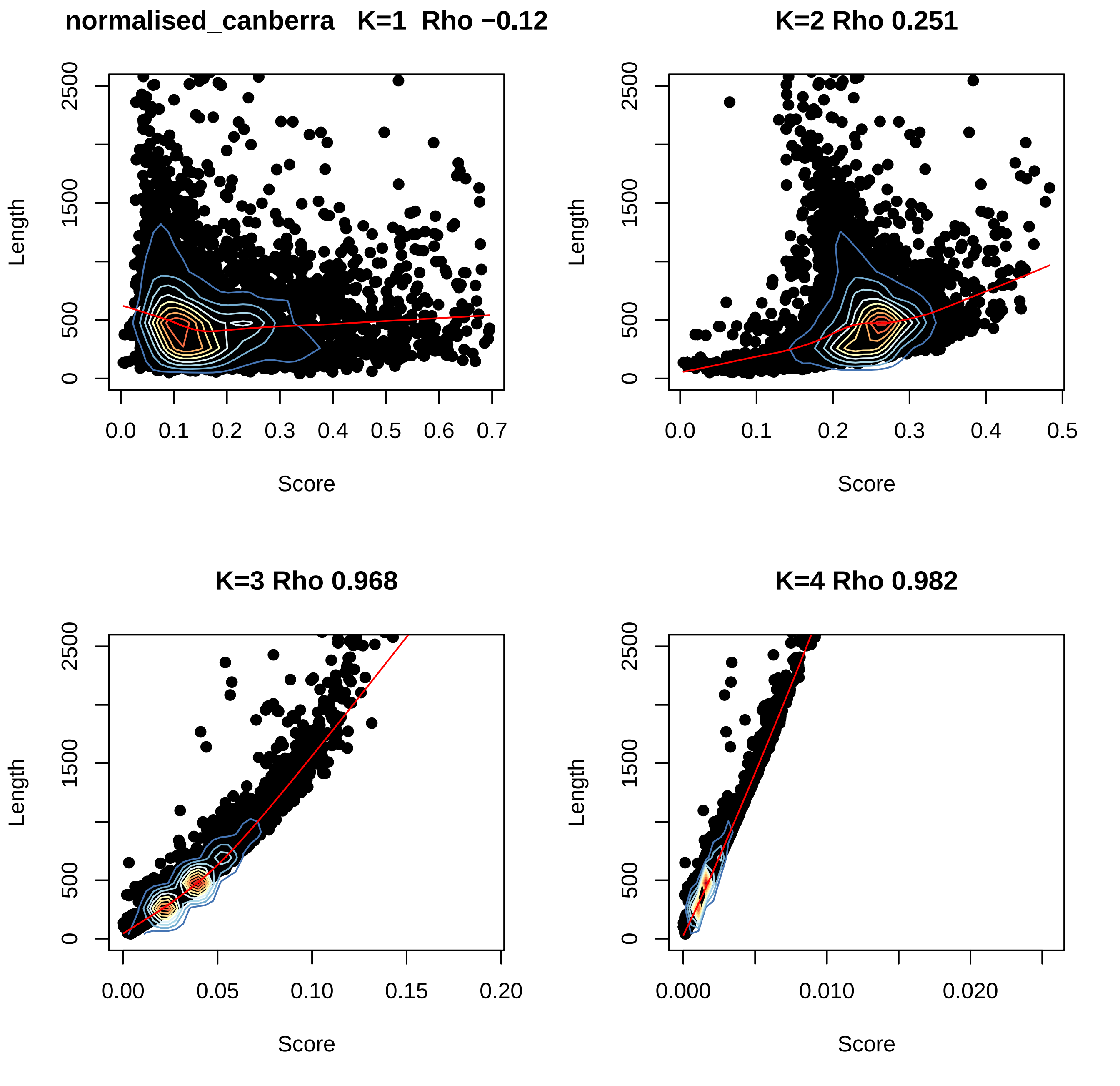

normalised_canberra K=1 Rho −0.12
K=2 Rho 0.251
K=3 Rho 0.968
K=4 Rho 0.982
Length
Score
0
500
1500
2500
0.0
0.1
0.2
0.3
0.4
0.5
0.6
0.7
0.00
0.05
0.10
0.15
0.20
0.000
0.010
0.020
