# Supplementary material for: Interpreting alignment-free sequence comparison: what makes a score a good score?
Source: NAR Genom Bioinform. 2022 Sep 5;4(3):lqac062. doi: 10.1093/nargab/lqac062 (PMC9442500; doi:10.1093/nargab/lqac062)
Supplement: lqac062_Supplemental_Files [file lqac062_supplemental_files.zip › Supp4_Figure11extras.pdf]

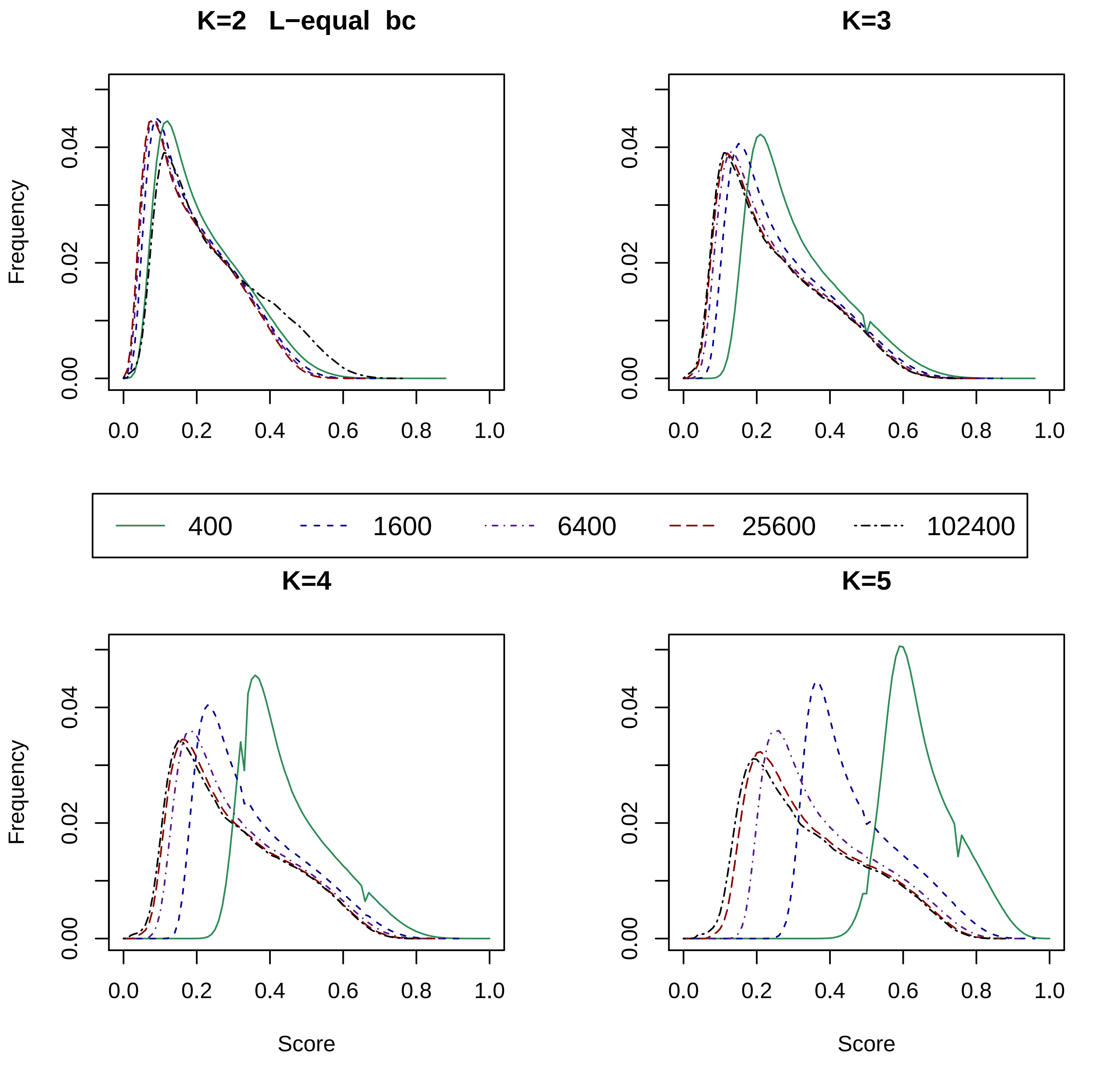
K=2 L−equal bc
K=3
K=4
K=5
Frequency
Score
0.00
0.02
0.04
0.0
0.2
0.4
0.6
0.8
1.0
400
1600
6400
25600
102400

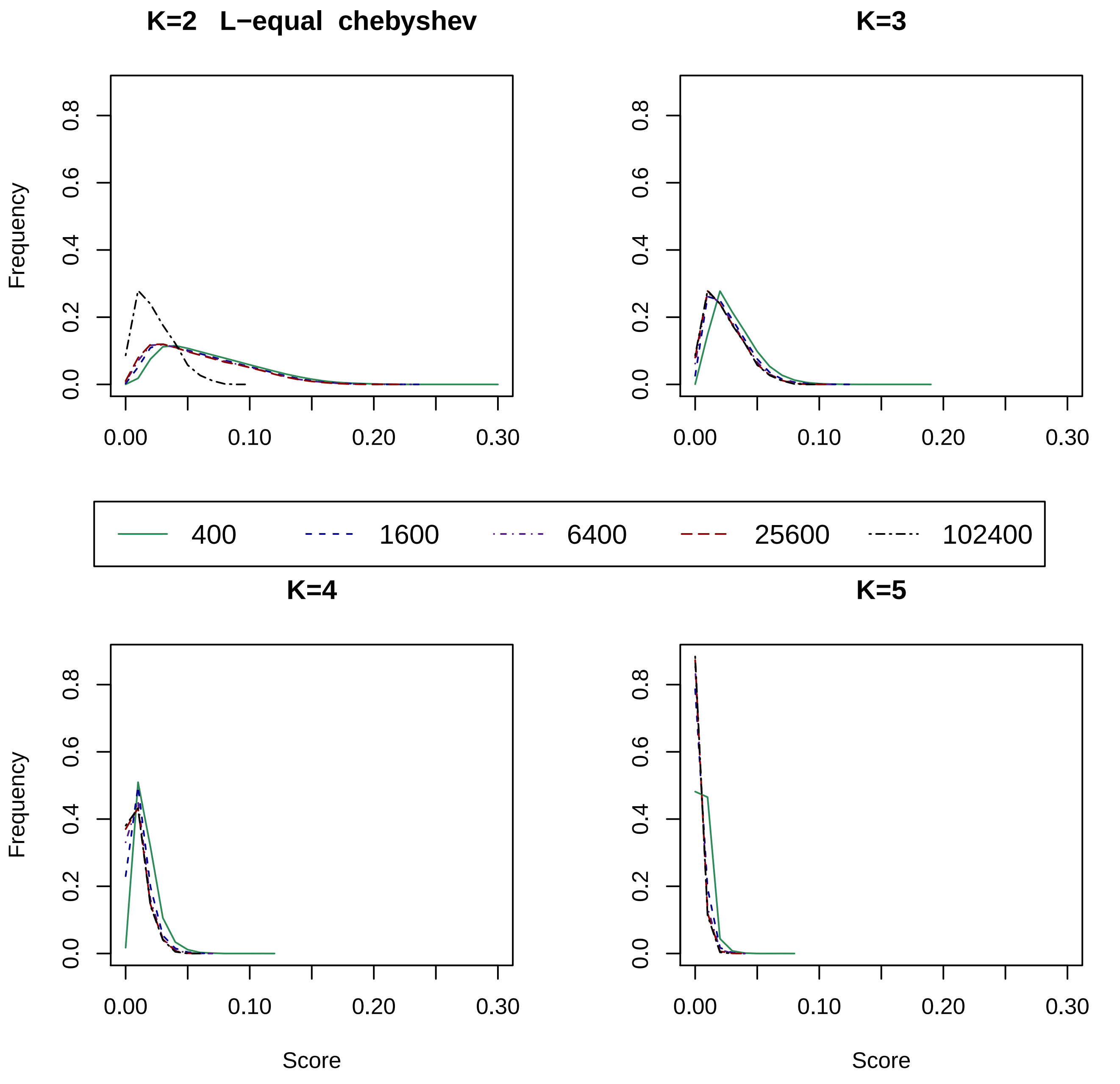

K=2 L−equal chebyshev
K=3
K=4
K=5
Frequency
Score
0.0
0.2
0.4
0.6
0.8
0.00
0.10
0.20
0.30
400
1600
6400
25600
102400

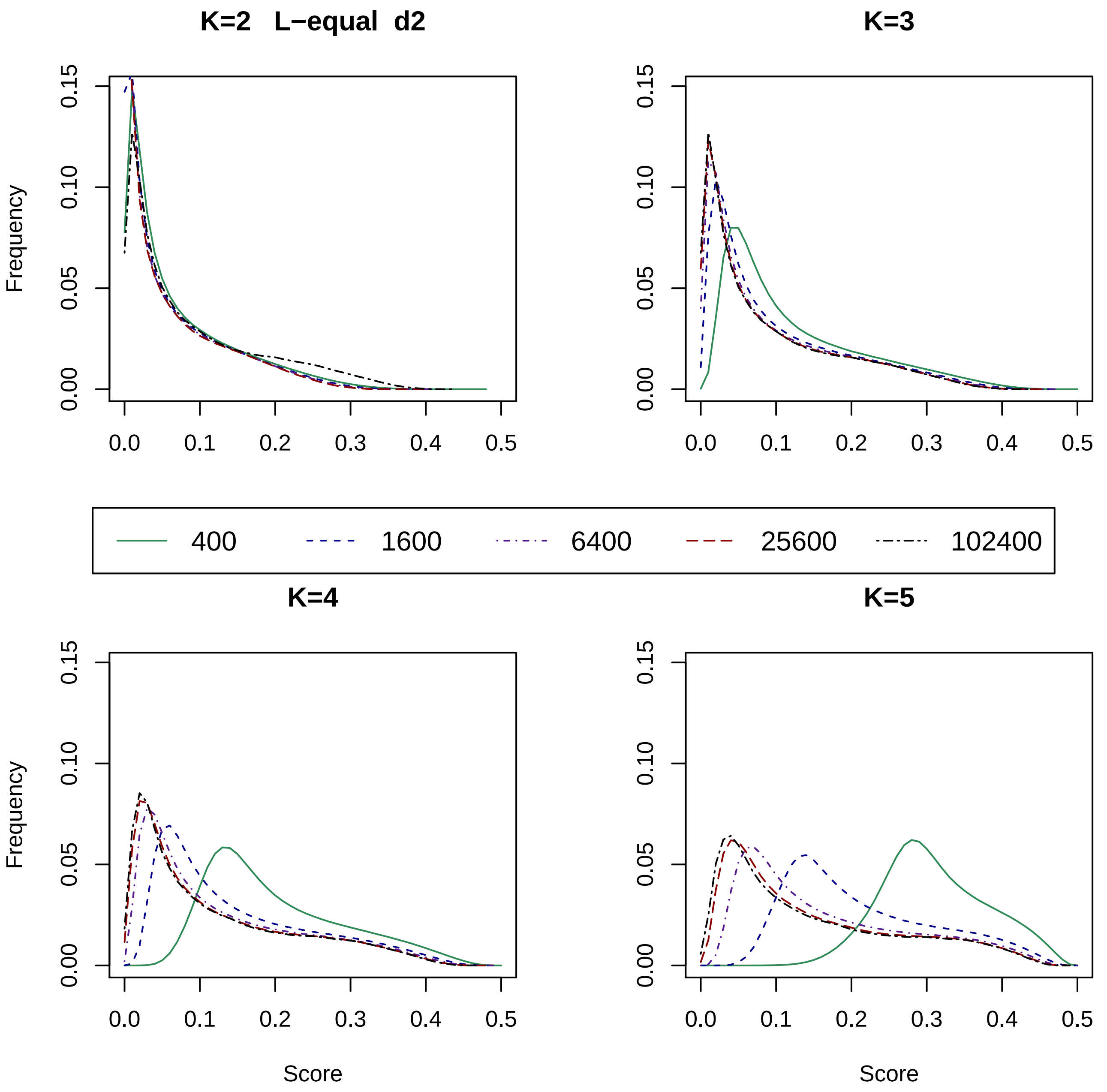

K=2 L−equal d2
K=3
K=4
K=5
Frequency
Score
0.00
0.05
0.10
0.15
0.0
0.1
0.2
0.3
0.4
0.5
400
1600
6400
25600
102400

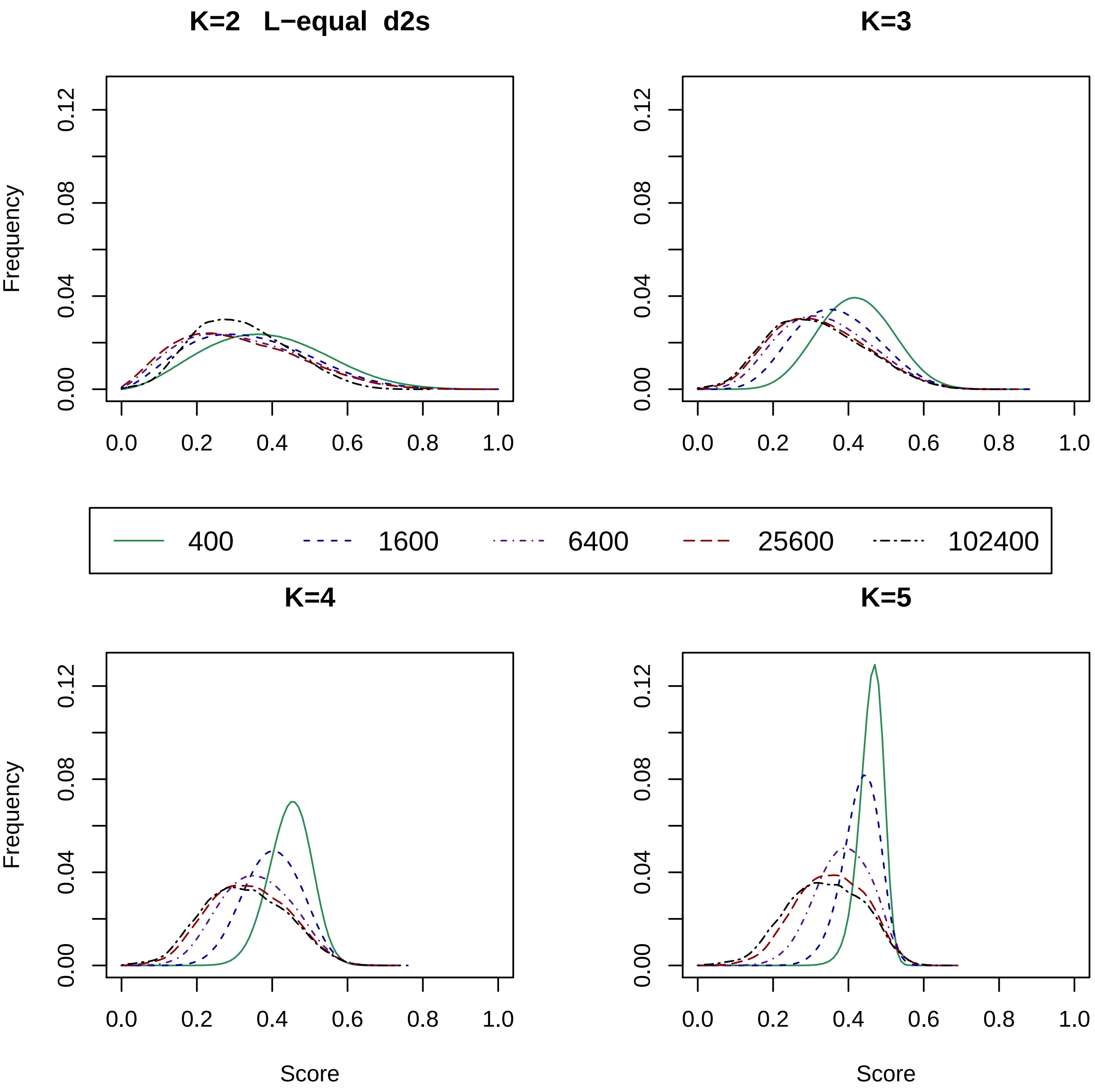
K=2 L−equal d2s
K=3
K=4
K=5
Frequency
Score
0.00
0.04
0.08
0.12
0.0
0.2
0.4
0.6
0.8
1.0
400
1600
6400
25600
102400

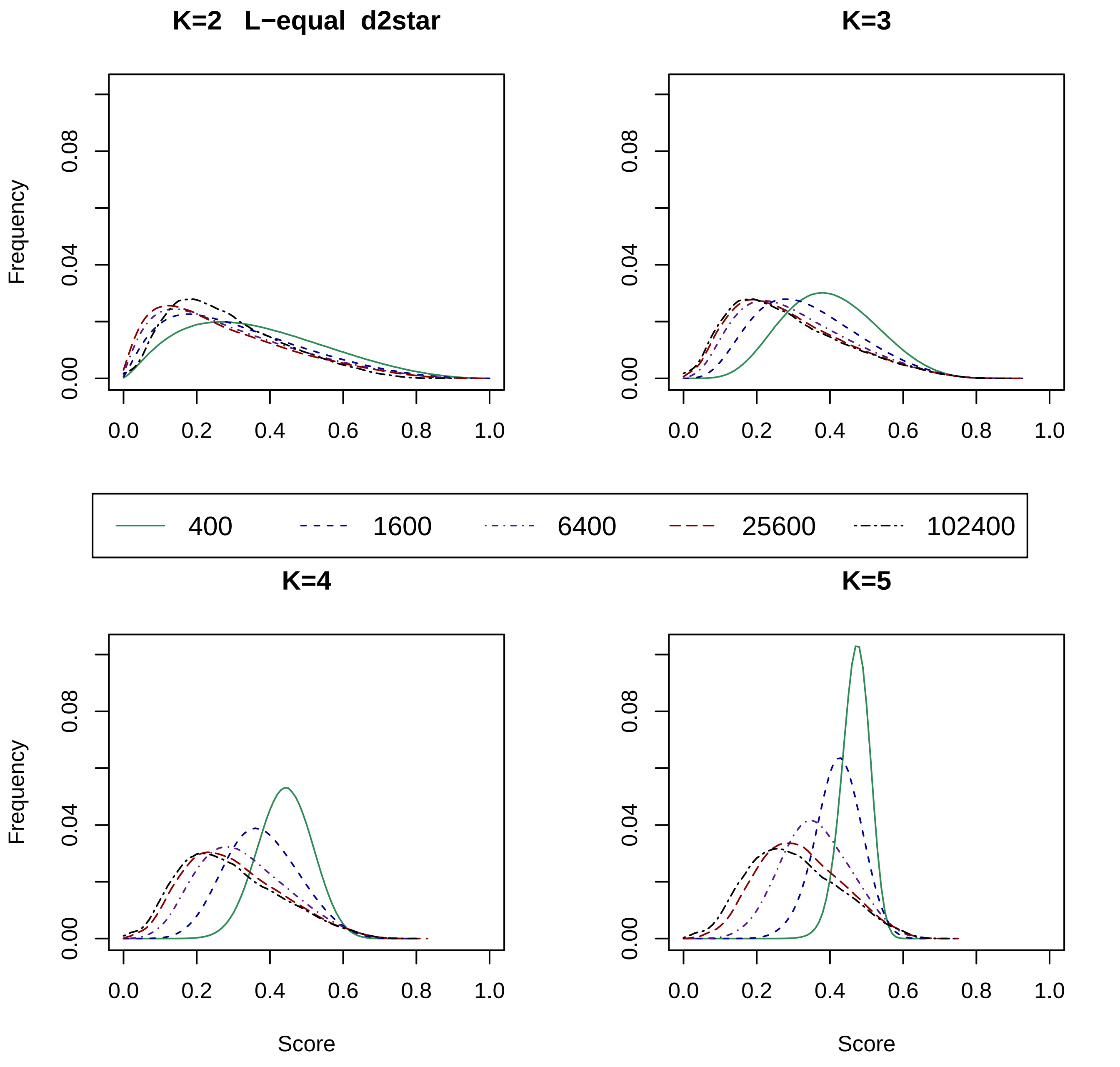

K=2 L−equal d2star
K=3
K=4
K=5
Frequency
Score
0.00
0.04
0.08
0.0
0.2
0.4
0.6
0.8
1.0
400
1600
6400
25600
102400

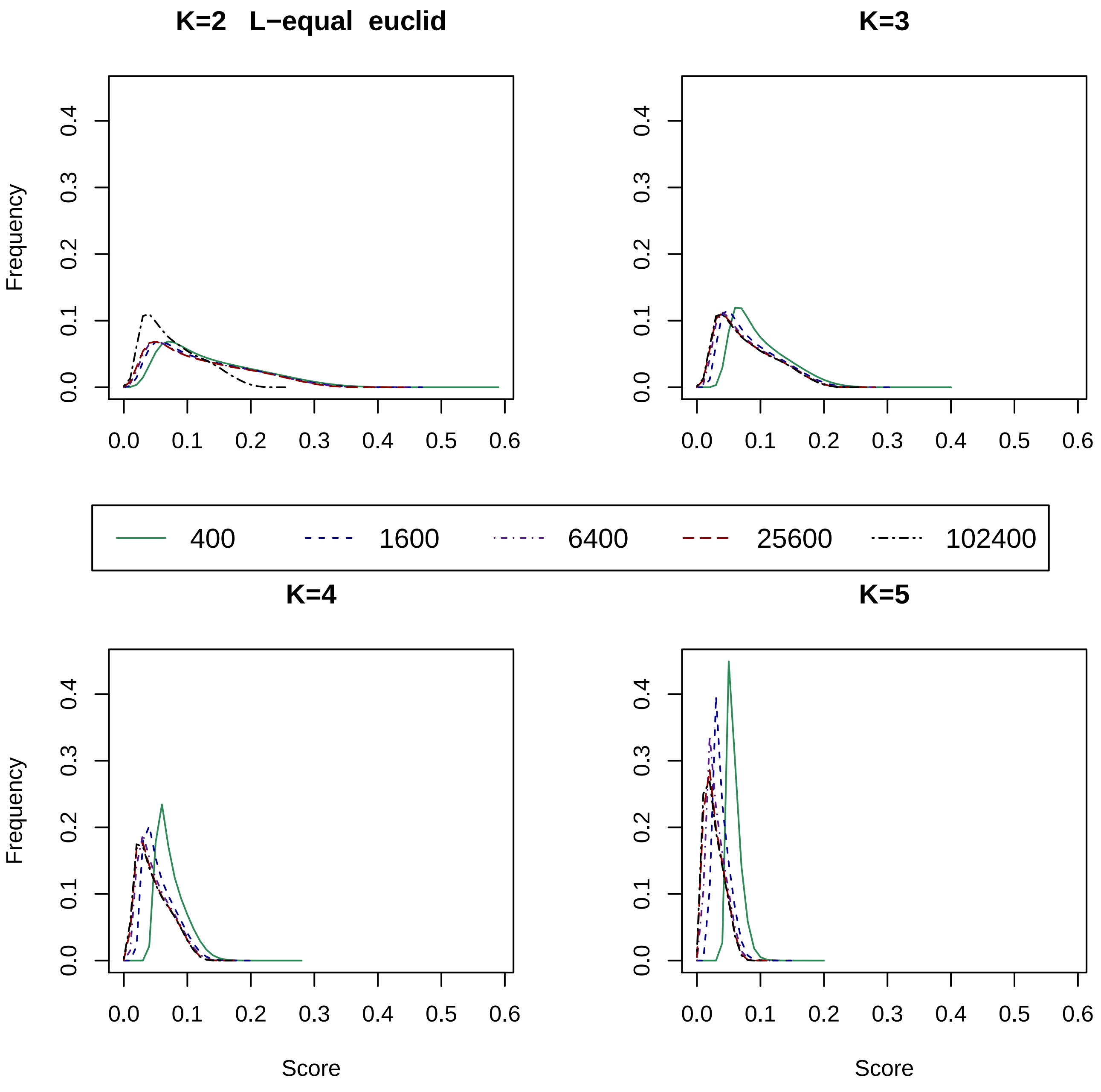
K=2 L−equal euclid
K=3
K=4
K=5
Frequency
Score
0.0
0.1
0.2
0.3
0.4
0.5
0.6
400
1600
6400
25600
102400

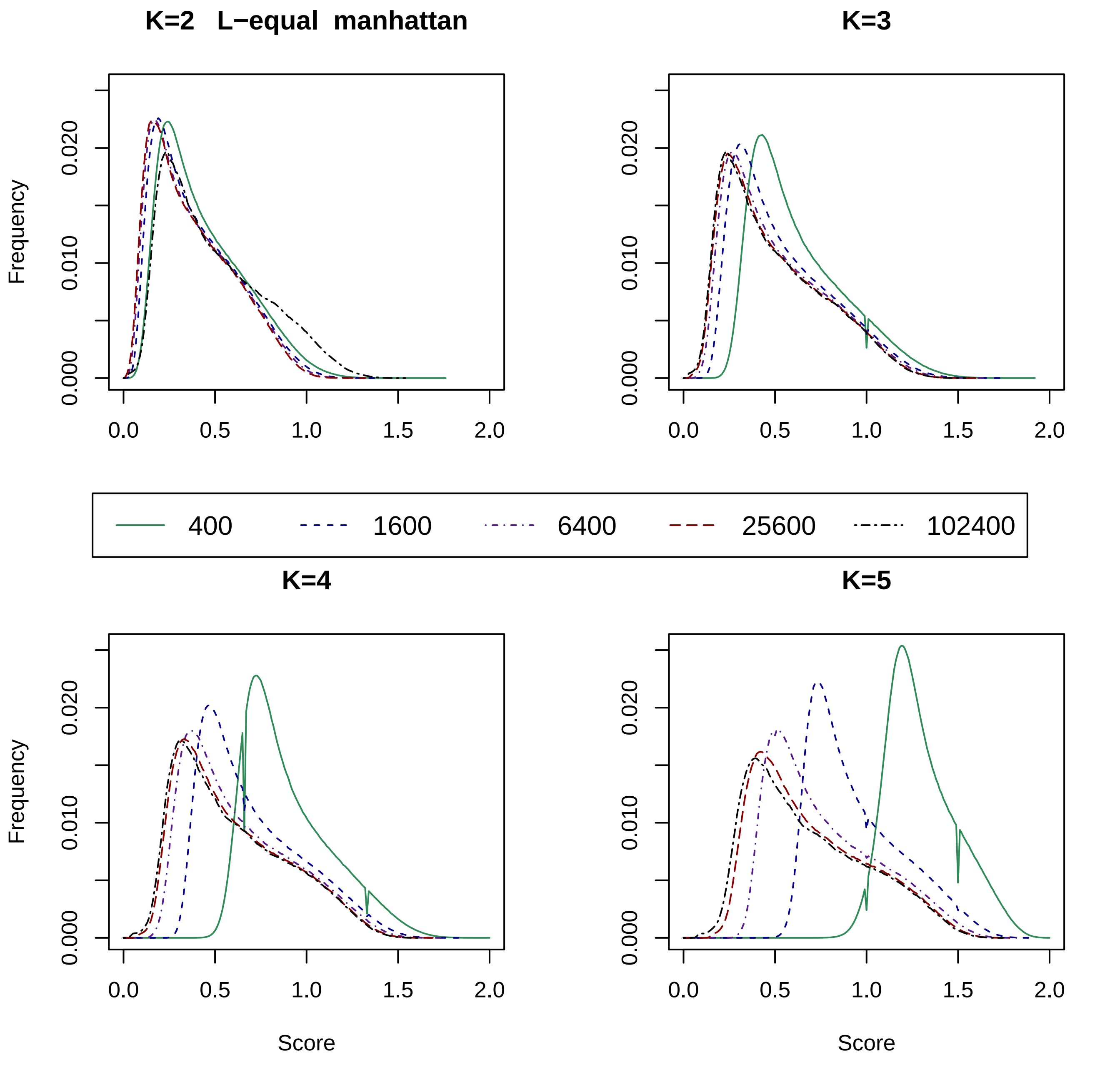

K=2 L−equal manhattan
K=3
K=4
K=5
Frequency
Score
0.000
0.010
0.020
0.0
0.5
1.0
1.5
2.0
400
1600
6400
25600
102400

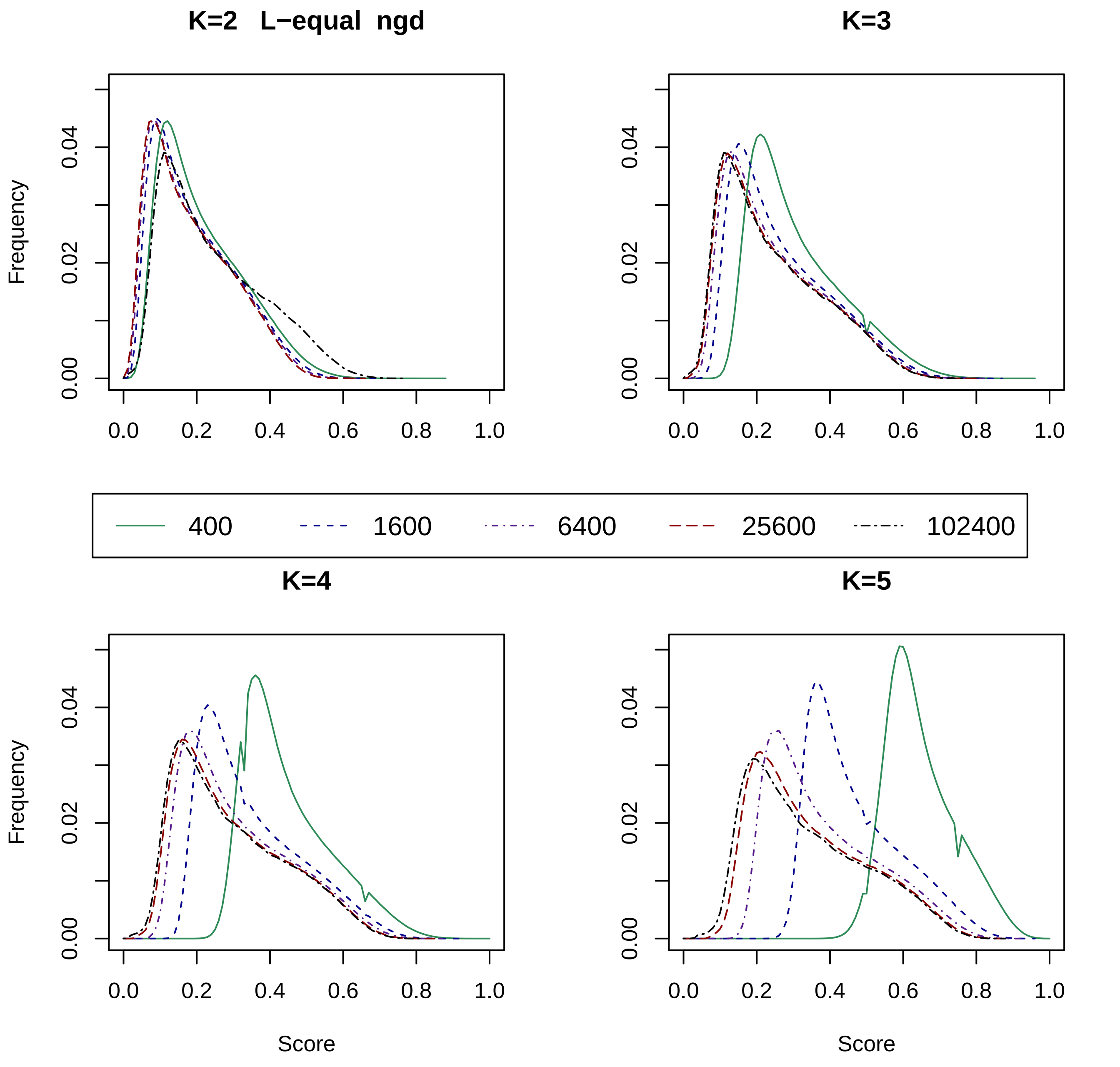

K=2 L−equal ngd
K=3
K=4
K=5
Frequency
Score
0.00
0.02
0.04
0.0
0.2
0.4
0.6
0.8
1.0
400
1600
6400
25600
102400

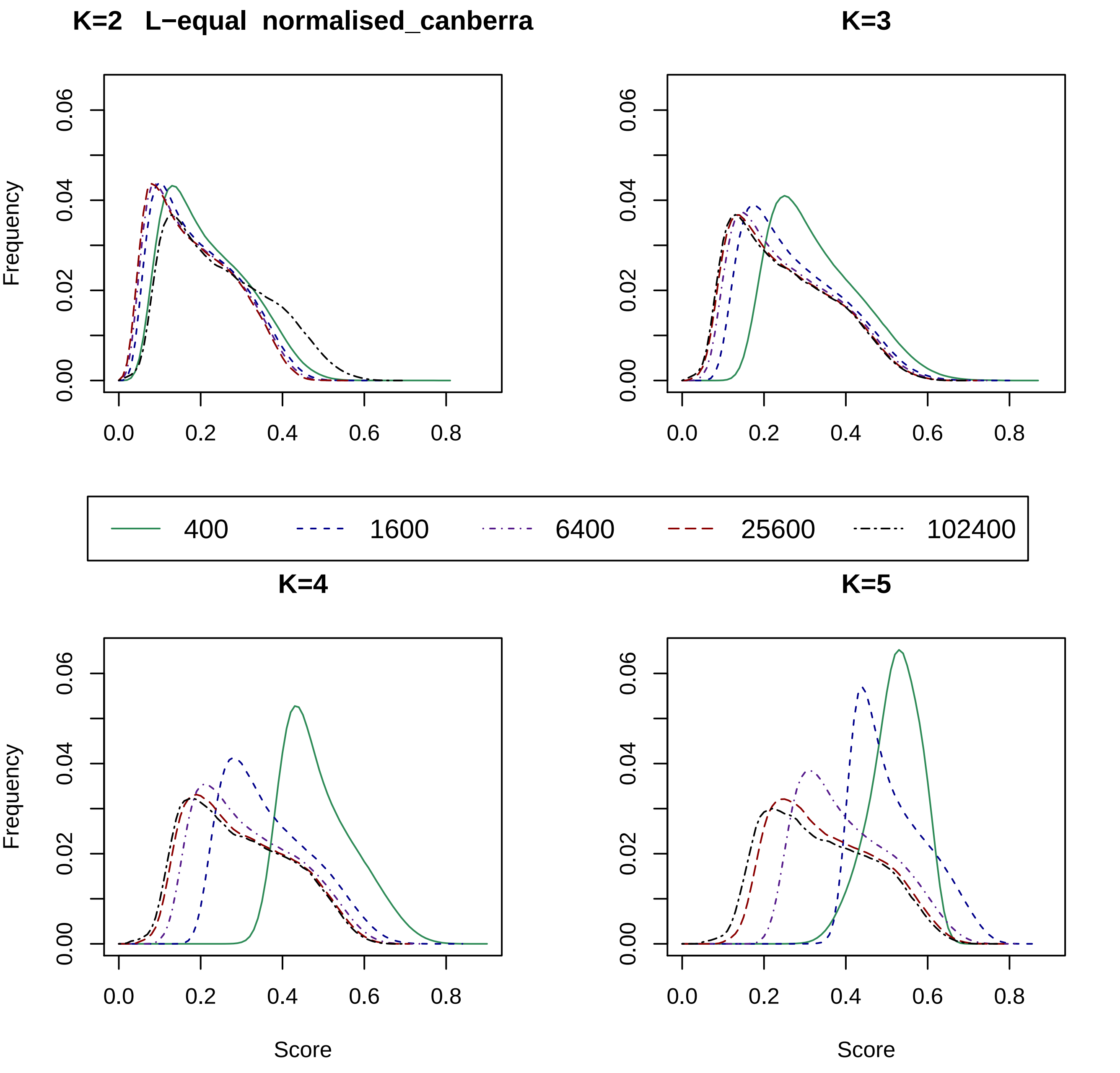
K=2 L−equal normalised_canberra
K=3
K=4
K=5
Frequency
Score
0.00
0.02
0.04
0.06
0.0
0.2
0.4
0.6
0.8
400
1600
6400
25600
102400

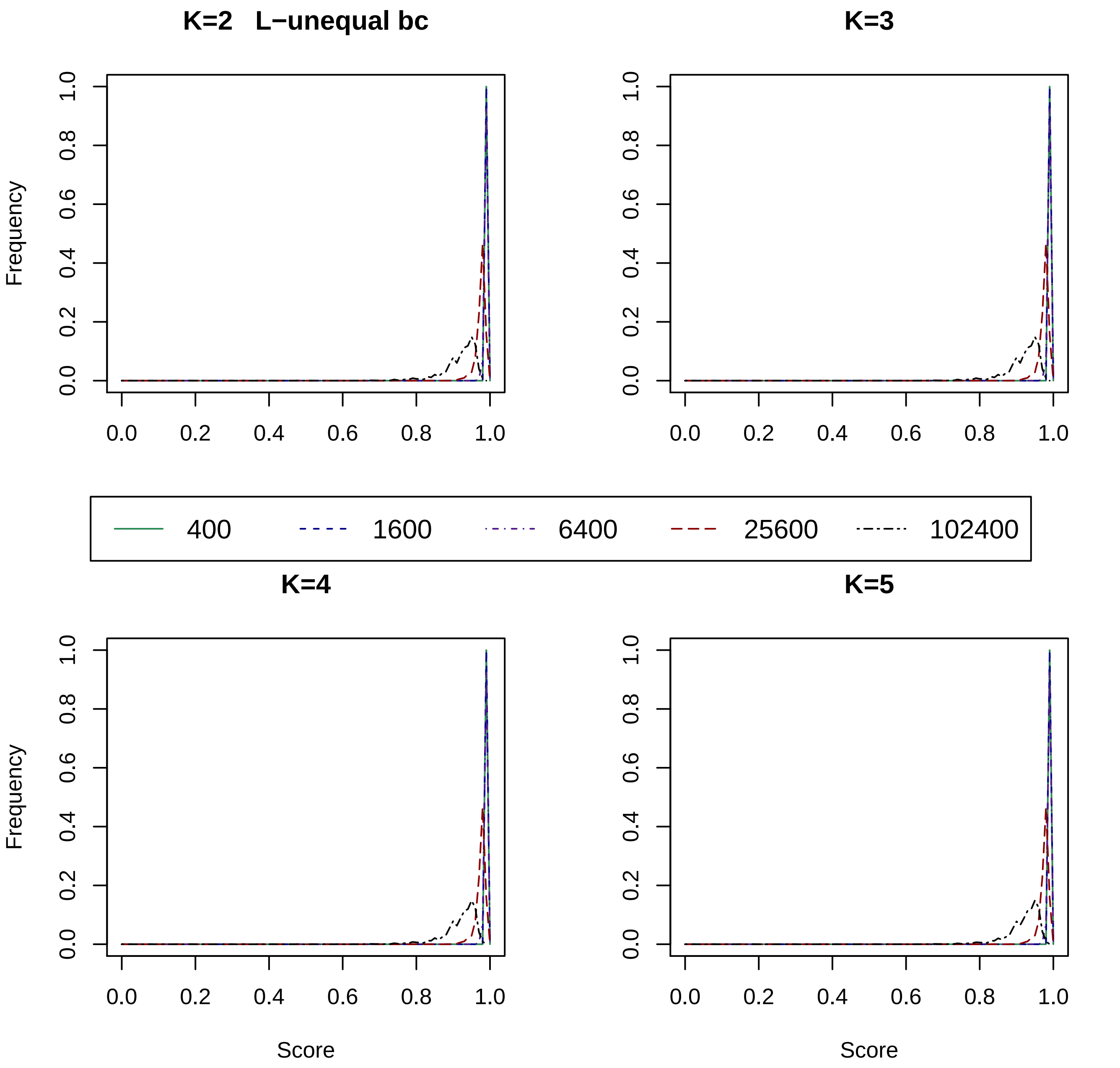

K=2 L−unequal bc
K=3
K=4
K=5
Frequency
Score
0.0
0.2
0.4
0.6
0.8
1.0
400
1600
6400
25600
102400

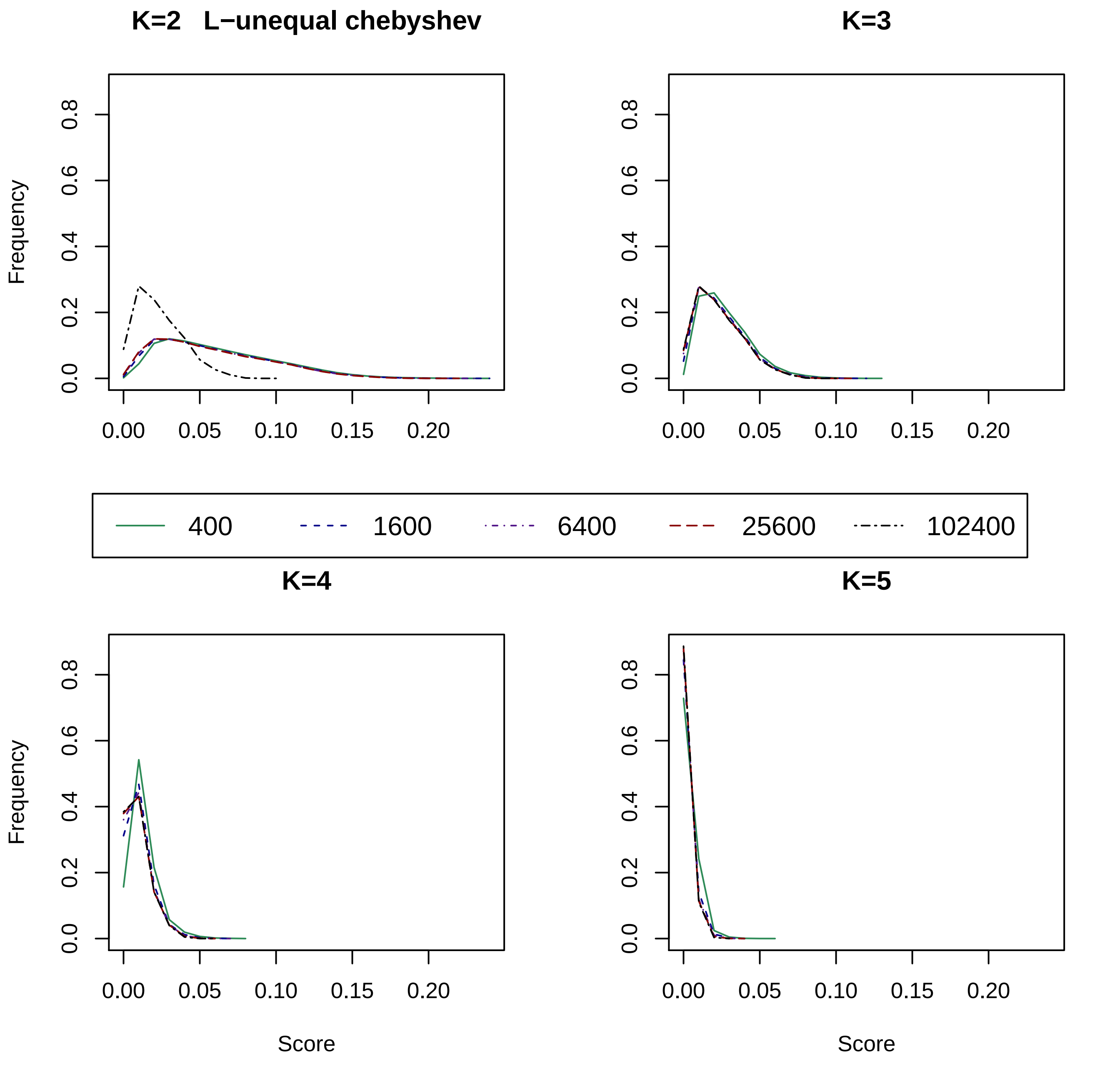

K=2 L−unequal chebyshev
K=3
K=4
K=5
Frequency
Score
0.0
0.2
0.4
0.6
0.8
0.00
0.05
0.10
0.15
0.20
400
1600
6400
25600
102400

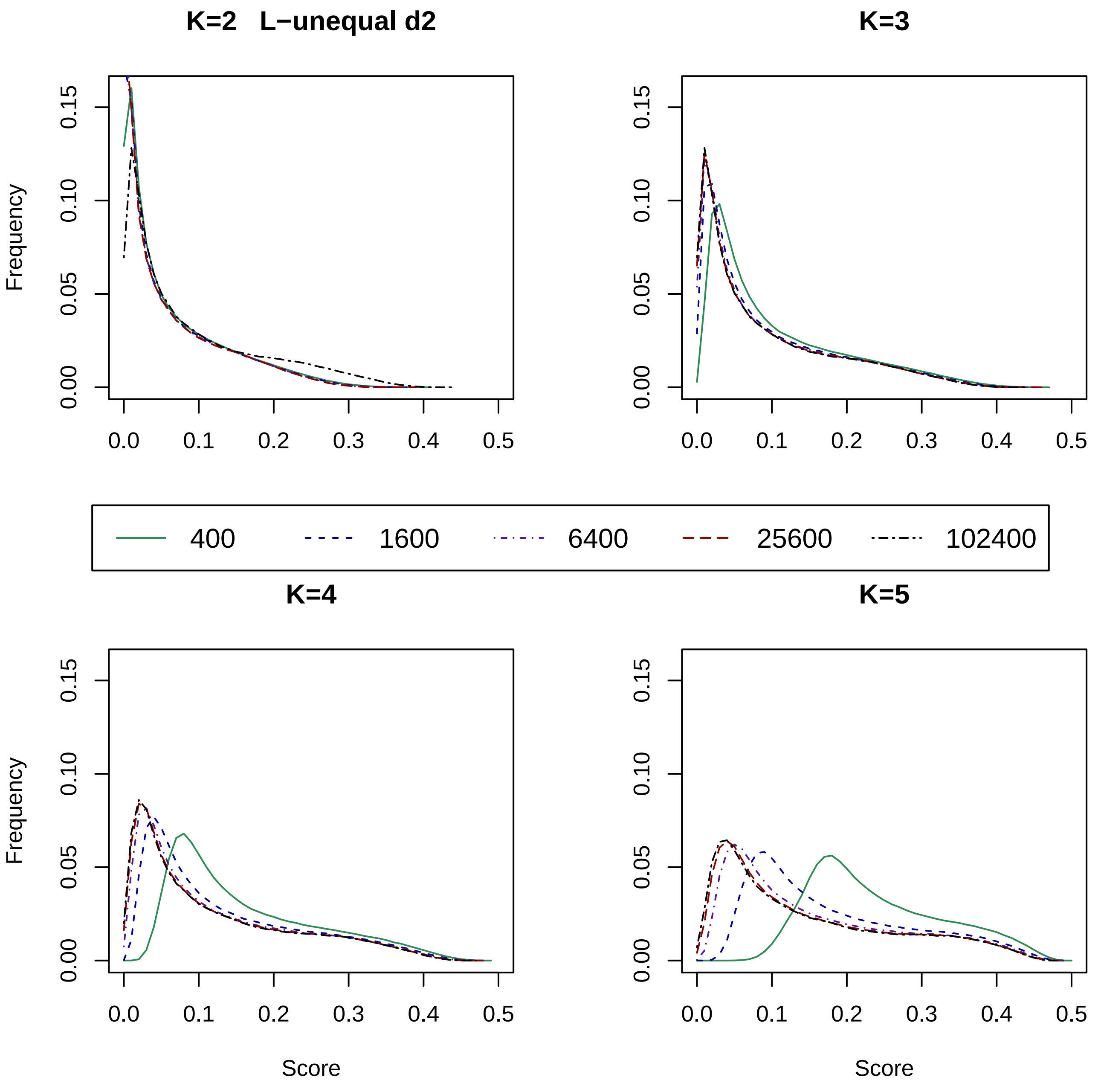

K=2 L−unequal d2
K=3
K=4
K=5
Frequency
Score
0.00
0.05
0.10
0.15
0.0
0.1
0.2
0.3
0.4
0.5
400
1600
6400
25600
102400

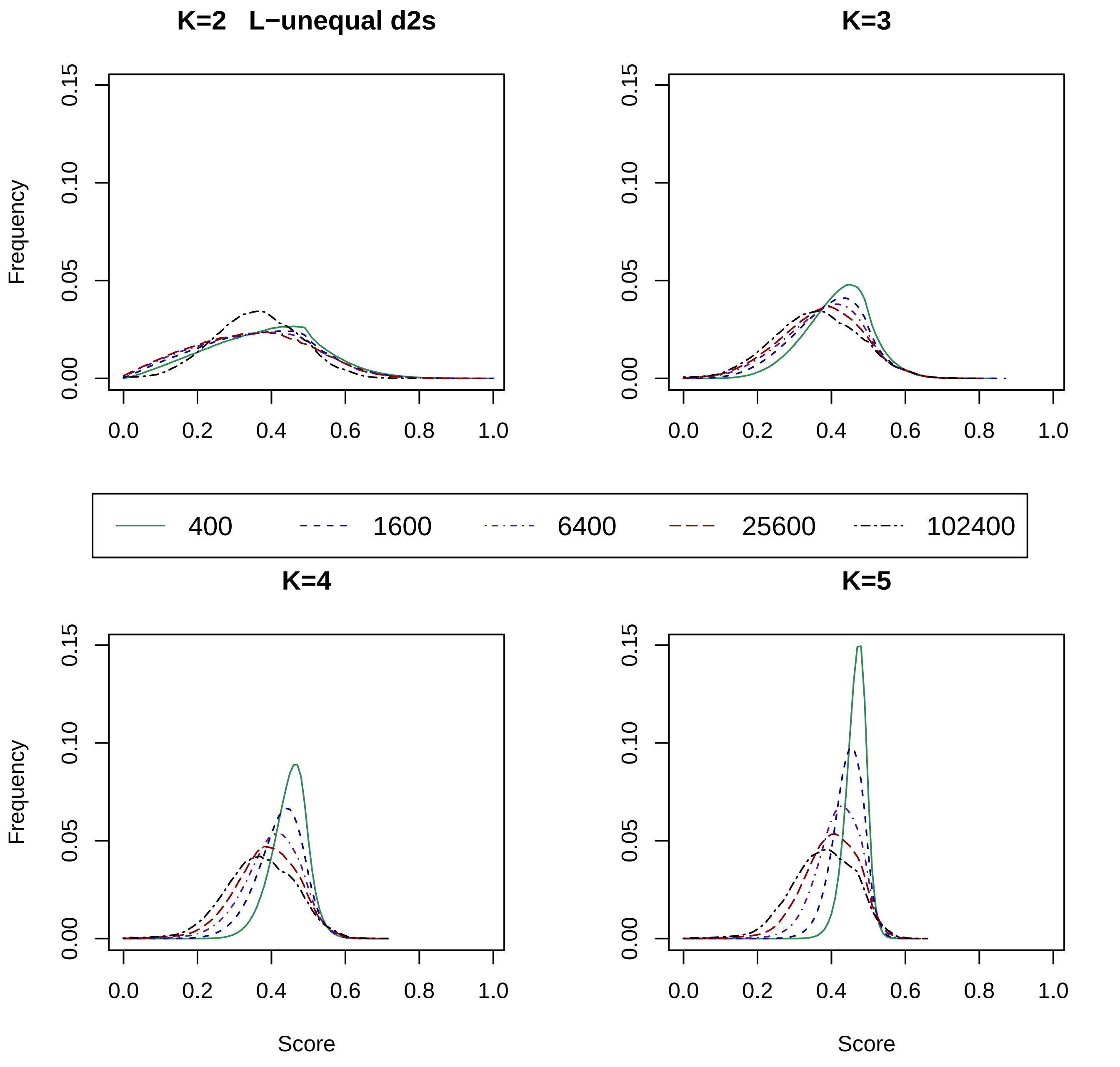

K=2 L−unequal d2s
K=3
K=4
K=5
Frequency
Score
0.00
0.05
0.10
0.15
0.0
0.2
0.4
0.6
0.8
1.0
400
1600
6400
25600
102400

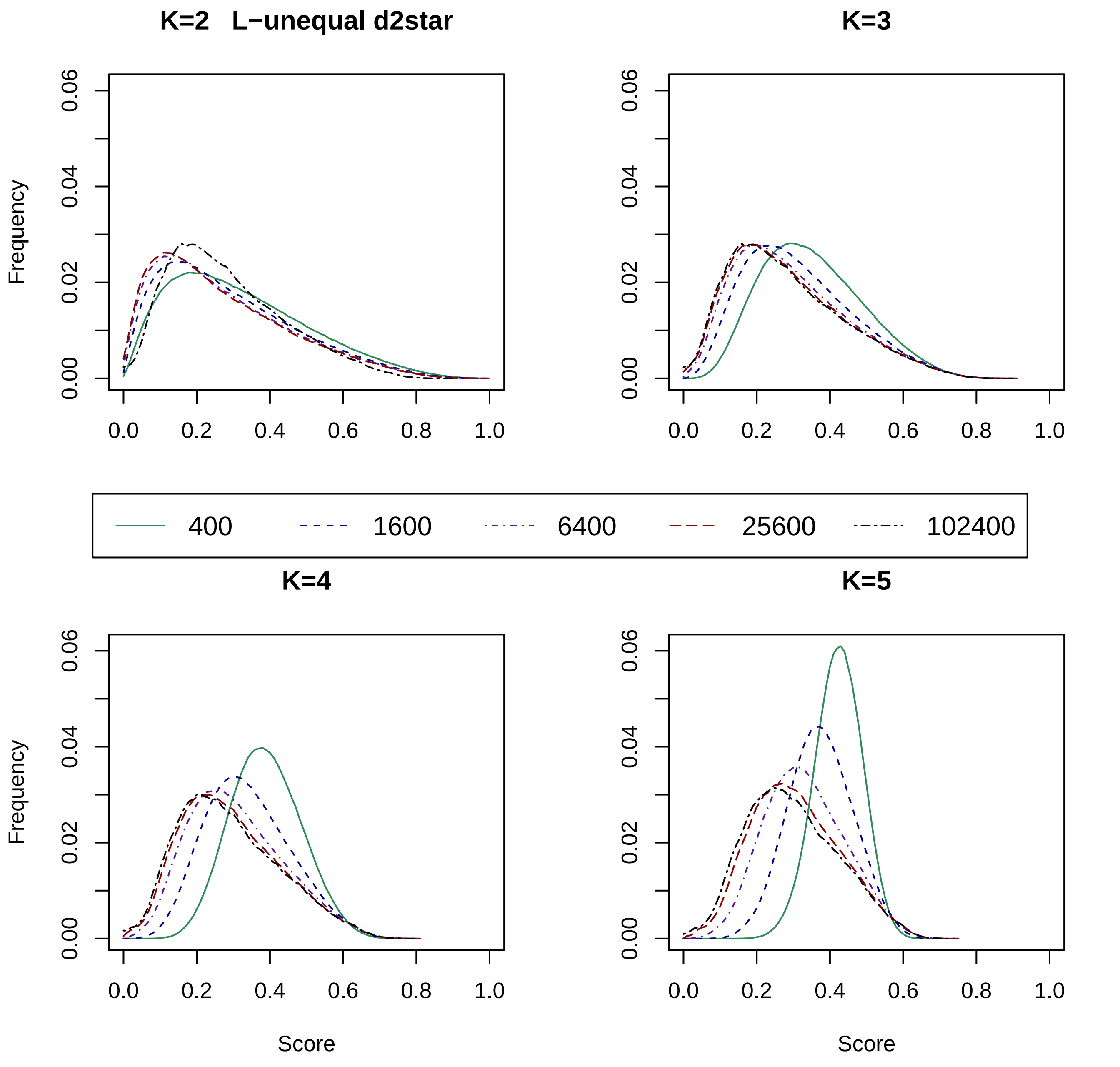
K=2 L−unequal d2star
K=3
K=4
K=5
Frequency
Score
0.00
0.02
0.04
0.06
0.0
0.2
0.4
0.6
0.8
1.0
400
1600
6400
25600
102400

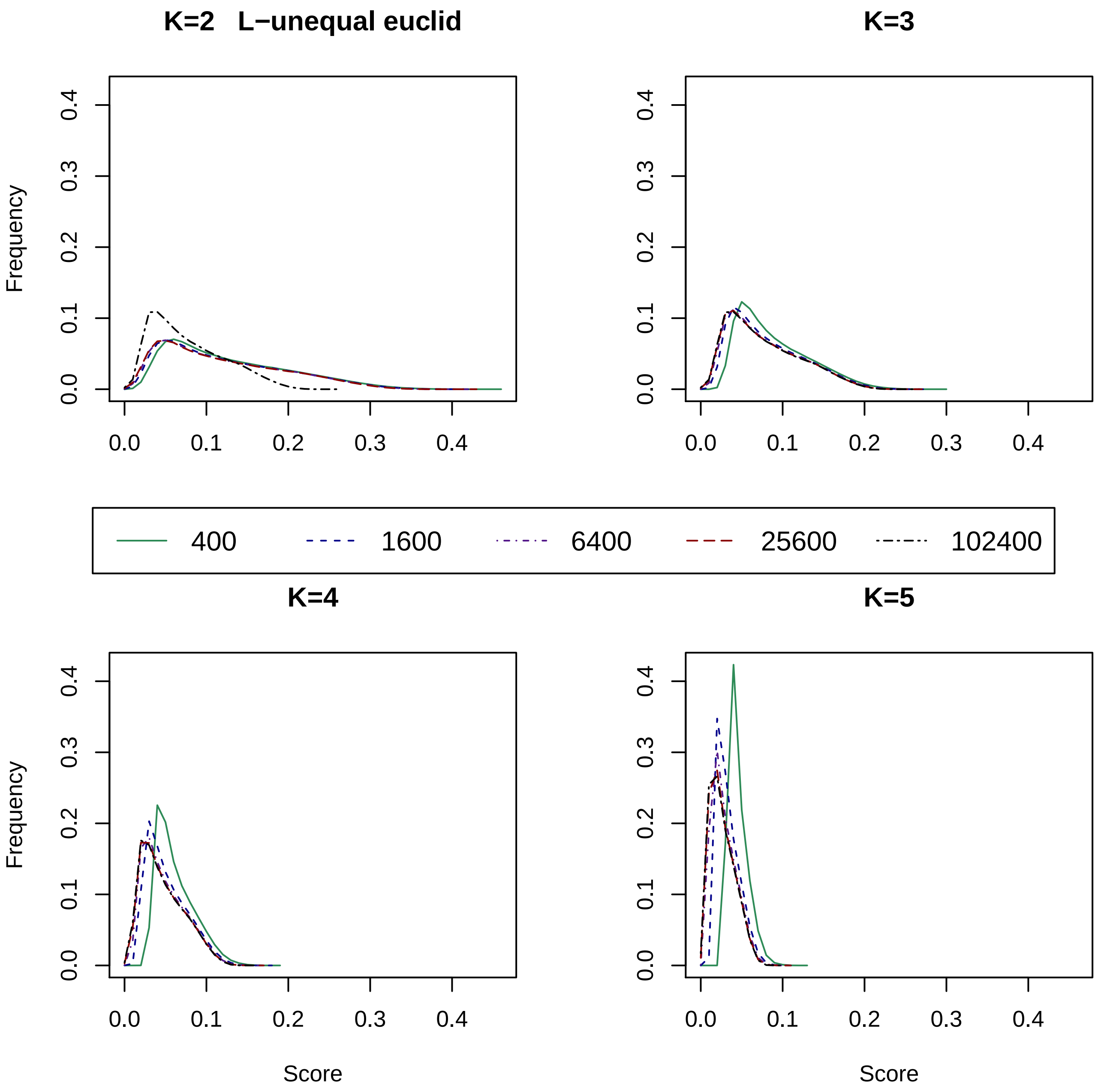
K=2 L−unequal euclid
K=3
K=4
K=5
Frequency
Score
400
1600
6400
25600
102400
0.0
0.1
0.2
0.3
0.4

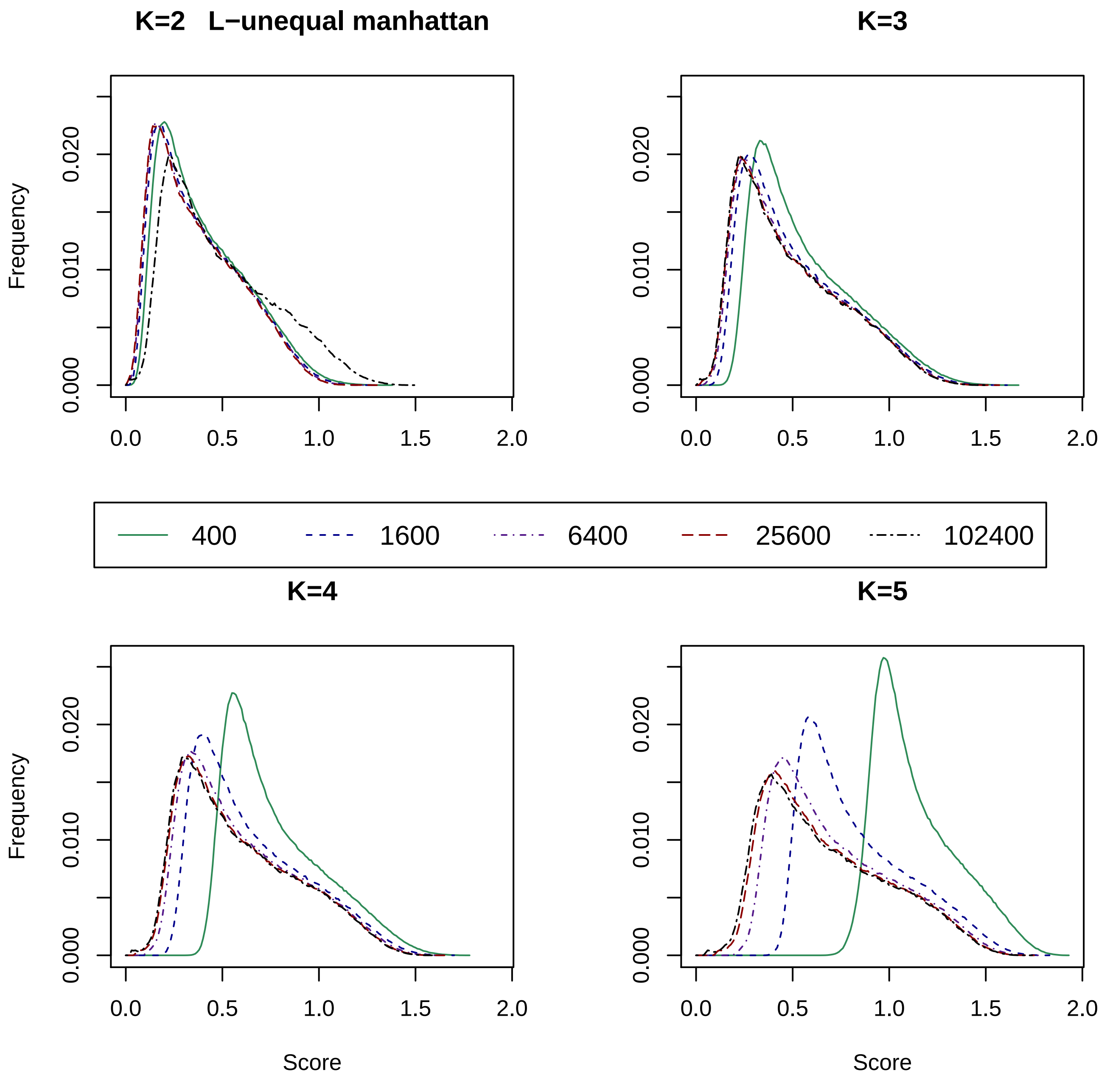

K=2 L−unequal manhattan
K=3
K=4
K=5
Frequency
Score
400
1600
6400
25600
102400

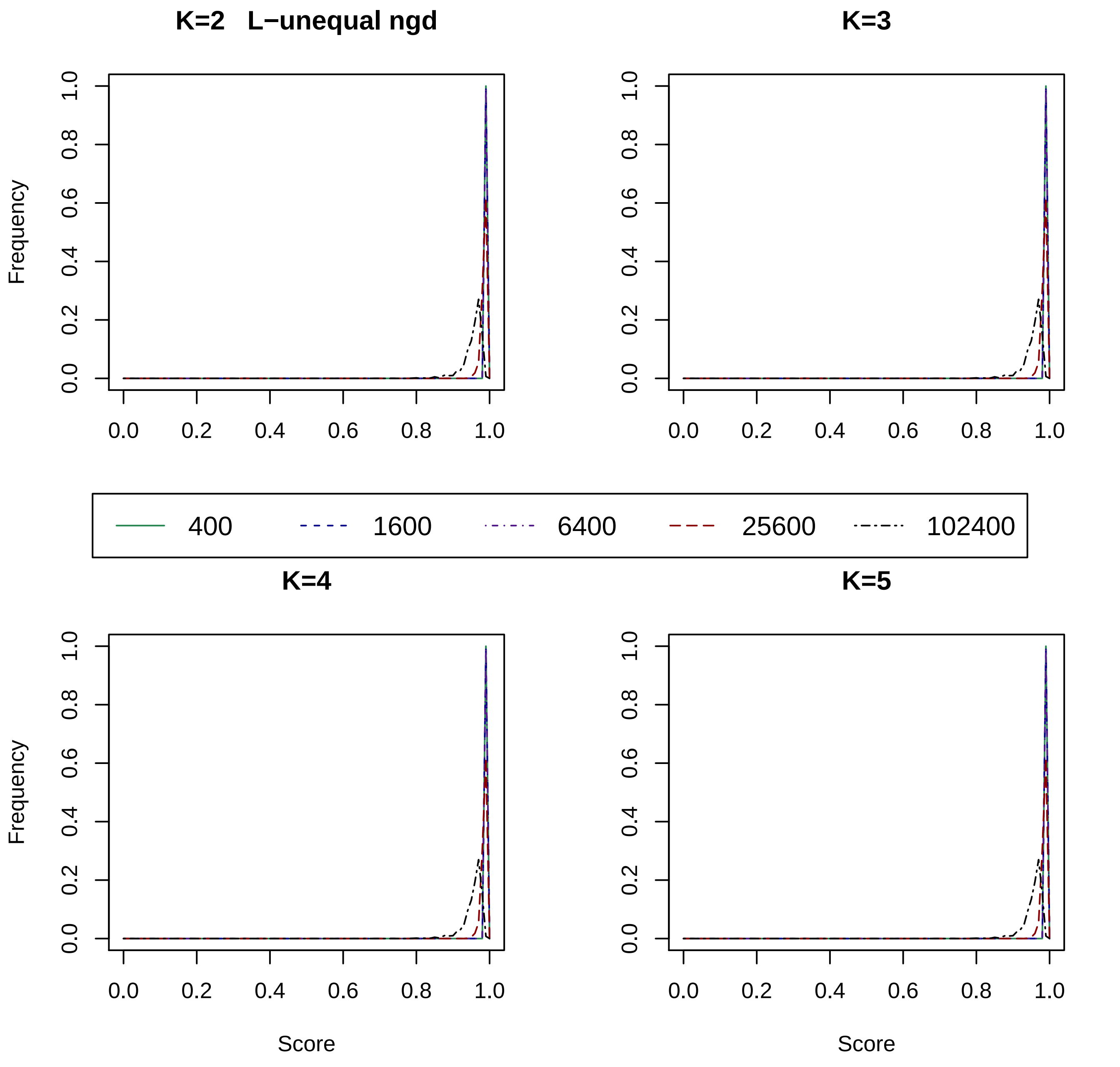

K=2 L−unequal ngd
K=3
K=4
K=5
Frequency
Score
0.0
0.2
0.4
0.6
0.8
1.0
400
1600
6400
25600
102400

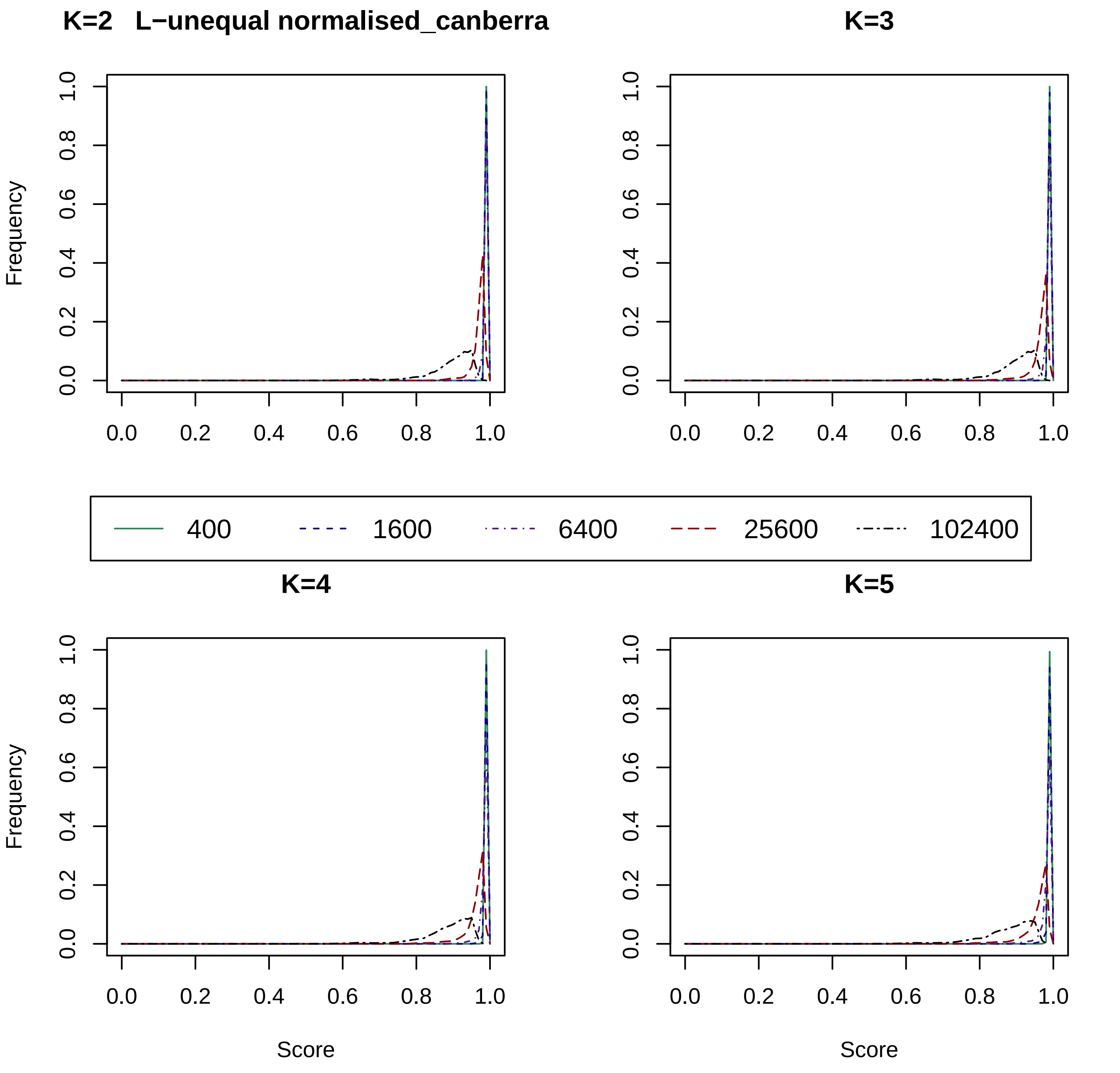
K=2 L−unequal normalised_canberra
K=3
K=4
K=5
Frequency
Score
0.0
0.2
0.4
0.6
0.8
1.0
400
1600
6400
25600
102400
