# Supplementary material for: Interpreting alignment-free sequence comparison: what makes a score a good score?
Source: NAR Genom Bioinform. 2022 Sep 5;4(3):lqac062. doi: 10.1093/nargab/lqac062 (PMC9442500; doi:10.1093/nargab/lqac062)
Supplement: lqac062_Supplemental_Files [file lqac062_supplemental_files.zip › SUPPLEMENTARY_DATA.docx]

SUPPLEMENTARY DATA

Scripts are supplied to make all the figures in this paper (see Data Availability section).

Supplementary File 1: Tables of the data that are shown in Figure 1.

Supplementary File 2: Score distribution histograms for all metrics to expand on Figure 2.

Supplementary File 3: Correlation plots for all metrics to expand on Figure 4.

Supplementary File 4: Score distribution histograms for all metrics to expand on Figure 11.
